# Treatment of chronic hepatitis B naïve patients with a therapeutic vaccine containing HBs and HBc antigens (a randomized, open and treatment controlled phase III clinical trial)

**DOI:** 10.1371/journal.pone.0201236

**Published:** 2018-08-22

**Authors:** Mamun Al Mahtab, Sheikh Mohammad Fazle Akbar, Julio Cesar Aguilar, Gerardo Guillen, Euduaro Penton, Angela Tuero, Osamu Yoshida, Yoichi Hiasa, Morikazu Onji

**Affiliations:** 1 Department of Hepatology, Bangabandhu Sheikh Mujib Medical University, Dhaka, Bangladesh; 2 Department of Medical Sciences, Toshiba General Hospital, Tokyo, Japan; 3 Department of Biomedical Research, Center for Genetic Engineering and Biotechnology, Havana, Cuba; 4 Department of Gastroenterology and Metabology, Ehime University Graduate School of Medicine, Ehime, Japan; 5 Department of Medicine, Sai Sei Kai Imabari Hospital, Imabari, Japan; The Chinese University of Hong Kong, HONG KONG

## Abstract

**Context:**

Current drugs for chronic hepatitis B therapy have a poor efficacy in terms of post-treatment sustained viral suppression and generate important side effects during and after therapy. Therapeutic vaccination with HBV antigens is an attractive alternative to test.

**Objective:**

Evaluating the efficacy of a therapeutic vaccine candidate (designated NASVAC) containing both hepatitis B surface antigen (HBsAg) and core antigen (HBcAg) versus pegylated interferon (Peg-IFN) in naïve chronic hepatitis B patients.

**Design, setting, participants:**

An open phase III, randomised and treatment controlled clinical trial was conducted in a total of 160 CHB patients, allocated into two groups of 80 patients each to receive NASVAC or Peg-IFN. The vaccine formulation comprised 100 μg of each HBsAg and HBcAg, and was administered in 2 cycles of 5 doses. The control group received 48 subcutaneous injections of Peg-IFN alfa 2b, 180 μg per dose, every week, for 48 consecutive weeks.

**Main outcome measure:**

The primary outcome measure was in relation with the proportion of patients showing reduction of the viral load under the limit of detection (250 copies/mL) after 24 weeks of treatment completion.

**Results:**

Sustained control of HBV DNA was significantly more common in NASVAC group (p<0.05) at 24 weeks of follow up. NASVAC-induced increases of alanine aminotransferases (ALT) were detected in 85% patients after 5 nasal vaccinations, although seen in only 30% of patients receiving Peg-IFN. At the end of treatment (EOT) antiviral effect was comparable in both NASVAC and Peg-IFN groups. Clearance of Hepatitis B e antigen (HBeAg) was also more frequent in NASVAC group compared to Peg-IFN recipients. A lower progression to cirrhosis was found in NASVAC group compared to Peg-IFN group.

**Conclusion:**

Nasvac induced a superior reduction of the viral load under the limit of detection compared to Peg-IFN treatment. It is a safe and efficacious finite alternative of antiviral treatment for CHB patients.

**Trial registration:**

ClinicalTrials.gov NCT 01374308.

## Introduction

Chronic hepatitis B virus (HBV) infection represents a major global public health problem. About 250 million people worldwide are chronically infected with HBV and most of them are infected at birth or during childhood [1]. Epidemiological studies have shown that about 20–25% of individuals infected with HBV as children are prone to develop chronic hepatitis B (CHB), and its complications like liver cirrhosis (LC) and hepatocellular carcinoma (HCC) [2]. World Health Organization estimates indicate that annual deaths due to HBV are more than 0.68 million [3]. A proper management of CHB patients may reduce progression to LC and HCC, and consequently HBV-related mortality.

At present, two types of treatments have been approved: (1) interferon (IFN) that is endowed with antiviral and immune modulatory properties and (2) nucleos(t)ide analogs that directly inhibit HBV polymerase and therefore replication [4]. However, these drugs have a poor efficacy in terms of post-treatment sustained viral suppression and IFN generate important side effects during and after therapy [5–7]. Accordingly, various approaches are pursued to develop more effective therapy regimens.

Although the cellular and molecular mechanisms related to viral clearance and liver damage are poorly elucidated, it has been demonstrated that efficient host immunity is required for the control of both persistent HBV replication and progressive liver damage [8]. To this end polyclonal immune modulators and a wide range of HBV-specific antigen-based immune therapies (vaccine therapy) have been attempted in CHB patients [9–12]. Unfortunately, they did not stand the test of time and so far there has not been any valid immune therapy based on specific active immunization for CHB patients.

Recent studies have indicated that although the concept of immune therapy for CHB remains attractive further modifications in their design are needed to increase efficacy. Both HBsAg and HBcAg-specific immunity seem warranted for control of HBV replication and liver damage [13]. Furthermore ad’hoc regulatory studies are requested to define dose/route of vaccine administration and duration of therapy.

For this sake, a vaccine formulation that contained both hepatitis B surface antigen (HBsAg) and hepatitis B core antigen (HBcAg) (designated NASVAC) was developed [14]. NASVAC was intended to be used via nasal route to induce a broad-based immunity at both mucosal and systemic compartments [15]. Pre-clinical pharmacological and toxicological studies with NASVAC confirmed its safety and efficacy and these have also been documented in HBV transgenic mice which expressed Dane particle, HBV DNA, and HBsAg/HBeAg [16]. A phase I clinical trial carried out in healthy volunteers confirmed the safety and immunogenicity of NASVAC in humans [17]. Subsequently, a phase I/II clinical trial was conducted in 18 CHB patients with further encouraging results in terms of safety and antiviral effect [18]. In the present paper, the results of a phase III clinical trial are reported. The study was aimed at evaluating the safety and efficacy of NASVAC therapeutic vaccine in comparison with pegylated IFN (Peg-IFN) in patients with CHB.

## Methods

### Study design

This phase III, treatment controlled, open label and randomized clinical trial was conducted at Bangabandhu Sheikh Mujib Medical University, and Farabi Hospital, Dhaka, Bangladesh in compliance with the Declaration of Helsinki and with the principles of Good Clinical Practice. The study was approved by Institutional Review Board (IRB) of both hospitals. Directorate General of Drug Administration of Bangladesh also provided the permission for clinical trial. The study has been registered in ClinicalTrials.gov (NCT01374308). All patients gave written informed consent for the study.

### Study population

All patients enrolled in this study were diagnosed as CHB on the basis of serological, biochemical, virological, and imaging assessments. All of them were treatment naïve and none has received any antiviral or immune stimulatory drug for HBV infection. Patients of both genders were enrolled, with ages from 18–65 years. All of them carried HBsAg and HBV DNA in blood for more than 6 months. The levels of alanine aminotransferase (ALT) at enrolment had to be above the upper limits of normal (ULN) values, HBV levels were more than 10^3^ copies/mL for hepatitis B e antigen [HBeAg(-)] patients and 10^4^ for HBeAg(+) patients.

**Fig 1:**
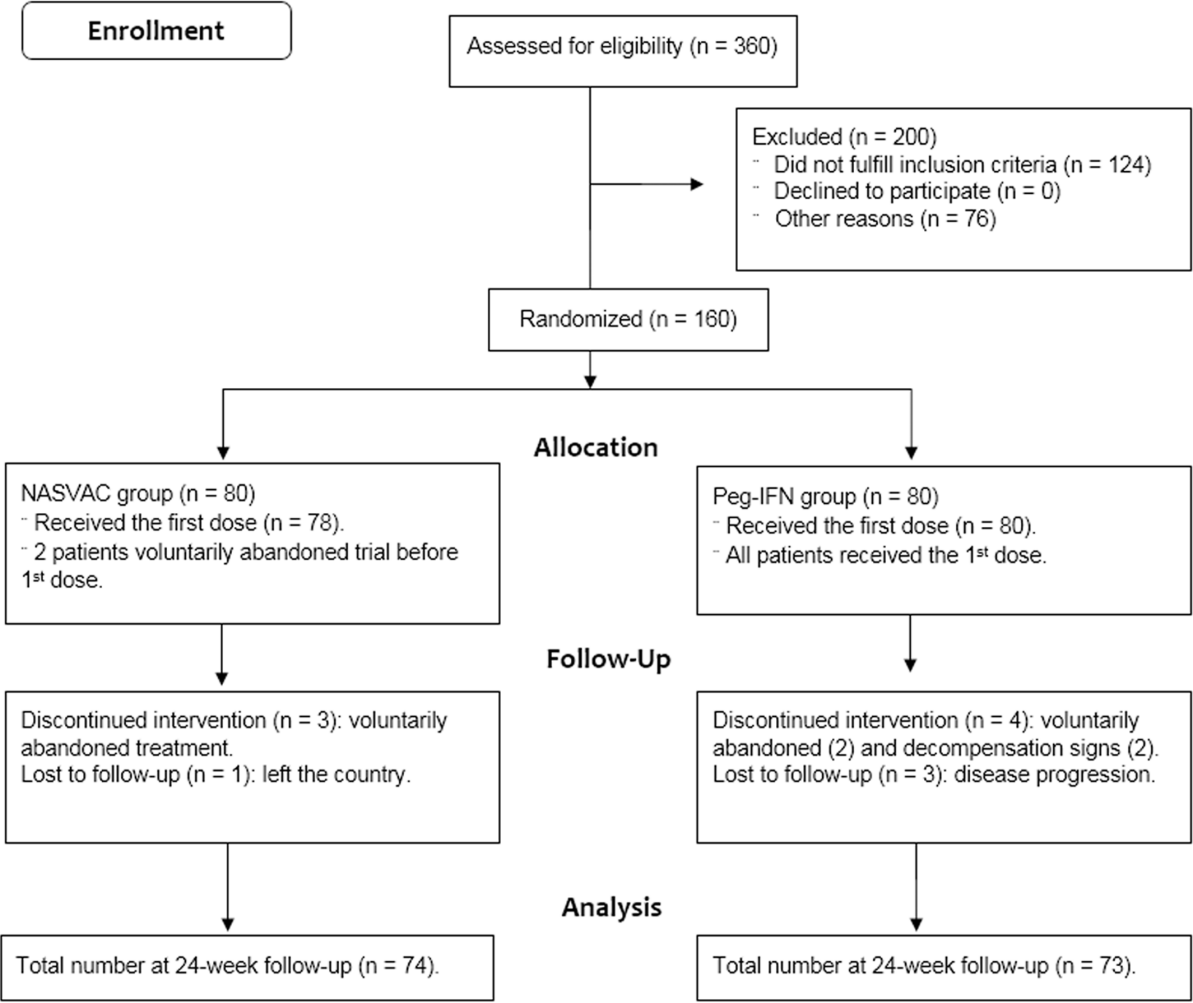
CONSORT 2010 flow diagram. Flow diagram following CONSORT guidelines, comprising the steps of patient enrollment, allocation and follow-up.

Patients were excluded from the study in case of either immune tolerant or inactive carrier state with normal ALT or advanced liver disease with cirrhosis and/or HCC; positive serology for hepatitis C, hepatitis delta or human immune-deficiency virus; previous treatment for CHB; critically ill patient; hypertension; hyperthyroidism; epilepsy; malignancies or any non-controlled systemic disease; pregnancy or nursing women; women in fertile ages without any contraceptive methods; known severe allergic conditions or hypersensitivity, severe psychiatric dysfunction or another limitation that prevents the patient’s consent; autoimmune diseases or treatment with immune suppressive or immune modulator drugs during or in the 6 months previous to the study; history of alcohol or drug abuse within one year before entry; presence of other hepatic diseases of different etiology and very high levels of ALT at the beginning of treatment (ALT above 500 U/L) suggesting unstable disease or acute flares over 10 times the upper limit of normal (ULN). Out of a total of 360 CHB patients screened, 160 were finally selected for the study after fulfilling inclusions and exclusions criteria (Fig 1).

### Study interventions

**Fig 2:**
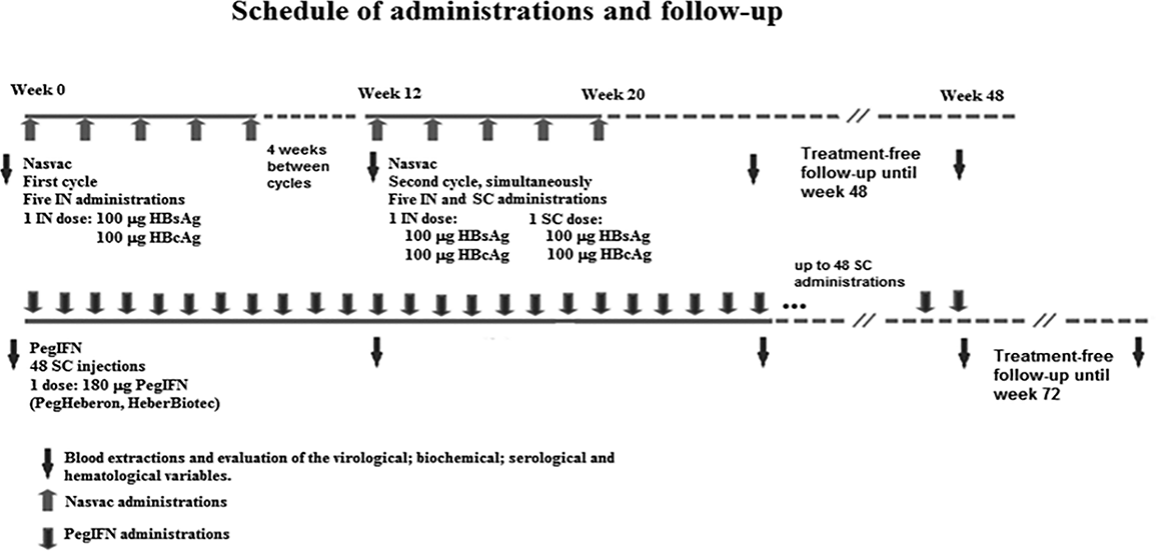
Schedule of administrations and follow-up. Time schedule of administrations, blood extractions, frequency and number of administrations as well as the administration route and composition of treatment and control groups (Nasvac and PegIFN).

The patients were randomly divided into two groups (1:1). Eighty patients were assigned to receive 180 μg of Peg-IFN (PegHeberon, Peg-IFN alpha 2b, Center for Genetic Engineering and Biotechnology, [CIGB], Havana, Cuba) once weekly for 48 consecutive weeks. The other 80 patients received NASVAC. This formulation comprises equal amounts of 100μg of HBsAg (Pichia pastoris-derived recombinant HBsAg subtype adw2) and 100μg of HBcAg (purified Escherichia coli-expressed recombinant full-length HBcAg, GenBank accession no. X02763). NASVAC was designed, produced, and developed by the CIGB, Havana, Cuba and has satisfactorily passed pharmacological, stability, toxicological tests as well as clinical trials in healthy and CHB patients [14–18]. Therapeutic vaccination was conducted in two cycles, as described in the present Phase-IIIclinical trial schedule of administration and follow-up (**Fig 2**). In the first cycle, NASVAC was administered in a volume of 1.0 ml via the intranasal route using a nasal spray on five occasions at 2-weekly intervals. The second cycle started at week 12, the same vaccine formulation was administered simultaneously via the nasal (1.0 ml containing 100 μg of HBsAg and 100 μg of HBcAg) and subcutaneous routes (1.0 ml containing 100 μg of HBsAg and 100 μg of HBcAg) on five occasions at 2-weekly intervals. All patients were observed for 2 h after each vaccination and also periodically after vaccination. Serum was collected from each patient before the study started, before each vaccination, after 5 nasal vaccinations (end of first cycle) and after the end of second cycle (end of treatment [EOT]) (Fig 2). The follow-up study was conducted 24 weeks after the EOT.

The occurrence of adverse events to the treatment was most carefully assessed in this clinical trial. Adverse reactions were measured immediately and up to 2 hours after immunization. Any adverse events during the inter-immunization periods were also recorded before the administration of the next dose of the products. In addition to the collection of adverse events, blood from all patients was tested to assess hematology and the general biochemical parameters for liver and kidney function. Sera were also collected at EOT, and 24 weeks after EOT to assess the long-term safety of NASVAC.

The levels of HBV DNA, ALT, HBeAg, Anti-HBe, and levels of fibrosis were evaluated before treatment and at different points during and after treatment. All assessments were made at tertiary level reference laboratories of Dhaka, Bangladesh by standard methodology under good clinical practices as assessed by monitoring and regulatory audits.

### Randomization

A total of 160 patients were randomly assigned to treatment NASVAC or Peg-IFN with a randomization 1:1. A computer generated list based on the software 2N, created in the University of Arkansas by Prof. Martin Hauer-Jensen. Patients were assigned according to their arrival order. Type of randomization: by blocks, with a block size of 20.

### Study outcomes

The viral load reduction was taken as its primary outcome. The success criterion was in relation with the proportion of patients showing reduction of the viral load under the limit of detection (250 copies/mL) after 24 weeks of each treatment completion, corresponding to weeks 48 and 72 for NASVAC and Peg-IFN, respectively. The virological response was measured by the HBV serum DNA quantification of the patients by real time PCR systems. The viral load quantification was performed before immunization and at weeks 12, 24, 48 and 72. Validated procedures and equipment were used, specifically the ABI Prism 7300 SDS Real-Time PCR system (USA) and the quantification kit Genebio HBV (Italy) and the RoboGene HBV DNA (Germany) Quantification Kits.

The secondary outcomes were: biochemical response as measured by the serum ALT transaminase level, according to the procedures established in the Farabi Hospital, Dhaka and evaluated every 12 weeks; serological response as measured by HBsAg detection and their specific antibodies (weeks 0, end of treatment and end of follow-up); serum HBeAg detection and its conversion to anti-HBeAg antibodies (weeks 0, end of treatment and end of follow-up); histological response as measured by Fibroscan (week 0 and week 96).

Regarding safety measurements, a careful registration of the adverse events was carried out by specialized and dedicated team of researchers as a secondary but very relevant outcome every administration date. As the control treatment (Peg-IFN) required a specialized follow up, the vaccinated patients were followed in deep to explore any AE during the treatment and follow-up. The variable related to the treatment´s safety were: Type of adverse event, intensity of the adverse event, duration of the adverse event, evolution of patients’ symptoms and causality relationship.

The control variables were: age, sex, weight, body mass index, disease duration previous to the start of the treatment, viral load and Initial characteristics of the disease, toxic habits and the background of reaction to other treatment.

### Sample size and statistical analysis

The sample size calculation was carried out under the hypothesis that the proportion of patients with CHB able to reduce the viral load (VL) to undetectable levels (250 copies/mL) at the follow up determination is higher than 20% in the group immunized with NASVAC as compared to the group treated with Peg-IFN, H0:δ≤δ_0_ = 0.20 *vs*. HA:δ>δ_0_ = 0.20.

To accomplish this hypothesis with a pre-fixed precision of 0.05, 80% potency and a loss percentage that may cover abandons for any cause of 10%, 80 patients will be needed for group 1 (immunized with NASVAC) and 80 patients for group 2 (inoculated with Peg-IFN), therefore the total sample size will be 160 patients.

Nominal and categorical parameters were expressed as absolute numbers and percentages. Mean, standard deviation, median, interquartile range and/or ranges were calculated for all quantitative variables.

Characteristics at baseline (gender, age, height, weight, BMI, HBV DNA level, ALT and HBeAg level), occurrence of adverse events during treatment and sustained virological response at the end of treatment were compared between both groups of patients using a Pearson’s Chi-square test or a Fisher's exact test for categorical parameters and a t-test or a non-parametric Mann–Whitney test for continuous parameters.

Mean HBV DNA level at baseline and at different time points during treatment (W12, EOT, W24(FU)) were compared between both groups of patients using a non-parametric Mann–Whitney test. Moreover, HBV DNA level decrease between baseline and different time points during treatment (W12, EOT, W24(FU)) within each group was assessed using a non-parametric Wilcoxon test. For all analyses, a two-tailed significance testing and a significance level of 0.05 were used. Statistical analysis was performed using SPSS version 15.0 for Windows (SPSS Inc., Chicago, IL, USA).

## Results

### Study population

A total of 360 patients were assessed for eligibility, and 160 were finally selected and randomized into two groups of 80 patients (1:1). Only 2 patients voluntarily dropped out of the trial before start of NASVAC treatment, but none due to adverse reaction during treatment 4 out of 80 patients in the Peg-IFN group withdrew from Peg-IFN treatment; in 2 cases because of signs of cirrhotic decompensation and in 2 others for non-treatment related causes.

### Recruitment and treatment

Patients were enrolled in the trial and treated with NASVAC from June, 7th 2011 upto January 3rd 2012. In the case of Peg-IFN treated patients, the enrollment started the same day and was completed on May 5th, 2012.

### Baseline data

The enrolled patients were young with a mean age of 29±7 years in both groups. General parameters like height, weight, and BMI were comparable between two groups. All patients were phenotypically from Indian ethnical background. Only 15 patients (19.2%) in NASVAC group and 18 patients (22.5%) in the Peg-IFN group were HBeAg positive. This reflects the natural prevalence pattern of CHB patients in Bangladesh and also in Indian subcontinent that show a higher prevalence of HBeAg (-) CHB patients. No statistical differences were found in baseline levels of HBV DNA, ALT or any of the demographic or baseline variables between two groups (**Table 1**).

**Table 1:** The patients’ baseline data.

Variables		NASVAC		Peg-IFN	
		N	%	N	%
Total		78	49.4	80	50.0
Sex	Female	14	17.9	10	12.5
	Male	64	82.1	70	87.5
Age (years)	Median ± IQR (range)	28 ± 10 (18–50)		28 ± 11 (18–48)	
Height (m)	Median ± IQR (range)	1.52 ± 0.00 (1.22–1.83)		1.52 ± 0.00 (1.22–1.83)	
Weight (kg)	Median ± IQR (range)	59 ± 16 (36–81)		61 ± 15 (35–85)	
Body mass index (kg/m^2^)	Median ± IQR (range)	25.78 ± 6.83 (15.50–36.85)		26.36 ± 6.43 (17.22–40.26)	
HBV DNA (log copies/mL)	Median ± IQR (range)	4.7 ± 1.8 (3.2–13.0)		5.2 ± 2.6 (3.1–12.5)	
ALT (IU/L)	Median ± IQR (range)	30.0 ± 22.0 (10.0–262.0)		37.0 ± 19.8 (10.0–226.0)	
HBeAg(+) at baseline	N (%)	15 (19.2)		18 (22.5)	

The absence of significant differences between groups in demographic as well as the most important clinical variables support the randomization quality.

SD; Standard Deviation

IQR; Interquartile range

IU; International unit.

### Numbers analyzed

The statistical hypothesis was tested 24 weeks after the end of each treatment, when less than 10% of patients dropped out. A total of 74 and 73 patients were analyzed in NASVAC and Peg-IFN treatment groups, respectively (**Fig 1**).

### Outcomes and estimation

At EOT, both NASVAC and Peg-IFN therapy induced similar proportion of HBV DNA reduction. HBV DNA<250 copies/ml were detected in almost similar percentages of patients in NASVAC group versus Peg-IFN group (59.0% versus 62.5%, p>0.05). Also, patients receiving NASVAC and Peg-IFN showed almost similar proportion of patients with HBV DNA< 1000 copies/ml at EOT (69.2% versus 65%, NASVAC versus Peg-IFN, p>0.05).

The statistical hypothesis of the present clinical trial was verified during treatment free follow up 24 weeks after EOT. A significantly higher (p<0.01) proportion of patients with viral load below 250 copies/ml was indeed found in NASVAC recipients (57.7%) as compared to the Peg-IFN treated group (35.0%). Also, the levels of HBV DNA<1000 copies were detected in 71.8% and 45% patients receiving NASVAC and Peg-IFN, respectively. Taken together, Peg-IFN treated group evidenced a more pronounced post-treatment (EOT) viral rebound, with a significant reduction in the proportion of patients with controlled viral loads.

In addition to the ratio of patients controlling HBV DNA, the mean levels of HBV DNA in the sera of these patients were also compared at baseline and different times after therapy (**Table 2**). At baseline, the levels of HBV DNA were comparable between NASVAC recipients and Peg-IFN treated CHB patients (5.4 ± 2.1 versus 5.8 ± 2.3 log copies/ml). At EOT, the levels of HBV DNA were reduced in both groups, and the levels of reduction were also similar without statistical difference (Table 3). Conversely, 24 weeks after EOT, the patients receiving NASVAC maintained HBV DNA suppression almost at EOT level. However, the patients receiving Peg-IFN experienced rebound of HBV DNA from 3.0 ± 1.5 log copies/ml to 4.3 ± 2.2 log copies/ml (p<0.001) (**Table 2**).

**Table 2:** Kinetics of HBV DNA in CHB patients receiving NASVAC or Peg-IFN.

	Treatment	NASVAC	Peg-IFN
Baseline	Mean ± SD^a^	5.4 ± 2.1	5.8 ± 2.3
	Median	4.7	5.2
	(Min; Max)	(3.2; 13.0)	(3.1; 12.5)
	*Mann Whitney (p)*	*0*.*471*	
Week 12	Mean ± SD	3.1 ± 1.3	3.1 ± 1.5
	Median	2.4	2.4
	(Min; Max)	*(1*.*1; 7*.*7)*	*(0*.*9; 8*.*2)*
	*Mann Whitney (p)*	*0*.*573*	
Baseline vs Week 12	*Wilcoxon (p)*	*<0*.001(***)	*<0*.001(***)
EOT^b^	Mean ± SD	2.8 ± 1.1	3.0 ± 1.5
	Median	2.4	2.4
	(Min; Max)	*(0*.*8; 6*.*6)*	*(1*.*9; 9*.*6)*
	*Mann Whitney (p)*	*0*.*851*	
Baseline vs EOT	*Wilcoxon (p)*	*<0*.001(***)	*<0*.001(***)
Week 24 follow-up (FU)	Mean ± SD	2.9 ± 1.3	4.3 ± 2.2
	Median	2.4	3.1
	(Min; Max)	*(0*.*8; 7*.*5)*	*(2*.*1; 9*.*3)*
	*Mann Whitney (p)*	*<0*.001(***)	
EOT vs Week 24 (FU)	*Wilcoxon (p)*	*0*.*660*	*<0*.001(***)

Mean±SD and median values of HBV DNA at baseline, week 12, end of treatment (EOT), and 24 weeks after EOT (Week 24 follow-up (FU)) are shown. Mann Whitney’s *p* value compared the statistical significance between NASVAC-group versus Peg-IFN group. Data revealed that the levels of HBV DNA were significantly lower in NASVAC-recipients compared to Peg-IFN at week 24 Follow-up. Wilcoxon’ p value compared HBV DNA levels of NASVAC or Peg-IFN at different time points. HBV DNA levels did not increase 24 weeks after EOT compared to levels at the EOT in NASVAC-recipients, on the other hand, in Peg-IFN recipients HBV DNA increased significantly at the 24 weeks after EOT compared to levels at the EOT.

^a^SD, Standard deviation

^b^EOT, end of treatment

*p*<0.05 indicates statistical significance.

**Table 3:** Adverse events detected in patients receiving NASVAC and Peg-IFN.

Adverse Event	Stats	NASVAC (N = 78)		Peg-IFN (N = 80)		Total (N = 158)	
		N	%	N	%	N	%
At least one episode of AE^a^	*p < 0*.*001*	61	78.2	80	100.0	141	89.2
Fever	*p < 0*.*001*	34	55,7	78	97,5	112	79,4
Weakness	*p < 0*.*001*	20	32,8	67	83,8	87	61,7
General malaise	*p < 0*.*001*	12	19,7	65	81,3	77	54,6
Headache	*p < 0*.*050*	15	24,6	35	43,8	50	35,5
Local pain	*p = 0*.*438*	14	23,0	23	28,8	37	26,2
Nausea		8	13,1	18	22,5	26	18,4
Lose motion		4	6,6	18	22,5	22	15,6
Gastrointestinal disorder		4	6,6	8	10,0	12	8,5
Hair fall		1	1,6	10	12,5	11	7,8
Vomiting		4	6,6	6	7,5	10	7,1
Anxiety		1	1,6	8	10,0	9	6,4
Vertigo		4	6,6	4	5,0	8	5,7
Fatigue		0	0,0	8	10,0	8	5,7
Body allergy		3	4,9	4	5,0	7	5,0
Skin rash		1	1,6	6	7,5	7	5,0
Dyspepsia		0	0,0	7	8,8	7	5,0
Sneezing		2	3,3	4	5,0	6	4,3
Bitter taste		1	1,6	5	6,3	6	4,3
Aphthous ulcer		0	0,0	6	7,5	6	4,3
Itching		1	1,6	4	5,0	5	3,5
Gum bleeding		0	0,0	5	6,3	5	3,5
Slight fever		4	6.6	0	0,0	4	2,8

The treatment with NASVAC was safer to PegIFN considering the most relevant AE detected during the study.

^a^AE, Adverse Event

### Elevation of ALT by nasal administration of NASVAC in CHB patients

**Fig 3:**
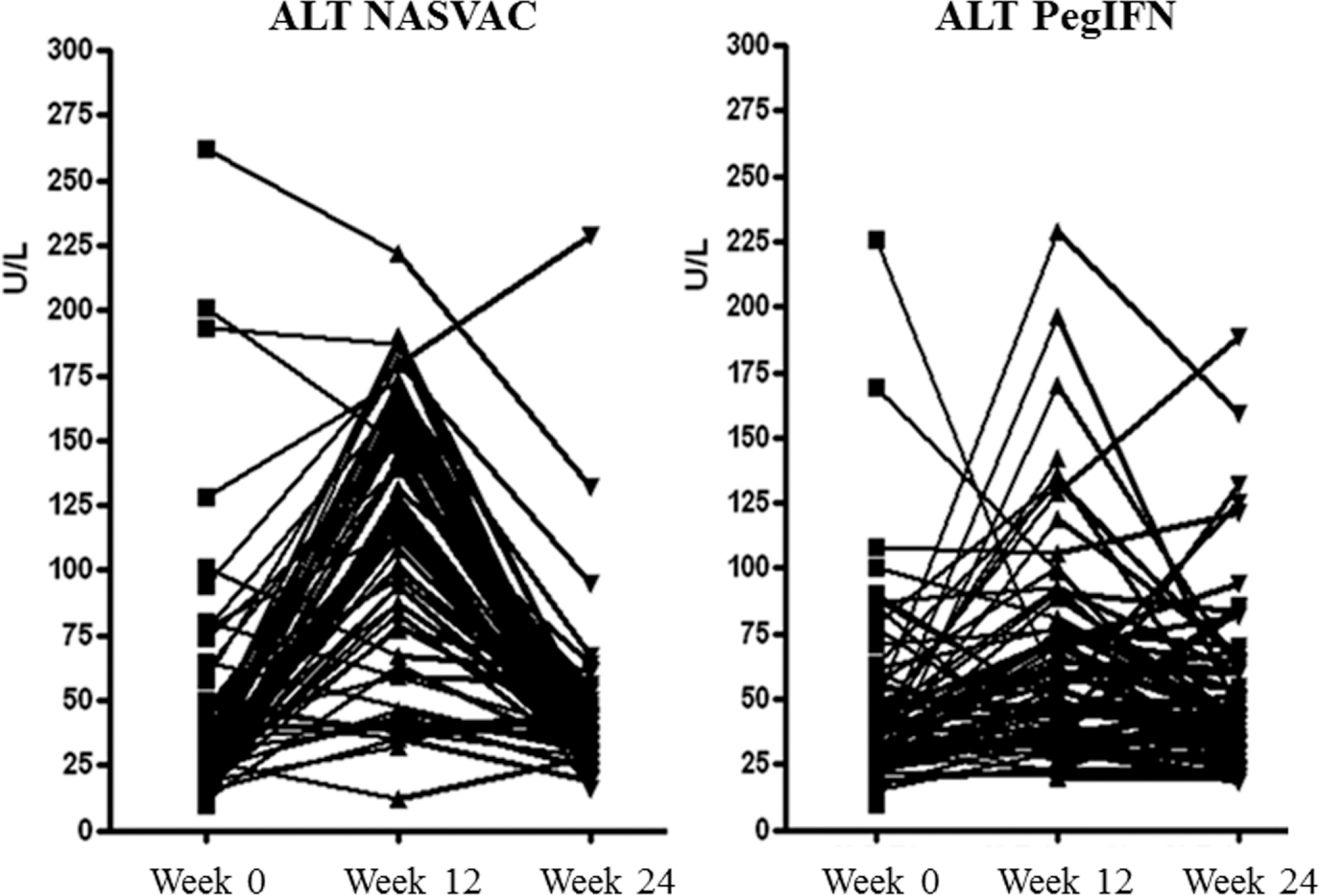
Changes in serum ALT levels during therapeutic vaccination. The study of serum ALT levels evidenced a transient, homogenous and significant increase of transaminase values in vaccinated compared to PegIFN treated patients at week 12.

ALT increase (>2X ULN) was recorded in 85% of NASVAC-treated patients after 5 nasal vaccinations. These flares occurred independently of patients HBe serology, sex, age or initial viral load. The nature of the ALT increases in NASVAC group was transient, homogeneously related to week 12 and reached five to ten times baseline levels. The ALT flares in some Peg-IFN treated patients had a similar range of intensity (up to 300 U/L) but such increases occurred in only 30% patients. In addition, the effect was not homogeneously related to the week 12 (**Fig 3**).

### HBeAg & anti-HBeAg determinations

A total of 5 out of 14 NASVAC treated patients (35.7%) seroconverted from HBeAg to anti-HBe within 24 weeks of treatment-free follow-up while only 3 out of 16 patients seroconverted to anti HBeAg (18.7%) in the Peg-IFN group.

All HBe/anti-HBeAg seroconversions were associated to strong reduction of HBV DNA in both the NASVAC and Peg-IFN treated groups. Patients who cleared HBeAg had a significantly deeper reduction in mean viral load value compared to patients with persisting HBe antigenemia.

### Assessment of liver fibrosis

The fibroscan follow up monitoring evidenced that none of the NASVAC-recipient patients had stiffness values over 18.3 kPa at 24 week after EOT, (a level corresponding to cirrhosis on the Metavir score) while 7 patients receiving Peg-IFN treated had liver stiffness of ≥ 18.3 kPa cut off at 24 week after EOT.

### Harms

#### Safety assessment

There was no serious adverse event in any of the groups. The NASVAC treatment was not stopped due to any severe event. Severe adverse events were mainly reported during Peg-IFN treatment. These adverse events have been listed in **Table 3.**

## Discussion

Among drugs which are used for treatment of CHB, Peg-IFN is endowed with both antiviral and immunomodulatory activities. In addition, Peg-IFN can be used for a finite course. Based on these premises, a phase III, open label randomized controlled clinical trial was designed to directly compare the therapeutic efficacy and tolerance of a therapeutic vaccine candidate, NASVAC versus Peg-IFN in treatment-naïve CHB patients.

NASVAC was significantly safer compared to Peg-IFN in all major variables related to adverse events. Furthermore, the development of signs of hepatic decompensation was only found in 2 patients receiving Peg-IFN, but in none of the NASVAC treated group (Table 2). It should be emphasized that this phase III clinical trial is a continuation of the Phase I/II clinical trial which had been completed 7 years earlier. The immunization schedule of phase I/II study and of this phase III is completely similar regarding dose, duration and route of administration of NASVAC. The patients of Phase I/II clinical trial did not develop any notable adverse event related to NASVAC therapy over the last 7 years. Taken together, the overall data indicate that NASVAC is a safe immune therapeutic agent for CHB patients in both short-term and long-term perspectives.

The efficacy of NASVAC should be appreciated in the context of various frustrating immune therapy attempts for CHB treatment [19]. These include: HBsAg-based vaccines [20], vaccines in combination with nucleoside analogs [11, 21], vaccine combined with potent adjuvants [11], HBV DNA-based vaccine [21, 22], HBsAg/anti-HBs complex vaccine [23] and other therapeutic vaccines [19]. All have been found to be safe for CHB patients, but, their efficacy has not been satisfactory [19]. At present, it is considered that although vaccine therapy may represent an attractive concept, evidence-based approaches and proper design remain to be determined to reach desirable outcomes.

NASVAC was developed to this end, and its safety and efficacy were checked in HBV transgenic mice, normal volunteers and patients with CHB in a phase I and I/II clinical trials. Key pivotal aspects of this study deserving to be stressed are the following: NASVAC therapeutic vaccine combines both HBsAg and HBcAg. Evidence from early 2000 did show that HBcAg-specific immunity is essential to control HBV replication and liver damage [13]. Remarkably, studies in HBV transgenic mice revealed that HBcAg acts like an adjuvant to induce and sustain HBV-specific immunity which is safe and exhibits antiviral activity [16]. The successive regimens of vaccine therapy for CHB have assayed the usual doses and schedules of vaccine derived from experiences of prophylactic vaccination [19, 20]. In this study, we used 200 micrograms of antigens per shot in the first cycle and 400 micrograms of antigens were used in the second cycle in a 1:1 proportion.

Finally, NASVAC has been administered by both nasal and parental routes allowing stimulation of both mucosal and systemic immune compartments of CHB patients. The elevation of ALT found in the majority of CHB patients treated with NASVAC after only 5 nasal vaccinations was not detected in healthy volunteers after NASVAC administration [17]. Thus, NASVAC triggered increases of ALT, an indirect marker of restoration of host immunity, after nasal administration. This may be a key factor related to the outstanding therapeutic effect of NASVAC in CHB patients. Indeed, reduction of HBV DNA levels did follow ALT elevations in most NASVAC recipients. This finding mimics the natural history of immune restoration in CHB patients evolving from an immune tolerance state to an immune clearance phase [24]. Conversely, many patients receiving Peg-IFN often showed HBV DNA reduction but without elevation of ALT, possibly suggesting a more direct antiviral effect of Peg-IFN [25].

In conclusion, we present here a phase III study which used a therapeutic vaccine comprising both HBsAg and HBcAg, at high doses (up to 100–200 μg of each antigen), administered 10 times by the nasal and five by subcutaneous routes, in treatment naïve patients with CHB. This regimen was well tolerated and safer than Peg-IFN. Serum HBV DNA clearance was found in a significantly higher proportion of patients receiving NASVAC as compared to Peg-IFN recipients 24 weeks after EOT. This was further confirmed by significantly lower levels of HBV DNA in NASVAC-treated patients compared to Peg-IFN treated patients. The progression of hepatic fibrosis was also better controlled in NASVAC compared to Peg-IFN recipients, even though significant increases of ALT were recorded in almost all patients receiving NASVAC.

Finally, the therapeutic benefit of NASVAC was obtained after 15 nasal/subcutaneous immunizations within only 6 months as compared to 48 doses of Peg-IFN over a year. This much shorter treatment duration reduces the potential adverse effects and increases the treatment adherence. The strong potential to further improve efficacy of NASVAC by optimizing the schedule of immunization warrant further study. Similarly, the impacts of NASVAC on nucleoside treated patients remains to be evaluated.

This study indicates that the impact of therapeutic vaccination in the treatment of chronic infectious diseases will depend on the capacity of designing the adequate antigens and adjuvant strategies as well as the selection of the most suitable immunization route(s) and candidate recipients. The present clinical results underline the need for further investigations to assess the impact of mucosal immunization and its mechanisms of action in the field of therapeutic vaccination.

## Supporting information

S1 Checklist CONSORT checklist. (PDF)

S1 File Minimal anonymized data set: Introduction. The file includes the following information: Statement on informed consent for data publication; Information regarding the variables used in the study, statistical hypothesis and justification of the sample size; and the Nature of the files used to provide the data in the *Anonymized data set*.
(DOCX)

S2 File Trial study protocol. (PDF)

S1 Table ANNEX 1 Virology HBs HBeAg Serology.doc. The file contains the results of Virological and serological evaluations of the timepoint assessments included in the publication.
(DOC)

S2 Table ANNEX 2 Biochemistry and liver function tests.doc. The file contains the results of Biochemistry and liver function tests of the timepoint assessments included in the publication.
(DOC)

S3 Table ANNEX 3 Adverse Events.doc to your manuscript. The file contains the results of adverse events detected during the study.
(DOC)
