# Supplementary material for: Treatment of chronic hepatitis B naïve patients with a therapeutic vaccine containing HBs and HBc antigens (a randomized, open and treatment controlled phase III clinical trial)
Source: PLoS One. 2018 Aug 22;13(8):e0201236. doi: 10.1371/journal.pone.0201236 (PMC6104936; doi:10.1371/journal.pone.0201236)
Supplement: S1 File — The file includes the following information: Statement on informed consent for data publication; Information regarding the variables used in the study, statistical hypothesis and justification of the sample size; and the Nature of the files used to provide the data in the Anonymized data set. (DOCX) [file pone.0201236.s002.docx]

**S1 File.** Minimal anonymized data set

The file includes the following information: Statement on informed consent for data publication; Information regarding the variables used in the study, statistical hypothesis and justification of the sample size; and the Nature of the files used to provide the data in the *Anonymized data set*.

1. **Statement on Informed consent for data publication:**

Consent for publication of raw data obtained from study participants

1. **Regarding publication in journals**

It has been considered that the raw data can be published in journal.

## 3. Regarding the variables used in the study

All study variables are well known for a reviewer skilled in the art.

Quantitative viral load (HBV DNA) was the main variable of the study. The statistical hypothesis is based in a superiority design trial and defines the sample size of the study (3.1). In addition the other variables are the variables are usually assessed to understand the effect of any treatment on main viral antigens (serological variables HBsAg and HBeAg/Ab), the blood biochemical markers of liver damage (ALT and AST) as well as the liver function tests most frequently used in literature (Bilirubin and Alkaline phosphatase). In addition to virological, serological and biochemical variables, and liver function tests in Annexes 1 and 2, we are providing in Annex 3 the detailed database of all detected adverse events collected during the administration of both products in their frequency and related time schedule.

## 3.1 Statistical hypothesis and Justification of the sample size.

Sample size calculation is carried out under the hypothesis that the proportion of patients with chronic Hepatitis B reducing their viral charge to undetectable levels (defined as values under 250 copies/mL) is greater than 20% in the group immunized with NASVAC with respect to the group immunized with IFN-PEG, H_0_:δ≤δ_0_=0.20 vs. H_A_:δ>δ_0_=0.20.

To carry out this hypothesis with a precision of 0.05, a potency of 80% and a percentage loss covering 10% of abandons by any cause, the result is 80 patients are needed for group 1 (group immunized with NASVAC), 80 patients for the group 2 (immunized with IFN-PEG), reaching a sample size of 160 patients in total.

**Sample Sample Prop|H1 Prop**

Size Size Grp 1 or Grp 2 or Diff Diff

Grp 1 Grp 2 Trtmnt Control if H0 if H1 Target

Power N1 N2 P1 P2 D0 D1 Alpha

- 1. 72 72 0.5000 0.3000 0.0000 0.2000 0.0500

**4. Nature of the files used to provide the data in the *Anonymized data set*.**

Considering the presence of qualitative as well as quantitative variables, all data has been provided in word format and inserted in tables than can be easily transformed into excel or any other file formats needed for statistical analysis, similarly the number format is given in the case of HBV DNA in the scientific notation as required by several statistical systems.
