# Supplementary material for: Treatment of chronic hepatitis B naïve patients with a therapeutic vaccine containing HBs and HBc antigens (a randomized, open and treatment controlled phase III clinical trial)
Source: PLoS One. 2018 Aug 22;13(8):e0201236. doi: 10.1371/journal.pone.0201236 (PMC6104936; doi:10.1371/journal.pone.0201236)
Supplement: S2 File — (PDF) [file pone.0201236.s003.pdf]

# CLINICAL TRIAL PROTOCOL

**“Phase II-III study of a therapeutic vaccine candidate containing the hepatitis B virus (HBV) core antigen (HBcAg) and the HBV surface antigen (HBsAg) for chronic HBV infection (CHB) treatment”**

**Version 1: September**

**2009**

**Table of Contents**

1 General Information ........ 1

1.1 TITLE ........ 1
1.2 SPONSORS, INVESTIGATORS AND MONITORS ........ 1
1.2.1 SPONSOR ........ 1

*1.2.2 Principal investigator ........ 1*

*1.2.3 Other Investigators ........ 1*

*1.2.4 Monitors* ........ 2
1.3 INSTITUTION WHERE THE TRIAL WILL BE CARRIED OUT ........ 2
1.4 DATA MANAGEMENT AND STATISTICAL ANALYSIS ........ 2

1.5 RESPONSIBLE ENTITIES FOR CONSERVATION, LABELING AND SUPPLY OF MEDICATIONS ........ 2

2 Justification and Objectives ........ 3

2.1 PURPOSE OF THE TRIAL ........ 3
2.2 GENERAL OBJECTIVE ........ 3
2.3 SPECIFIC OBJECTIVES ........ 3
2.4 REASON FOR WHICH THE TRIAL IS CARRIED OUT AND BACKGROUND INFORMATION ABOUT THE VACCINES ........ 4
2.5 FUNDAMENTAL BACKGROUND ........ 6

3. Ethics and Medical Deontology ........ 20

3.1 GENERAL ETHICAL CONSIDERATIONS ........ 20
3.2 TRIAL AUTHORIZATION AND APPROVAL ........ 21
3.3 EXPLANATION ON HOW THE PATIENTS WILL BE INFORMED AND THEIR CONSENT WILL BE OBTAINED ........ 21

4. General design ........ 22

4.1 TYPE OF TRIAL: OPEN PHASE II CLINICAL TRIAL. ........ 22
4.2 DESIGN OF THE TRIAL ........ 22

5. Selection of subjects ........ 23

5.1 POPULATION ........ 23
5.2 DIAGNOSTIC CRITERIA ........ 23
5.3 INCLUSION CRITERIA ........ 24
5.4 EXCLUSION CRITERIA ........ 24
5.5 EXIT CRITERIA OR INTERRUPTION OF THE TREATMENT AFTER INCLUSION ........ 25

6. Treatment ........ 25

6.1 PRODUCTS TO BE USED ........ 25
6.2 RULES FOR THE USE OF CONCOMITANT TREATMENTS ........ 26
6.3 MEASURES TO GUARANTEE SAFETY IN THE MANIPULATION OF THE PRODUCTS ........ 26
6.4 MEASURES TO PROMOTE AND CONTROL THAT THE INSTRUCTIONS AND PRESCRIBED RULES ARE FOLLOWED ........ 27

7. Evaluation ........ 27

7.1 EVALUATION CRITERIA REGARDING IMMUNOGENICITY / EFFICACY ........ 27

*Main variable ........ 27*

*Secondary variables ........ 27*

*Control variables* ........ *27*
7.2 VARIABLES USED TO MEASURE THE EFFECTS AND PERIODICITY OF THE DETERMINATIONS ........ 28
*Evaluation criteria regarding safety* ........ *28*
7.3 EXPLANATION OF HOW TO ACT IN CASE OF ABANDONMENT OR EXIT IN THE TRIAL ........ 28
8. Adverse reactions ........ 30
8.1 ADVERSE EVENTS THAT CAN BE PRESENTED AND METHODS TO REGISTER THEM ........ 30
8.2 PROCEDURE FOR ADVERSE REACTIONS TREATMENT ........ 30
8.3 NOTIFICATION OF ADVERSE EVENTS (AE) ........ 30
9. Data management ........ 31
9.1 PROCEDURES ........ 31
10. Statistics ........ 32
10.1 STATISTICAL TESTS ........ 32
*Evaluation of the viral infection markers and statistic hypothesis* ........ *34*
*Evaluation of the biochemical status* ........ *35*
*Adverse events (AE)* ........ *35*
*Data center:* ........ *35*
*Number of patients* ........ *36*
*Interim analysis* ........ *36*
10.3 GENERAL SCHEDULE ........ 36
10.4 INTERIM ANALYSIS ........ 36
10.5 FINAL ANALYSIS ........ 36
11. Practicalities ........ 37
11.1 DETAILED PLAN. SUBJECT MANAGEMENT ........ 37
11.2 DISTRIBUTION OF DUTIES AND RESPONSIBILITIES AMONG THE PARTICIPANTS IN THE INVESTIGATION ........ 37
*Duties of the sponsor (CIGB)* ........ *37*
*Duties of the monitors* ........ *38*
*Duties of the Principal investigators* ........ *38*
*Duties of rest of the investigators* ........ *39*
11.3 INSTRUCTIONS TO THE PERSONNEL ........ 40
11.4 ADDRESSES AND TELEPHONE NUMBERS OF THE MAIN INVESTIGATOR ........ 40
11.5 CONSIDERATIONS ABOUT CONFIDENTIALITY AND DISCLOSURE OF THE RESULTS ........ 40
11.6 QUALITY CONTROL AND AUDITS ........ 40
12. Assurance ........ 41
12.1 NECESSARY RESOURCES ........ 41
13. References ........ 42

# 1 General Information

**Title**

"Phase II-III study of a therapeutic vaccine candidate containing the hepatitis B virus (HBV) core antigen (HBcAg) and the HBV surface antigen (HBsAg) for treatment of patients with chronic hepatitis B"

## 1.2 Sponsors, Investigators and monitors

### 1.2.1 Sponsor

Center for Genetic Engineering and Biotechnology (CIGB), Havana / Heber Biotech, Havana.

### 1.2.2 Principal investigator

Dr. Mamun-Al-Mahtab MBBS, MSc (Gastroenterology, London), MD (Hepatology), FACG

Assistant Professor, Department of Hepatology Bangabandhu Sheikh Mujib Medical University Consultant Hepatologist, Gastroenterologist & Interventional Endoscopist, Lab Aid Specialized Hospital Dhaka, Bangladesh

Secretary General, Viral Hepatitis Foundation and Bangladeshi Association for the Study of the Liver.

Senior Editor, Bangladesh Liver Journal Senior; Editor, Hungarian Medical Journal

Correspondence:

House No. 109, Road No. 4
Block. B, Banani
Dhaka-1213, Bangladesh
Tel: +880-2-8850371
Mobile: +880-1711567275
Fax: +880-2-8826840
E. mail: shwapnil@agni.com
Alternate Email <hbd@dhaka.net>

### 1.2.3 Other Investigators

*Principal Investigator Ehime University Graduate School of Medicine, Ehime, Japan*

*Principal Investigator, Toshiba General Hospital, Tokyo, Japan*

Dr. Sheikh Mohammad Fazle Akbar

*Viral Hepatitis Foundation, Dhaka, Bangladesh*

*Labaid Dhaka Hospital, Dhaka, Bangladesh*

Professor Salimur Rahman, President, Bangladeshi Association for the Study of the Liver.

Dr. Md. Fazal Karim

UNIVERSITY STAFF

### 1.2.4 Monitors

Dr. Julio César Aguilar
Center for Genetic Engineering and Biotechnology, Havana, Cuba

Prof. Eduardo Pentón Arias
Center for Genetic Engineering and Biotechnology, Havana, Cuba

## 1.3 Institution where the trial will be carried out

Under the direct supervision of *Viral Hepatitis Foundation*, Dhaka, Bangladesh, at Bangabandhu Sheikh Mujb Medical University Hospital, Bangladesh

## 1.4 Data management and statistical analysis

Information and Statistics Group, Clinical Trials Division, CIGB, Havana.

Viral Hepatitis Foundation, Dhaka, Bangladesh

## 1.5 Responsible entities for conservation, labeling and supply of medications

Vaccines Division, CIGB, Havana, Cuba.

Viral Hepatitis Foundation, Dhaka, Bangladesh

# 2 Justification and Objectives

## 2.1 Purpose of the trial

The clinical trial has the following objectives:

## 2.2 General objective

The general objective of the present clinical trial (CT) consists in defining the immunogenicity and efficacy of the repeated NASVAC therapeutic vaccine candidate (TVC) immunization of CHB patients, under the conditions expressed in this protocol, improving and widening safety assessment according to the proposed doses and schedule.

## 2.3 Specific objectives

With the above mentioned purpose the following specific objectives are declared:

1. To evaluate the immunogenicity resulting from the application of the NASVAC TVC to CHB patients, assessing the effect of intranasal / subcutaneous administration on the induced humoral and cellular immune response and finally on the viral load as a variable dependent on immune response, that will be used as the main variable.

2. To identify possible adverse events (AE) linked to the application of the NASVAC TVC to CHB patients and assess their frequency and intensity in the scenery of intranasal/subcutaneous administration.

3. To collect and assess preliminary data on the therapeutic potential of the NASVAC TVC in CHB patients regarding to:

- ➢ Reduction of the serum HBV DNA levels.
- ➢ HBsAg / HBeAg clearance and anti-HBsAg / HBeAg seroconversion
- ➢ Reduction in the levels of transaminase (ALT) activity
- ➢ Clinical outcome of patients (as defined in the corresponding section)

***Statistical hypothesis***

It is expected that the administration of NASVAC TVC, as defined in the present protocol, to chronic hepatitis B patients, will improve the specific anti-HBV immune response of treated patients and consequently will significantly increase the percentage of patients reducing the viral load to undetectable levels after treatment completion compared to the group administered with Interferon, expecting a difference between groups of more than 20%.

H0: Equivalency in the percentage of chronic hepatitis B patients reducing the viral load to undetectable levels. Formally, H0: $\delta \leq \delta_0 = 0.20$ where $\delta$ represents the difference between viral seronegativization, based in quantitative PCR assay with a sensitivity of 250 copies/mL, in the group treated with NASVAC compared to the group treated with Interferon.

H1: Superiority in the percentage of chronic hepatitis B patients reducing the viral load to undetectable levels. Formally, H0: $\delta > \delta_0 = 0.20$ where $\delta$ represents the difference between viral seronegativization, based in quantitative PCR assay with a sensitivity of 250 copies/mL, in the group treated with NASVAC compared to the group treated with Interferon.

## 2.4 Reason for which the trial is carried out and background information about the vaccines

In general, current treatments for CHB are limited in efficacy (around 30% on the basis of some particular criteria). Previous attempts to explore the efficacy of preventive commercial vaccines used for CHB active immunotherapy have also shown a limited efficacy**.** In this sense, the use of potent adjuvants, new antigens and more immunogenic formulations have been suggested to subvert the immunotolerant status in CHB patients.

The present active immunotherapeutic strategy has been designed to address this problem. This strategy comprises the use of the major HBs and HBc HBV antigens in a novel nasal formulation named NASVAC, characterized by an important cellular and humoral immunogenicity and by the ability to activate both the systemic as well as the mucosal immune compartments in animal models. The proposed approach is considered novel since it has been proven to induce a potent immune response in animal models improving the current state of the art on CHB TVC.

Importantly, NASVAC comprises two antigens:

a) The r-HBsAg derived from the Cuban *Pichia pastoris* yeast strain transformed by genomic insertion of “s” gene to express r-HBsAg.

This antigen is molecularly identical to that derived from the *Saccharomyces cerevisiae* plasmid constructions used by most of the other vaccines. However, other features were also different for the active antigenic ingredient of the Cuban vaccine, such as a milder purification process (patented) designed to preserve HBsAg-r molecule integrity, including a unique step of preparative

immunoaffinity chromatography (on selective and specific monoclonal antibody). This process is now considered relevant for the higher immunogenicity shown by antigen in preclinical studies and clinical trials around the world. In this sense, the nasal formulation under study -NASVAC- receives the benefit of using the surface antigen with a higher immunogenicity.

b) The r-HBcAg, the 183 aa. recombinant core antigen (HBcAg) purified and obtained from *E coli* as virus-like particles of around 28 nm with more than 95% purity.

Both antigens are produced using Good Manufactory Practices and high quality standards at the CIGB facilities. The introduction of the HBcAg increases the antigenic representation in the resulting formulation and at the same time exerts an adjuvant and immunomodulatory effect on the resulting immune response to NASVAC.

Regarding HBsAg, it has been used for years in the production of the Cuban recombinant (r-HBsAg) Heberbiovac HB vaccine, which since its introduction in the market in 1990, has exhibited an immunogenic strength higher than other preventive hepatitis B vaccines. The r-HBsAg derived from the Cuban *Pichia pastoris* yeast strain transformed by genomic insertion of "s" gene to express r-HBsAg is molecularly identical to that derived from the *Saccharomyces cerevisiae* plasmid constructions used by most of the other vaccines. However, other features are different for the active antigenic ingredient of the Cuban vaccine, such as:

- Milder purification process (patented) designed to preserve HBsAg-r molecule integrity, including:
    - adsorption on diatomaceous earth (Celite)
    - preparative immunoaffinity chromatography (on selective and specific monoclonal antibody)
- Significant amounts of aggregated HBsAg-r particle clusters

These characteristics are now considered relevant for the high immunogenic strength of the vaccine.

Heberbiovac HB, the commercial vaccine containing HBsAg produced at CIGB, has an increased capacity to promote immune responses as compared to other commercially available vaccines (1,2). In the HBsAg production process, a fraction of the antigen is produced in a significant amount as aggregated particles, which can also be obtained by other procedures (3). A higher immunogenicity in mice has been demonstrated as compared to the non-aggregated form of the antigen. The aggregated antigen has an increased capacity to promote delayed type hypersensitivity responses (DTH) (4), total IgG antibody and specially a higher IgG2a subclass response in mice. This pattern correlates with enhanced CTL and Th responses, with demonstrated efficacy for viral clearance (7).

Heberbiovac HB has a higher capacity of inducing lymphoproliferative, T $CD8^+$ $\gamma$–IFN secreting cell (CTL), T helper 1 (Th1 polarized) in mice and total serum antibody responses as compared to the rest of commercial vaccines and a higher humoral and cellular immune responses induced in healthy vaccine recipient volunteers as compared to the HBV vaccines of concurrent providers (unpublished data).

Considering this background we proposed the potential therapeutic of CIGB´s *Pichia pastoris* derived antigen, based on the demonstrated differential properties of the Cuban vaccine's active ingredient, the r-HBsAg, referred above. These indications, that will be explained in details below, led to the hypothesis that NASVAC, applied by intranasal (IN) route, may subvert tolerance to the HBV surface and core antigens by inducing a strong specific humoral (anti-HBs and/or anti-HBeAg seroconversion) and cellular responses, resulting in an effective control of the virus and ultimately, it may lead to the reversion of the liver damage.

NASVAC constitutes a simple mixture of HBsAg and HBcAg in the proportion 1:1, free of preservatives or any other substance apart from the salts comprising the phosphate buffered solution. Considering this general background, we propose the potential therapeutic use of NASVAC, based on the particular properties that will be exposed bellow.

In summary, NASVAC has shown the capacity of inducing lymphoproliferative, antibody, T $CD8^+$ $\gamma$–IFN secreting cell (CTL), T helper (Th1 polarized) and “professional” antigen presenting dendritic cell (DC) responses in mice as well as detectable immune responses against both antigens in healthy volunteers with an excellent safety profile.

### 2.5 Fundamental background

About 5-15% of the HBV infected patients develop CHB characterized possibly by a deficient B, T and DC immune response (IR), by a mechanism not yet completely understood. The life expectancy of infected patients is shortened by the risk of developing cirrhosis, hepatocellular carcinoma or both, but some remain as asymptomatic HBV carriers.

The clinical and virological recovery from acute, self-limited HBV infection is associated with neutralizing serum antibodies (5) to HBsAg and the induction of strong cellular responses. The significance of CTL activation for the elimination of infected cells is further supported by adoptive transfer experiments of HBsAg specific CTL in HBV transgenic mice, which results in acute immune-mediated hepatitis (6).

On the other hand, the induction of HLA class I- restricted T-helper cells, i.e. specific T-cells that release TH1 cytokines regulate CTL activation and neutralizing anti-HBsAg antibody production, may be critical for viral clearance (7). Interferon (IFN) alpha (8) and Lamivudine and other nucleoside analogs (reverse transcription inhibiting oral nucleoside), are accepted as effective therapies for chronic hepatitis B. The

enhancement of HBV-specific immune reactions to protein-based immunization could also have some beneficial effects (9,10).

Lipopeptide based therapeutic vaccine candidates ( 11 ) showed initial promise, as well as the immunization with a vaccine containing the expression product of the upstream pre-S2 HBsAg gene region and DNA mediated immunization (12). Early disappointing results in the strategy of lipopeptides enhancing a selected CTL epitope when evaluated in CHB patients, highlights the necessity of somehow overcoming T-helper deficiency (13). PreS2/S vaccination of HBV carriers led to a reduction in HBV replication or clearance of virus in 30% of treated patients. Moreover, it was demonstrated that vaccine therapy does not induce the selection of escape mutants in the HBsAg 'a' determinant (14).

The increase in the course of HBsAg vaccination using Hepagene (Aventis Pasteur, France) until 8 to 12 administrations has also resulted in nearly 40% of virus clearance in difficult-to-treat CHB patients (15). To stimulate MHC class-I restricted CD8 (+) CTL that may destroy HBV-infected cells in patients with CHB, endogenous peptide processing and presentation is required ( 16 ). Intradermal (ID) HBsAg vaccination has evidenced high seroconversion rates on vaccine non-responders inoculated intramuscularly (IM) with 3 to 8 micrograms of HBsAg vaccine doses (17). ID is the alternative route most frequently used.

The HBcAg was obtained in E. coli as a virus-like particle of 28nm size with a high degree of purity. Its mucosal immunogenicity was evaluated in mice, evidencing the higher immunogenicity after nasal administration (18).

All results containing preclinical and clinical data of NASVAC is provided along with the current protocol as Annexed information (See Corresponding Protocol Annex).

## 2. 6 NASVAC: summary of clinical and preclinical data

### 2. 6.1 Preclinical toxicity studies

Extensive preclinical characterization of NASVAC in laboratory animals has been carried out. The preclinical toxicology studies were conducted by the preclinical testing group at the Animal Facilities of the Center for Genetic Engineering and Biotechnology, Habana, Cuba using quality control released batches in rats and mice produced at the Center for Production of Laboratory Animals (CENPALAB, La Habana, Cuba). The toxicology studies comprised an Acute Toxicology Study as well as the Evaluation of Mucosal Irritability. The studies demonstrated that the HBcAg-HBsAg vaccine candidate is innocuous in terms of irritability in repeated administration and also after acute toxicity testing, both after nasal administration (see corresponding file at the dossier).

*In summary:*

A first study to evaluate the nasal irritability was conducted in albino, Sprague-Dawley young female rats concluded that the formulation is not irritant to the nasal mucosa. The conclusion is based in the following points:

-Animals kept a somato-motor activity and responded according to the reported for healthy animals of these specie. No clinical signs related with irritation at the administration site were reported in any treated group.

-Normal food consumption was observed according to the reported data for the specie and sex used in the trial. The observed weight loss was not related with the product but because of the handling stress.

-No irritation sign was observed macroscopically in the administration site. No abnormalities were reported in any organ.

-Irritation index was minor than one (<1), reported findings were related with the normal physiological response of the nasal mucosa.

According to the regulated scale for these studies the index obtained classify the substance HBcAg/HBsAg as a non irritant product for the nasal mucosa.

A second study to evaluate the acute toxicity also evidenced after a detailed analysis that the formulation is non-toxic and that the events reported are the results of the normal physiological behavior as a consequence of administering the product under evaluation. This conclusion was based in the following points:

-Animal consumptions remained stable according to the reported data for the specie and sex used in the trial. There was a progressive increase in the body weight for both sexes.

-There were no etiological or anatomical alterations, keeping a normal response in front of stimulations in all the studied animals.

-Microscopic observations confirmed the clinical observations and evidenced that there was no damage in any of the organs under study.

-The histopathology study demonstrated that there was no irritation at the nasal mucosa even when the administered dose was 90 times superior to the therapeutic dose, there was no damage in lungs or brain, and the effects found at the inoculation organs constitute a physiological response to the administration stimulus.

**2.6.2 Preclinical immunogenicity studies**

An important number of trials in animals, demonstrated the accomplishment of the requirements to start the administration of NASVAC to humans (see the corresponding dossier enclosed and the related articles).

The formulation NASVAC, which combines the major antigens of HBV (HBsAg & HBcAg), was initially characterized using electron microscopy (EM). In line with previous literature, HBsAg and HBcAg individually had the physical appearance of virus-like particles using EM. Both particles were obtained and purified to a minimum of 95% purity and a size analysis revealed that both antigens were homogeneous in size and in the range of 20–30 nm. HBcAg was ~30 nm and HBsAg ~20 nm. It was possible to discriminate between HBcAg and HBsAg based on the well-recognized high electron density nucleus of the HBcAg particle (19).

Based on the characteristic nucleus of HBcAg we could easily observe the aggregation of HBsAg and HBcAg particles when mixed in the resulting HBs and HBc formulation. This aggregation could result from a natural tendency of both particles to aggregate, which was evident from the tendency of both antigens alone to form aggregates. Aggregates of various sizes, up to approximately 200 nm, have been observed (19).

***Serum IgG antibody response***

There was a significant increase in specific IgG response against HBsAg obtained in sera of mice nasally immunized with the HBs and HBc formulation compared with the group nasally immunized with the HBsAg in PBS. A comparison with control mice administered with HBsAg i.m. confirmed the superiority of the response induced by nasal immunization of the combined HBs and HBc formulation. In the case of the HBcAg-specific IgG response, the combined HBs and HBc formulation gave significantly higher responses than HBcAg alone, indicating that HBcAg increases the immune response to HBsAg and conversely HBsAg increases the response to HBcAg (19).

***IgG subclass pattern analysis***

Serum IgG subclass patterns were compared in the groups of mice immunized nasally with the combined HBs and HBc formulation and the corresponding parenteral control immunized with HBsAg in alum. Both C57/Bl6 mice and Balb/c mice revealed a strong IgG2a immune response to HBsAg after nasal immunization with the combined HBs and HBc formulation. As a result, the ratio of HBsAg-specific IgG1/IgG2a and IgG1/IgG2b were significantly decreased in both mice strains, indicating a Th1 switch induced by HBcAg. In the case of HBcAg-specific IgG subclass response, a similar pattern of a switch to an IgG2a immune response was observed with the combined HBs and HBc formulation. For HBcAg alone there was a very potent IgG2a and IgG2b antibody response, which is consistent with nasally administered HBcAg predominantly inducing a Th1 antibody response (19).

***Lymphoproliferative and IFN-γ responses after nasal or i.m. immunization with HBsAg and HBcAg***

The capacity of the nasal route to induce cellular responses in systemic compartments was evaluated by studying lymphoproliferative and IFN-γ responses of whole spleen cells to HBsAg and HBcAg after nasal immunization with HBsAg or HBcAg alone or the combined HBs and HBc formulation compared to i.m. immunization with HBsAg in alum. The IFN-γ ELISPOT study was conducted using the immune-dominant HBs(28–39) CTL peptide from HBsAg as previously described. Measurement of lymphoproliferative activity revealed the induction of strong proliferative responses in systemic compartments against both HBsAg and HBcAg after nasal-combined immunization. The antibody responses obtained with the combined HBs and HBc formulation were consistently higher than the responses induced by HBsAg or HBcAg alone, whether administered nasally and parenterally (19).

It was possible to detect IFN-γ secretion to HBsAg 1 month after a booster dose on day 90 using an ELISPOT study incorporating a re-stimulation protocol. A significant increase in the secretion of IFN-γ was obtained for the group immunized with the nasal-combined formulation compared with the HBsAg nasally administered in PBS and the parenteral control of HBsAg in alum (19).

In summary: a marked enhancement in antibody and cellular responses towards both HBsAg and HBcAg is observed after the nasal co-administration of a formulation containing a mixture of HBsAg and HBcAg. Although this synergism is also observed when HBsAg and HBcAg are coadministered i.m., the total antibody response was 1–2 logs higher after nasal compared to parenteral administration. Furthermore, the use of the nasal route enhanced the production of IgG2a, suggesting the ability of nasally administered HBsAg and HBcAg to favor a Th1 response. This feature held true when using either Balb/c or C57/Bl6 mice, despite the fact that the former have an overall Th2 bias (19).

The adjuvant effect of HBcAg for HBsAg could be explained in several ways. New properties may arise in formulations containing both HBsAg and HBcAg as a result of the ability of such mixtures to form self-organizing aggregated structures. Both HBsAg and HBcAg naturally aggregate into particles with HBsAg forming particles measuring 22 nm and HBcAg forming particles measuring 28 nm. Both antigens interact to generate aggregated structures ranging in size from 22 nm to 200 nm. The particulate nature of antigens is an important feature for the immunogenicity of nasally administered antigens (20-24).

Another mechanism that may explain how HBcAg can act as an adjuvant relates to its unique 3-D folding, which generates a regular spacing between repetitive spikes on its surface. This feature enables HBcAg to bind and activate a high number of naive B cells. Following binding of HBcAg, the cross-linking of immunoglobulin membrane receptors on B cells results in intracellular signaling, secretion of IgM and IgG and up-regulation of co-stimulatory molecules (25). Taking into consideration the very fast and specific uptake of HBcAg by B cells (25) aggregated particles may be taken up and processed by B cells preferentially, driving the HBsAg through a very effective route for antigen processing and presentation.

The above results are highly relevant to therapeutic vaccination for chronic hepatitis B infection. It has been suggested that the effect of serum anti-HBcAg antibodies in blocking the interaction between B cells and HBcAg may be an important factor in viral immune-pathogenesis (25). Mucosal administration of HBsAg and HBcAg is directed at avoiding this negative effect of systemic anti-HBcAg antibodies that are present as a general feature of chronic HBV infection. The protected compartment offered by M cells may enable the administered antigen to avoid the modulating effect of high serum anti-HBcAg titers.

Another point of interest regarding mucosal administration concerns the recent discovery of HBV replication in salivary glands and the secretion of HBeAg (26). These results suggest that oral tolerance may develop towards HBeAg or against HBcAg (which shares around 80% of the HBeAg primary sequence) by virtue of the HBeAg being secreted by the salivary glands, swallowed and treated by the immune system as a dietary antigen. It is hypothesized that mucosal administration of antigens in a Th1-immunomodulatory environment may help in the subversion of such oral tolerance (27).

Recent reports from Boni *et al.* reveal an important degree of T-cell functional recovery after antiviral treatment with Lamivudine (28, 29), suggesting that it is possible to rescue T cells from non-responsive states. Other reports demonstrate that in patients naturally recovering from HBV chronic infection, there is a rebound in cellular immunity that correlates with viral clearance (30). This raises the possibility of combining nasal vaccination with antiviral treatment as a means of overcoming non-responsiveness and tolerance to hepatitis antigens in chronically infected individuals. The interaction between HBcAg-activated B cells and T cells results in the development of a Th1 phenotype, followed by antibody isotype switching and the increased production of IgG anti HBcAg antibodies (25, 30). The same effect was obtained in the HBsAg immune response in the combined HBs and HBc formulation studied in two different mice strains (19).

There was a marked increase in the number of IFN-$\gamma$-producing spleen cells in mice immunized with the combined HBs and HBc formulation. The ELISPOT study using the P815 mastocytome cell line pulsed with the CD8+-restricted HBs(28–39) peptide indicated that nasal immunization with the combined HBs and HBc formulation activates antigen specific CD8+ cells, consistent with a strong Th1 response. This result is in line with the finding that B cells that bind viral capsids can prime cytotoxic T cells (30). We were also able to show that proliferative T-cell responses were enhanced using nasal administration of the combined HBs/ HBc formulation. This was evident from the strong adjuvant effect of HBcAg on the cellular immune response against HBsAg and *vice versa*.

New properties arise from the mixture of HBsAg and HBcAg as a consequence of the adjuvant and immune-modulating actions of both antigens. Nasal administration of both antigens in a combined formulation induces a heightened humoral and cellular immune response against both HBsAg and HBcAg.

Previous positive although still weak therapeutic effect in the literature using commercially available HBsAg-based vaccines and the higher immunogenicity of the CIGB HBsAg, along with the opportunity of generalized immune response after mucosal immunization, deserve a refined approach for evaluation in patients.

The present first controlled clinical trial in patient with the HBV surface and core antigens is intended to determine any therapeutic potential on chronically HBV infected patients.

### 2.6.3 Immunogenicity and immunotoxicological studies in HBsAg (+) transgenic mice

***Humoral and cellular immune response induction in transgenic mice is not associated to immune-pathological damage.***

NASVAC formulation has been developed and evaluated at CIGB for therapeutic purposes. The availability of transgenic mice expressing HBsAg enabled studies on the mechanisms responsible for B and T cell tolerance and assessment of strategies and therapeutic formulations with potential for persistent viral infection eradication. A report is annexed to the present Clinical Trial Protocol including immunological and histopathological evaluations in mice expressing HBsAg in all their organs. Histopathological study of organs from transgenic mice that generated an immune response as a result of the HBsAg and HBcAg (NASVAC) simultaneous co-administration in a combined formulation by the mucosal (IN) and parenteral (SC) routes evidenced no damage as a result of the immunization protocol including 10 intranasal/subcutaneous immunizations using NASVAC formulation.

The objective of the first Immunopathology Report in transgenic mice was: to characterize the immune-response induced by the combined HBsAg and HBcAg simultaneous co-administration by the mucosal and parenteral routes in the Tg HBsAg (+) transgenic mouse model and to study the effect of this response on the transgenic mice organs and tissues.

Specifically, a) to determine the necessary dose number for detecting antibody response against HBsAg in the transgenic mice sera; b) to determine the optimal dose number needed to detect antigen specific T cell response in the transgenic mice spleen cells and c) to study the immune response effect on the transgenic animal organs and tissues.

The induction of an anti-HBsAg response in transgenic mice allowed the unique opportunity of knowing if starting from the active immunization and using an inoculation schedule of 10 administrations of the NASVAC vaccine candidate by nasal and parenteral route simultaneously, it is possible to induce damage to the immunologic system organs as well as the most important organs of the animal, most of them expressing HBsAg constitutively.

As it is known, the HBV target organ is the liver and for this reason in the histopathological study this organ was investigated with priority for any evidence of damage. Additionally other organs were studied, among them the kidneys, where the presence of immune-complexes could be a damaging factor. The histopathological study was done according to the conditions described in the corresponding Annex.

As a result of this work, no alterations of the organs of the transgenic mice were observed that could be associated to the administration of this product both by IN and SC immunizations as proposed in the present clinical trial. Additionally, no damage was observed in the liver or kidneys, which could be the most likely organs to be damaged. Other organs where the presence of HBsAg has been demonstrated were not damaged either. The corresponding Annex contains the summary of the technical report of the Pathology Group of the Center for Genetic Engineering and Biotechnology. This work has been carried out replicated, obtaining similar results in the group treated with the NASVAC therapeutic vaccine. A similar study was performed later using subcutaneous administration without alum and the same protocol and there was no sign of immunopathology.

It is important to highlight that the pharmacologic dose that these mice receive is much higher than the dose that a patient would receive. Additionally, the administration was achieved with a frequency that is the same to be used in the clinical studies (10 inoculations every 14 days).

It must be emphasized that a comparative study was undertaken with 4 different groups, where the vaccine candidate was administered to two transgenic animal groups and also to Balb/c non transgenic control groups. In the histopathology study only isolated conditions not associated to the treatment were observed in the negative control (transgenic as well as non transgenic) groups, while in the immunized (tg or not) no damage was observed in the hepatocytes or kidney tissues (see corresponding Annex).

A second histopathology study in the same report evidenced that the transgenic mice immunized with ten administrations every 14 days, with a similar pharmacologic dose to the previous study, there was no damage in the spleen nor in the thymus, which are organs from the immunological system. Additionally, it was observed absence of damage of liver, kidney, lung, heart, central nervous system or trachea.

The administration of 10 inoculations using a pharmacologic dose which is much higher than the dose that will be used in humans and with a similar treatment schedule -10 doses every 14 days- did not evidence damage to the animal model that is the closest to the chronic infection by the hepatitis B virus, the transgenic mouse expressing this antigen at similar levels to those present in the patients' blood. It is important to point out that from the several tg mice reported in literature, the present is probably the tg mice with the higher concentrations of HBsAg in sera as it was produced and selected to be comparable in HBsAg levels to the higher HBsAg concentrations found in patients sera.

In summary, from the first set of studies using tg mice to explore the immune mediated damage in mice expressing high concentrations of HBsAg in sera and organs, it was possible to conclude that a) The administration of at least 5 doses of the formulations containing simultaneously HBsAg and HBcAg by the IN and SC routes allowed the detection of humoral and CD8+ cellular responses in all the transgenic animals, as evidenced by the $\gamma$□IFN ELISPOT; b) The dynamics of the humoral and cellular response appearance was slower in the case of the tg as compared to the normal mice following the same humoral response pattern as expected and c) The administration of 10 inoculations using a pharmacologic dose which is much higher than that to be used in humans and a similar treatment schedule -10 doses each 14 days- did not evidence any damage in the main target organs and in the immune system of this animal model.

***Adoptive transfer of cellular immune response from Balb/C donor to HBsAg(+) transgenic mice is not associated to immuno-pathological damage.***

The immunotherapy based in the development of vaccine candidates constitutes one of the most promissory strategies in the therapy of chronic hepatitis B. The effectiveness of these candidates depends on the generation of a potent humoral and cellular immune response, capable to overcome the tolerance established in chronic patients against the viral antigens.

Some reports in literature demonstrated that the passive transfer of bone marrow from a naturally immune donor is effective in the viral clearance. These works indicates the relevancy of the cytotoxic T cell response in the resolution of the acute and chronic infection. However, a concern in the development of therapeutic vaccines is the potential immuno-pathological damage related to the generation of a potent cellular response against the viral antigens expressed in the liver and other organs as a consequence of the chronic infection.

In experiments with transgenic mice it was reported that the deposition of immune-complex antigen-antibody specific against HBsAg could cause damage at liver and kidney levels. Based on all the above evidences, the present work explore with more details safety aspects related with the nasal/parenteral administration of the vaccine candidate Nasvac. With that purpose we have simulated a chronic patient that develops a potent immune response against the surface and nucleocapsid antigens of HBV after the administration of said vaccine candidate using HBsAg transgenic mice that received a passive transfer of immune cells: intraperitoneal administration of total splenocytes coming from non-transgenic Balb/C mice that were immunized nasally and parenterally with three doses of a formulation containing HBsAg and HBcAg antigens (Nasvac).

The main objective was to study the effect of the immune response generated by nasal/parenteral vaccination with Nasvac, using the model of adoptive transfer of immune cells to HBsAg transgenic mice.

Specifically, a) to evaluate the effect of the transferred immune response on the concentration of HBsAg circulating in the sera of transgenic mice (antigenemia). b) to study, in the context of the transgenic mice, the kinetic of the transferred HBs-specific antibody response and c) to evaluate the effect of the adoptive transfer of immunity in the hemochemical and histological parameters, as a measure of the safety of the immune response induced by Nasvac immunization.

The experimental conditions are explained in the corresponding Annex to the present clinical protocol. In summary, the group of tg mice that received immune splenocytes showed a significant decrease of HBsAg concentration since the first week post-transfer evaluation. We found statistical differences between time cero and the second and third weeks post-transfer ($p < 0.05$, Student t test). Starting from the week number four, HBsAg concentration increased again, indicating that the control established by the transferred immunity was disappearing. Since this time point and until the week eight, we didn't find statistical differences in the antigenemia with the level reported for time zero.

In the case of the mice receiving splenocytes with HBs-specific immunity a marked decrease in the HBsAg concentration was detected in sera, notably between days seven and twenty-eight. However, for mice receiving placebo splenocytes or PBS 1X, although fluctuations in the serum HBsAg concentration were detected, there was no statistical difference from the concentration reported for time zero. As we showed in figure 1, values obtained for this group of animals are always above 5μg/ml. These results indicate that efficient decrease of circulating HBsAg is possible using adoptive transfer of immunity in the transgenic mice model. The antigenemia control established by the transferred immune response was effective for three weeks after a single splenocyte transfer.

Regarding HBsAg-specific IgG response in sera, all mice that received splenocytes with previous immunity against HBsAg showed a specific response of IgG, except one mouse, for which we didn't detect response. This result is in concordance with the antigenemia results obtained for this specific mouse, in which although we detected a slight decrease in the HBsAg concentration, it was not important. In the case of animals with HBs-specific IgG response the titers were high ($> 10^4$) and began to decrease after the third week, this could be related with the antigenemia increase found around the fourth week (day 35 on). The groups of treatment that received placebo splenocytes or saline solution did not shown HBs-specific antibody titers.

The hematological studies were based in the detection of alanine amino transferase (ALT), alkaline phosphatase and creatinine after eight weeks post transfer, a relevant date for hematological abnormalities in literature works. These specific markers are commonly used to measure liver and kidney damage. We known that the transgenic mice employed express the HBsAg constitutively in the liver, kidney and other organs, that's why constitutes a concern the potential damage induce by the presence of a potent specific immune response.

Statistical comparison between treated (mice that received anti-HBsAg immune splenocytes transfer) and not treated (mice that received placebo splenocytes or saline solution transfer) did not yield significant differences for the evaluated parameters.

Histological studies performed at week eight post-transfer, did not evidence differences regarding the animals weigh neither the weigh of the main organs between treated and not treated. The extracted organs were included in formol for histological analysis. As a result of this work, no alterations of the organs of the transgenic mice were observed that could be associated to the adoptive transferred immunity, additionally, no damage was observed in the liver or kidneys, which could be the most likely organs to be damaged. Other organs where the presence of HBsAg has been demonstrated were not damaged either.

Taking into account all the results we concluded that the adoptive transfer of immunity performed in our conditions was effective, decreasing the levels of circulating HBsAg in sera, safe, because it did not cause hystological damage in the studied organs. These results demonstrated the safety of the nasal/parenteral administration of Nasvac candidate in the studied model.

## 2.7 Clinical experience

***NASVAC administration to healthy volunteers was safe and immunogenic***

A Phase I Placebo controlled study was conducted with NASVAC in order to evaluate the safety and immunogenicity in 20 human healthy volunteers. NASVAC evidenced a very good safety profile, comparable with the placebo used in the study: saline solution.

The immunogenicity of NASVAC was also evidenced in the same study: All vaccinated volunteers (100%) responded to HBcAg, in the case of the anti-HBsAg, 75% of the volunteers generated a seroprotective response and 50% of those were hyper-responders (more than 100 UI/L of anti HBsAg antibodies). There was no immune response against any of the antigens in the placebo group (see corresponding Annex).

***The use of NASVAC for treating CHB patients was safe and evidenced preliminary efficacy***

In addition, NASVAC therapeutic vaccine candidate has been studied in treatment-naïve chronically infected patients. The formulation -previously studied in healthy volunteers- was administered at 100 mcg per antigen per dose, ten times every two weeks, to HBV chronically infected patients. The formulation was applied -in a first cycle- five times by intranasal route and -in a second cycle-, another five times, simultaneously by intranasal and subcutaneous routes, to the same patients. Patients were characterized as: HBeAg positive (7) and negative (13). ALT baseline levels were normal in 12 patients and abnormal in 8 patients. Patients with antigenemia for more than 6 months and detectable HBV were considered for

treatment after previous altered transaminase determinations in the last six months or by histological abnormalities, recommending treatment.

After dose number ten, there was a 66,6 percent (4/6) of transaminase normalization to levels under the upper level of normality (ULN) for patients starting in abnormal transaminase baseline levels. Biochemical response after treatment largely surpassed the success percentage value required to the clinical trial. Importantly, none of the patients with normal baseline ALT levels had abnormal ALT values after ten doses even when most patients' ALT values peaked during treatment, mainly during the first cycle. The peak after starting therapy has been associated in literature to virological clearance as a result of immune activation in post-treatment period.

Virological response was assayed after five and ten doses. The HBeAg negative group of patients behaved better in terms of HBV elimination by quantitative PCR (detection limit 250 copies/mL) compared to HBeAg positive group. From 13 HBeAg patients receiving five intranasal doses of NASVAC, six patients reduced the viral load to undetectable levels for 46% just after five intranasal doses. None of the six patients clearing the virus were over the ULN for ALT after the fifth or after dose number 10, even when four of them had a mild ALT flare during the first cycle of IN administrations, suggesting a quick immune response. The absence of ALT increase during treatment in two patients clearing the virus suggests that non-cytolitic mechanisms were also activated. For patients clearing the virus, ALT increases ranged between 1.5 and 3 times the ULN, such increases where in one or two consecutive determinations only, suggesting that the potentially curative effect of vaccination was associated at least to small and quick flares of ALT levels. Such increases were not clinically apparent as patients didn't report any adverse event during the course of the vaccination, and physicians didn't detect any related symptom, suggesting that the increases in ALT levels were not clinically apparent. Also, due to the temporal relation to the starting date of the protocol, it is reasonable to predict that it was associated to the vaccination. From literature is known that specific immune stimulation has been related to ALT increases and virological response in CHB patients.

In support of the results obtained after the end of treatment, the evaluation of the 6 month time follow up (time 12 months) evidenced a further decrease in the levels of HBV DNA to undetectable levels in 50% of total patients, being outstanding the behavior of HBeAg negative patients that reduced the viral load in 8 of 13 patients. HBeAg positive patients also evidenced strong reductions in viral load however the reduction to undetectable was reduced compared to HBeAg negative patients, which is understandable based in the fact that the baseline viral load values were much higher for HBeAg positive patients. For both groups ALT values evidenced a generalized normalization reaching 100% at month 9 and 95% at month 12..

Further clinical research is required as in case the concept of therapeutic immunization is probed, a large amount of world population will find in therapeutic vaccination a new finite and affordable treatment to overcome current situation.

*All previously presented facts induced the following **working hypothesis**:*

*The administration of NASVAC to CHB patients may subvert tolerance to the surface and core antigens (Ag) of HBV inducing specific immune responses (antibody seroconversion to anti-HBs and anti-HBe), T cells (Th1, CTL) and antigen presenting cells (APCs). Such immune response will favor the control of virus replication and will normalize the transaminases (ALT and AST) in a similar or superior extent to the currently approved treatment Interferon alpha 2b, associated to a lower number of adverse reactions.*

***Rationale of the hypothesis:***

*In respect to the nature of HBV chronic infection*

-There is a clear dichotomy in hepatitis B infection: patients resolving acute hepatitis display strong polyclonal and multispecific helper and cytotoxic T-cell responses against HBV nucleocapsid, polymerase and envelope proteins, these cellular responses are weak or even undetectable in patients with chronic HBV infection (Webster et al., 2004).

The CD8+ cytotoxic T lymphocytes (CTL) can kill infected hepatocytes by various mechanisms (perforin, Fas-L and tumor necrosis factor $\alpha$ (TNF$\alpha$). However, the lack of massive hepatocyte lysis during recovery from infection suggests that HBV infection is controlled by mechanisms other than lysis. Indeed, a recent study in humans confirmed that the number of CD8 + T cells infiltrating the liver is not related to the level of hepatocyte lysis (31). Studies in mice and chimpanzees have shown that HBV is eliminated from hepatocytes largely due to the secretion of Th1 cytokines such as IFNs and TNF$\alpha$ (32; 33). During chronic infection, levels of Th1 cytokine production by peripheral blood cells stimulated with antigens are very low, which is not the case during acute infection (34-37).

-Nevertheless, cytotoxic T-cell responses against the virus of equal intensity to those found in recovered patients have been observed in patients with chronic infection resolving spontaneously (10-15 % of patients) or responding to IFN-a treatment (38). An increase in CD4+ and CD8+ T-cell responses specific for HBV has also been reported in chronically infected patients treated with Lamivudine, suggesting that it may be possible to induce an immune response in these patients (29, 39).

-The concept of immunotherapy, which involves reactivating deficient immune responses to control viral replication, has been directly validated during transplantation of bone marrow from donors with anti-HBV immunity to recipients with chronic HBV infection (HBsAg-positive), curing the infection (40; 41). The aim of treatment is therefore to decrease levels of viral replication and to eliminate infected hepatocytes. For

this reason, new strategies for immunotherapy by vaccination target not only the induction or stimulation of CD4 + and CD8 + T-cell responses, but also the induction of proinflammatory cytokines capable of controlling viral replication.

A number of reasons specifically related to the present proposal:

*Regarding the HBsAg*

-A certain proportion of the HBsAg particles produced by the CIGB *P. pastoris* recombinant strain form aggregates which are more immunogenic in mice, in terms of specific antibody production and cellular immunity recruitment. These aggregates are present in the NASVAC TVC and have also been purified and characterized.

*Regarding the HBcAg*

-It is also produced as a recombinant protein VLP in E coli, with a strong immunogenicity by systemic and mucosal routes and similar folding compared to the naturally produced antigen.

-HBcAg is responsible of an important proportion of anti HBV cellular immune response, and anti-HBcAg cellular immunity has shown therapeutic value in experiments of passive transplantation.

-It has been documented the unique mechanism of HBcAg to activate the immune system related to the uptake of B-cells and the ulterior development of these B cells in very efficient professional APC by Ab cross linking on their membranes.

-A high Ab titer against HBcAg in blood is a normal feature of patients with chronic Hep B, the potential use of HBcAg through the parenteral route could be negative due to pre-existing immunity.

*Regarding the mucosal administration of both antigens (nasal administration of NASVAC):*

-Pre-clinical experiments in mice showing a higher immunogenicity and increased capacity of NASVAC to promote cellular and humoral (superior IgG2a) responses support the expectation for the induction of a therapeutic (humoral/cellular) immune-response in chronic HBV infected patients. This pattern has been shown to correlate with enhanced CTL and helper responses, with demonstrated efficacy for viral clearance in hepatitis B therapeutic approaches.

-there is a high frequency of professional antigen-presenting cells of airway surfaces reaching 700 DC per $mm^2$.

-HBV main target is liver, however, the virus has been detected in several organs related to the mucosal immune system (salivary and thyroid glands, gonads).

-The immunity induced in systemic compartments by parenteral administration only marginally covers the organs associated to the mucosal immune system.

-Mucosal administration of antigens is able to generate mucosal as well as systemic responses, activating the immunocytes associated to mucosa –approximately 80%.

-Nasal immunization is one of the most effective routes in terms of antigen amount needed to induce parenteral and mucosal immunity, -even in remote tissues as genital tract.

-Mucosal immune system has shown to remain undepressed in front of concomitant immune depressions in the systemic compartments during chronic infections.

-Impairment of DC of CHB patients as well as the non-responsiveness of the effectors B and T cell responses from systemic compartments of CHB patients.

-The mucosal route provides the very protected environment of M-cells and the tonsil crypts, enabling the induction of the immune response protected from systemic tolerogenic environments and protected from systemic immunity.

*Regarding adverse reactions*

-It has been widely recognized the adverse reactions of interferon therapy, none of them have been associated until now to the use of any therapeutic vaccination under study, including NASVAC.

## 3. Ethics and Medical Deontology

### General ethical considerations

The trial is justified from the ethical point of view because:

- The study is carried out according to the principles of the Declaration of Helsinki on investigations with human beings for therapeutics purposes. The patients will receive the medical care and the normal general treatment that is applied in these cases.

- CHB is a slow developing disease. The fact of being 6 – 12 months without any of the specific recognized treatments will not affect significantly the patients' evolution. Besides, follow-up will be very cautious and upon any sign of disease progression, the patient will be withdrawn from the study treatment and given a recognized specific anti-hepatitis therapy (interferon alpha or Lamivudine). In all cases patients included in the trial, after its completion will receive one of these treatments, if the principal patient and investigator decide.

- It is possible that the patients obtain benefit from their participation in the study since there are reasonable evidences that vaccination can improve their condition according to phase I results.

- Treatment of CHB patients with NASVAC, according to the results obtained at CIGB, does not represent additional risk for the patients. It is a quite safe intervention, with a very low probability of associated adverse reactions. Doctors can properly treat the possible adverse reactions and a favorable risk-benefit balance justifies them.

- Written consent of individuals will be requested, after properly informing about the objectives of the investigation, treatment to be subjected and possibility to refuse to participate in the trial at any moment without consequences to his medical care. When publishing, the confidentiality of the individuals' personal data will be guaranteed.

- All patients unable to satisfactorily control the viral load will be treated with available antiviral drugs, according to their physician decision, independently whether they belong to the control or treatment groups.

- There are preliminary evidences of safety and efficacy after the first clinical trial using NASVAC in chronic hepatitis B patients, conducted in Dhaka, Bangladesh, as previously described in the present protocol.

**Trial authorization and approval**

The trial will be carried out in agreement with the regulations established in Bangladesh.

The protocol will be approved by the Ethical Committee of the Faculty of Medical Sciences related Hospital and other relevant authority of Bangladesh.

**Explanation on how the patients will be informed and their consent will be obtained**

The patients will receive explanation about their disease and the characteristics of the trial, objectives, prospective benefits, risks and inconveniences that could be presented related with the treatment. The potential advantages of the proposed treatment and possible adverse effects will be explained to the patient or relatives, specifically the medical and nursery cares required for these cases.

The patients will decide on their participation in the trial freely, through written consent (see Annex 2), in case of abandonment of the treatment by self-decision, their medical care will not be affected.

## 4. General design

**Type of trial:** Open phase II-III clinical trial.

**Design of the trial**

The population sample to be tested will be of 160 CHB patients, with variable serum transaminase levels, without treatment for at least 6 months, selected according to inclusion and exclusion criteria and allocated in two study groups (SG). The first study group, SG1 will include 80 CHB patients (HBeAg (+) and HBeAg (-)) that will be treated with 1 mL NASVAC (100 mcg per antigen, per dose per patient), administered in two cycles of inoculations, the first one by the intranasal route with five intranasal inoculations every 14 days each. During the second cycle patients will receive the same dosis (100 mcg per antigen) by the intranasal route and additionally the same dosis by the SC route,

The SG2 will be treated with Alpha-Interferon at standard dosis (weekly injection by SC route).

The treatment is discontinued for all patients of SG1 after completion of both cycles. The evaluation will continue during one year, but an interim report will be released after 5 and 10 administrations.

During the whole treatment period up to one year from the beginning of treatment, the systematic assessment of patients is launched to determine to what extent the treatment has driven them to generate the capacity to control the HBV load. This information is included in Figure 1.

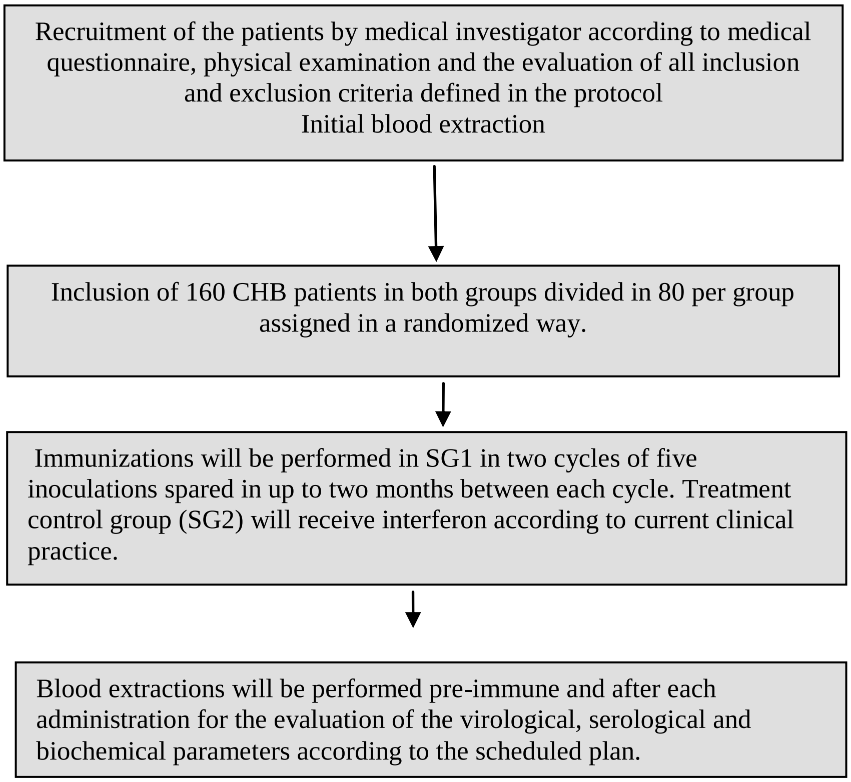


**Figure 1.** General Algorithm of the trial

## 5. Selection of subjects

### Population

Patients of both sexes, from 18 to 60 years-old with diagnosis of CHB will constitute the study population. These patients must, at least, be free of any specific treatment for CHB in the past 6 months before the inclusion in the study.

### 5.2 Diagnostic Criteria

The diagnosis will be established by

- HBV surface antigen (HBsAg) in serum for more than 6 months
- Presence of HVB DNA in serum with a detectable viral load according to quantitative PCR analysis

- One abnormal ALT value in the last year before immunization or history of chronic hepatitis verified by biopsy (Biopsy will not be a requisite, however, it should be stimulated for those requiring such procedure according to current clinical practice in Bangladesh)

### 5.3 Inclusion criteria

- HBsAg+ serology for more than 6 months before the beginning of the treatment.

- HBeAg (-) patients, with a) baseline HBV DNA ≥ $10^3$ and b) raised serum ALT (> 1 x ULN) or significant hepatic necro-inflammation and or fibrosis (HAI-NI≥4 and/or HAI-F ≥2), or stiffness >7.2.

- HBeAg (+) patients, with a) baseline HBV DNA ≥ $10^4$ and b) and raised serum ALT (>1.5 ULN) or significant hepatic necro-inflammation and or fibrosis (HAI-NI≥4 and/or HAI-F ≥2) stiffness **>7.2**
.

- 18 to 60 years-old patients of both sexes.

- No specific hepatitis B treatment at least for 6 months previous to the inclusion

- Voluntary signed informed consent to participate in the trial

### 5.4 Exclusion criteria

- Condition of asymptomatic HBV carrier, cirrhotic or primary hepato-cellular carcinoma patients
- Positive serological markers for hepatitis C or hepatitis delta virus.
- Positive serological markers for HIV
- Previous specific anti-hepatitis treatment in the last 6 months.
- Critically ill patient, heart or renal failure, hypertension, hyperthyroidism, epilepsy, immunodeficiency diseases, malignancies or any non-controlled systemic disease.
- Pregnancy or nursing women. Women in fertile ages without any contraceptive methods.
- Known severe allergic conditions (degree III or IV asthma, urticaria, dermatitis, bronchitis, etc. or hypersensitivity to any of the ingredients present in the preparation such as thiomersal.
- Severe psychiatric dysfunction or another limitation that prevents the patient’s consent.
- Autoimmune diseases (such as lupus, rheumatoid arthritis, multiple sclerosis, non controlled diabetes mellitus type 1)

- Other hepatic diseases of different etiology (such as alcoholism, auto immune hepatitis, toxic hepatitis, Wilson diseases, hemochromatosis)

- Treatment with immune suppressive disorder or administration of immune suppressive-immune modulator drugs (including steroids) during or in the 6 months previous to the study.

- Very high transaminase levels at the beginning of treatment (ALT over 500 U/L) suggesting a not stable disease with risk for patient's health or acute flares over 15 times the upper limit of normality.

### 5.5 Exit criteria or interruption of the treatment after inclusion

- Voluntary abandonment of the patient.

- Appearance of any of the above-mentioned exclusion criteria.

- Patients with worsening of the liver pathology in comparison with baseline levels, according to the investigator's criteria.

- Severe adverse reactions according to WHO guidelines

- Feverish status (more than 37.8 °C) at the moment of vaccine administration or in the 24 hour before, or any suspected or confirmed infectious diseases that results in the suspension of three vaccine administrations during the course of the immunization schedule.

- Patient's death.

Patients, once included, will be considered in the analysis even if they interrupt the treatment. Therefore, no case report form will be eliminated.

## 6. Treatment

**Products to be used**

### General characteristics of the NASVAC TVC

- NASVAC TVC composition and specifications are contained in the dossier provided with the present protocol. Briefly:

per 1 bb (500 mcL) NASVAC formulation contains:

| Component | NASVAC TVC (per 0.5 mL) |
|---|---|
| Recombinant HBsAg, produced in *Pichia pastoris*, immunopurified. | 50 μg |
| Recombinant HBcAg, produced in *E coli*, purified over 95% of purity. | 50 μg |
| Sodium chloride | 4.0 mg |
| Dibasic sodium phosphate (anhydrous) | 5,6 mg |
| Dehydrated monobasic sodium phosphate or Monohydrated monobasic sodium phosphate | 0.62 mg or 0.55 mg, respectively |
| Water for injection, up to | 0.5 mL |

**Treatment schedule**

The NASVAC TVC will be administered **to SG1** during the first cycle in a final volume of 1.0 mL (100μg of HBsAg and 100μg of HBcAg) by intranasal route with 8 actuations of 125 μL until completing . The second cycle will continue the IN administration in the same dosis and frequency and simultaneously the same treatment will be administered by the SC route. The device to be used (Valois VP7D spray pump) will be actuated with five minutes intervals between actuations in the same nostril.

The pegylated IFN will be administered **to SG2** weekly by subcutaneous route in the established dose (180μg) .

**Rules for the use of concomitant treatments**

- Any specific anti hepatitis treatment should be avoided, as well any other investigational drug.
- Other drugs that can affect the immune system should also be avoided (corticosteroids, immune-suppressors, immune-stimulants, cytokines, etc.).

**Measures to guarantee safety in the manipulation of the products**

1. Experienced personnel will carry out the administration of the medications in the clinic.
2. NASVAC and IFN vials should be stored at 2 to 8 $^{o}$C (domestic refrigerators) until use.
3. The access to all products will be limited and controlled by the principal investigator or the person designed by him.

4. The vials that are not used, as well as empty vials, should be returned to the sponsor.

5. The product should not be used for another purpose besides this clinical trial.

**Measures to promote and control that the instructions and prescribed rules are followed**

1. An included- and not included-patients register will be kept.

2. The sponsors will verify storage conditions and use of the products in the quality control visits by monitors or by any other persons officially designated by the sponsors.

3. The treatment will be controlled through a control card, where each administration will be registered.

4. The investigators will know the analysis certificate of the products in order to begin trial.

5. The investigators, properly trained for it, will carry out the whole treatment.

6. A physician that includes a case will continue his follow-up and will be responsible for the suitable control of the complementary tests.

## 7. Evaluation

**Evaluation criteria regarding immunogenicity / efficacy**

**Main variable**

The main variable of efficacy is the HBV DNA viral load quantification in serum.

**Secondary variables**

Serological markers: HBeAg and anti-HBeAg, HBsAg and anti-HBsAg

Biochemical markers: liver transaminases (ALT and AST)

Other parameters regarding basic hematology and clinical biochemistry (bilirrubin, alkaline phosphatase etc).

Histology: histological grade and stage

**Control variables**

- Age
- Sex

– Weight

– Body mass index

– Duration of the disease

– Initial viral load

– Smoking and other toxic habits

**Variables used to measure the effects and periodicity of the determinations**

The clinical parameters will be evaluated by the investigator taking into account the information that the patient offers and the physical examination. The laboratory parameters will be measured by the usual methods of clinical laboratory. The information will be registered in the case report forms.

In the following table the evaluation parameters and their frequency are included.

**Evaluation criteria regarding safety**

All AE that appear during the study will be recorded in the Case Report Form of each individual.

All medical incidents that occur in the first hour after each administration of the vaccine, causally related or not with the vaccination will be recorded.

The rest of the observations will be evaluated in the follow up visits every 14 days.

The observation includes medical questionnaire of the patient, inspection of the injection site, axillary's temperature, and registration of all symptoms and signs referred by the patient. Explanation of how to act in case of abandonment or exit in the trial

The patients that abandon the trial will always be evaluated, except if the investigator looses contact with him. The information will be registered in the Case Report Form (CRF), including the cause of exit.

Quality control of the evaluation procedure

The clinical and laboratory evaluations will be preserved, in order to be examined by external quality controllers.

All primary data should be collected and conserved during the study.

The frequency of specific determinations as well as the type of determination per specific date according to the protocol are presented below: (Grey boxes: Determinations will not be performed).

| Test | Baseline (not more than 2 months before starting treatment) | Week 12 | Week 24 EOT-V | Week 36 | Week 48 EOT-I | Week 72 | Week 96 | Reference Values (M: male, F: female) |
|---|---|---|---|---|---|---|---|---|
| Hemoglobin | | | | | | | | M: 12-17 g/dL<br>F: 11.5-15.5 g/dL |
| Hematocrit (PCV) | | | | | | | | 37-54% |
| Leukocytes | | | | | | | | 4-10 x $10^9$/L |
| Neutrophils | | | | | | | | 40-80% |
| Lymphocytes | | | | | | | | 20-40% |
| Eosinophils | | | | | | | | 1-6% |
| Basophils | | | | | | | | Up to 1% |
| Plateles | | | | | | | | 150-400 x $10^9$/L |
| Random Blood Sugar | | | | | | | | 3.9 – 7.7 mmol/L |
| S. Creatinine | | | | | | | | M: 60-130 μmol/L<br>F: 53-120 μmol/L |
| S. Uric acid | | | | | | | | M: 200-420 μmol/L<br>F: 140-340 μmol/L |
| S. ALT | | | | | | | | Up to 42 U/L |
| S. AST | | | | | | | | Up to 37 U/L |
| S. Alkaline Phosphatase | | | | | | | | Up to 117 U/L |
| Gamma GT | | | | | | | | M: ≤65 U/L<br>F: ≤45 U/L |
| S. Bilirubin | | | | | | | | 0.6 mg/dl |
| HBV DNA | | | | | | | | Quantification |
| Anti-HCV | | | | | | | | Negative |
| Anti-HIV | | | | | | | | Negative |
| Anti-HBs | | | | | | | | Up to 100,000 UI/L |
| HBsAg | | | | | | | | Positive |
| HBeAg | | | | | | | | Positive / Negative |
| Anti-HBe | | | | | | | | Positive / Negative |
| Anti-HBcAg IgM | | | | | | | | Positive / Negative |
| USG of HBS | | | | | | | | |

In addition, ALT and AST determinations will be conducted after each administration of NASVAC in the corresponding group in order to further study or explore any potential damage.

## 8. Adverse reactions

**Adverse events that can be presented and methods to register them**

General AE described for the NASVAC TVC use are generally mild **(see the Reports of Phase I Clinical Trials)**

The investigators will register the information in the CRF, describing their nature (local or systemic), intensity and the necessary treatment to relieve them.

According to WHO guidelines (WHO technical report series N° 850, 1995), the intensity degrees are:

0: No adverse reaction

1: Mild: it does not require treatment

2: Moderate: it requires treatment, and disappears with treatment.

3: Severe: it puts the patient's life in danger or produces the death. It requires prolonged hospitalization, produces significant or persistent disability or congenital malformations.

**Procedure for adverse reactions treatment**

1. In case of severe AE the treatment is stopped and the patient is immediately withdrawn from the trial.
2. In case of death, this should be immediately informed to monitors and to the main investigator of study.
3. The hospital should guarantee all the conditions and medications to handle anaphylactic reactions if they appear.
4. Another type of AE will be treated in a symptomatic form according to the rules indicated at each service.

**Notification of adverse events (AE)**

Severe AE will be notified to the main investigator and the monitors immediately or before 72 hours after apparition. If a death occurs it will be notified to the monitors before 24 hours of having happened.

Notification should include:

- General data of the patient (group of treatment, age, sex, code number)
- Date of AE, description.
- Treatment applied
- Evolution

## 9. Data management

### Procedures

| FORMS | MOMENT TO FILL | INFORMATION COLLECTED | RESPONSIBLE |
|---|---|---|---|
| Annex 1. Informed Consent | Before each patient's inclusion | Patient's, family or legal guardians consents | Responsible investigator in the service |
| Annex 2. Case Report Form and Compliance Control | At the beginning and at each evaluation | General data, clinical, laboratory, evolution and final evaluations of each patient | Specialist that evaluates |
| Annex 3A and 3B. Case Report Form for Adverse Events | After each dose | AE | Specialist that evaluates |
| Annex 4. Instructive for filling out the adverse events case report form. | Not to be filled | - | - |
| Annex 5 Scale of adverse events | Not to be filled | - | - |
| Annex 6A and 6B. Record of the medicine administration. | At each application | Medication administered | Nurse that administers or patient |

| | | | |
|---|---|---|---|
| Annex 7: Verification of inclusion and exclusion criteria before the administration of each dose | At each application | To verify criteria | Responsible investigator in the service |
| Annex 8: Model of initial screening | During the whole period of inclusion of cases | Lists of patients with diagnosis that go to the service and if they were included or not | Responsible investigator in the service |
| Annex 9 List on included volunteers | After the inclusion process | Lists of patients finally included in the trial | Responsible investigator in the service |

The clinical investigator will fill the Case Report Form (CRF) clearly and in a readable form, preferably with black ink and duplicate. The procedure appears in each CRF.

The original forms will be picked up during the visits to the institution, once complete; the duplicate will be kept by the investigator in the trial file. The primary information should be kept in the hospital during the period of time established by the authorities.

Each form should contain the subject's identification code, conformed by the inclusion number and the institution code (listing of codes appears in each CRF).

At the bottom of each model a space appears to consign the doctor's name that filled model as well as the date.

The data will be introduced in two duplicate databases by two different operators and in different computers. An application made in FOXPRO will be used. Once completed, these bases are compared in order to detect errors. The process will be repeated until no errors appear (both databases are identical).

Once be the base free of errors will be applied a program of cleaning to determine and to rectify inconsistent responses that have not been detected in the entrance of data process.

## 10. Statistics

### Statistical tests

With all the variables involved in the study, either the main variable of evaluation, and the control variables the following exploratory and diagnosis analysis will be carried out

**Exploratory analysis:**

a) Descriptive graphs will be done, depending on the type variable. With the quantitative variables (age, weight, illness duration), box-plot graphs, frequency histogram, and confidence intervals graphs for the mean; with the qualitative variables (sex, smoking habit, reaction to medications, proportion of patient with each adverse reaction), out pie graphs and frequency histograms.

b) With the qualitative variables correspondences factorial and cluster analysis will be carried out to study if there is some tendency of individuals grouping in determined categories of the variables and to infer their importance in the treatment response prediction.

c) With the quantitative variables the descriptive analysis with the estimates of the central tendency and dispersion measures that satisfy the assumptions of normal distribution: mean, mode, confidence intervals for the mean, standard deviation and maximum and minimum values. In the case of the quantitative variables that does not satisfy the assumptions of normal distribution: median, mode, minimum and maximum values, quartile range and percentiles; with the qualitative variables: the frequency histogram.

**Diagnosis:**

With the quantitative variables:

a) To study the presence of aberrant or extreme values through the following tests: t test, prove index test and 4SD test.

b) Depending on the type of analysis that is necessary from the confirmatory point of view, the specific diagnosis techniques will be applied: dispersion graphs, residuals with and without cover graphics, residuals with suppression, Cook's residuals, diagonal of the matrix Hatt parameters (leverages, (hi)).

c) In case of detecting some aberrant point through these techniques, the individual's form should be revised with that observation. If the possibility that is an entry error to the database is discarded, the following step consists on consulting with the investigators the possibility of exclusion the individual of the analysis, since it can cause biases in the results interpretation, due their deviation with the rest.

With the qualitative variables:

a) To infer the presence of extreme values of the exploratory analysis carried out (cluster analysis).

b) and c) Similar to the quantitative variables.

**Evaluation of the viral infection markers and statistic hypothesis**

**HBV-DNA:** Response: Undectectability of HBV DNA in the serum of infected patient by using a quantitative PCR assay with 250 copies/mL as a viral detection limit of sensitivity at the end of therapy with NASVAC TVC as well as in every study cut starting from dose number five and ending at one year after treatment started.

The success criteria will be assessed one year after study started however it can be evaluated for Interim Analysis according to the date of virological assessment.

***Statistical hypothesis***

It is expected that the administration of NASVAC TVC, as defined in the present protocol, to chronic hepatitis B patients, will improve the specific anti-HBV immune response of treated patients and consequently will significantly increase the percentage of patients reducing the viral load to undetectable levels after treatment completion compared to the group administered with Pegylated Interferon, expecting a difference between groups of more than 20%.

H0: Equivalency in the percentage of chronic hepatitis B patients reducing the viral load to undetectable levels. Formally, H0: $\delta \leq \delta_0 = 0.20$ where $\delta$ represents the difference between viral seronegativization, based in quantitative PCR assay with a sensitivity of 250 copies/mL, in the group treated with NASVAC compared to the group treated with Interferon.

H1: Superiority in the percentage of chronic hepatitis B patients reducing the viral load to undetectable levels. Formally, H0: $\delta > \delta_0 = 0.20$ where $\delta$ represents the difference between viral seronegativization, based in quantitative PCR assay with a sensitivity of 250 copies/mL, in the group treated with NASVAC compared to the group treated with Interferon.

**Serology: Response:**

**HBeAg:** **Negativity:** disappearance from serum

**Seroconversion:** disappearance of HBeAg from serum and appearance of HBeAb**.**

**HBsAg:** **Negativity:** disappearance from serum

**Seroconversion:** Appearance of HBsAb in serum.

Both groups will be compared according to the categories defined above by the chi-square test or odds ratio calculation. A logistic regression will be also performed for a multivariate analysis of the influence of the different control variables that result significant in the previous univariate analysis. A signification level of p = 0.1 will be considered. The potency will be of 80%.

### Evaluation of the biochemical status

**Transaminases: Response**

**ALT / AST:**

**Sustained normalization** (reduction to values under 40 IU during a period of three months time) in at least 20% compared to the initial number of patients with altered (more than 40 IU) at the beginning of treatment.

**Normalization:** Reduction to values under 40 IU after one year.

It will be studied for Interim analysis different periods of time and compared to baseline level as described in Interim analysis: after five dosis, after ten dosis, after 24 weeks (6 months) or after 56 weeks (one year)..

The ALT and AST values in serum will be compared between groups and within each group, as quantitative variables, by Student's t or the Mann-Whitney's U test, depending on the normality or not of their distributions. Paired analysis will be also performed within each group to know whether a significant change has occurred in these patients.

### Adverse events (AE)

To build a table with the frequency of AE per patient for each presented, as well as their severity, to studying which are the most frequent AE.

To study the relationship with the control variables and the appearance of each adverse reaction through a logistic regression analysis where the importance of each variable is obtained in the response prediction and the ratio of probability and conditional probabilities of response for each category of the qualitative variables and for the desired increments of the quantitative variables are estimated.

To compare both treatment groups considering the frequency of AE. For this purpose the chi-square test will be used. In case of quantitative variables that measure the intensity of a reaction, such as fever, blood pressure, ALT increase, etc. the Student's t test will be used, when possible. Otherwise, non parametric tests will be used. Paired tests will be used for the comparison between products and between periods of treatment.

### Data center:

The data processing and the statistical analysis will be performed by specialists of the Statistics Department of de Clinical Trials Division of the Center for Genetic Engineering and Biotechnology.

**Number of patients**

The decision was to take 60 patients per group, 180 patients in total, considering the statistical hypothesis.

**Interim analysis**

After 5 and 10 administrations and also at 48 weeks, patients will be tested by PCR to quantify HBV DNA in serum as well as for HBsAg and HBeAg seroconversion. A comparative study will be conducted for the same variables for internal comparisons between both groups in order to have a reference to standard treatment with a recognized efficacy.

a) A 20% superior percentage of viral load reduction to undetectable levels in the group treated with NASVAC compared to PegIFN treated group in any of the virological study cuts will be considered as a success criteria for the Interim Analysis.

b) A superior difference in the proportion of patients with ALT normalization or combined ALT normalization and viral load reduction to undetectable levels at a defined time (5, 10 dosis or 48 weeks) between groups will be considered a success criteria for the Interim Analysis.

e) A sustained biochemical (ALT) response during three consecutive determinations after dose number 10 will be considered a success criteria.

**General schedule**

| **Period** | **Beginning** | **Termination** |
|---|---|---|
| Preparation of the protocol and its amendments | Sep / 2009 | Oct / 09 |
| Recruitment and treatment of patients | Oct / 2010 | Jun / 2011 |
| Management and processing of data | Oct / 2010 | Dec / 2012 |
| Interim analysis | May / 2011 | Jun / 2011 |
| Final analysis | Dec / 2012 | March / 2013 |

*Flexibility will be accepted in schedule on the basis of recommendation of Principal investigator.*

## 11. Practicalities

**Detailed plan. Subject management**

Patients from the Gastroenterology outpatient consultation potentially can participate in the protocol. These sessions should take place at least once per week and the following steps will be taken with every patient that fulfills the inclusion criteria:

- Clinical examination by specialists.
- Laboratory analyses
- If the patient fulfills the rest of the inclusion criteria and does not have any of the exclusion criteria, his written consent is requested after the proper explanation.
- The individual data form will be filled.
- The patient will receive an explanation about the treatment and its time of duration.
- The day before the beginning of treatment, serum samples will be taken for the baseline data.
- The injections will take place every fortnight at the clinic. Clinical examinations will be done according to the established schedule.
- At each evaluative consultation the appointment for the next one will be established.

For the purpose of quantitative HBV-DNA determinations, the serum samples will be taken at the consultations and kept frozen until their transportation to laboratory. HBV-DNA determination for inclusion will be the same method to the one to be used during assay.

**Distribution of duties and responsibilities among the participants in the investigation**

**Duties of the sponsor (CIGB)**

1. To promote the clinical trial at Bangladesh.
2. To offer information about the product to the investigators including chemical-pharmaceutics, toxicological and clinical properties of the preparation of the NASVAC TVC produced at the CIGB.

3. To carry out the initiation workshop or meeting or e. mail conversation among different participants of this trial..

4. To comply with regulatory requirements regarding the study (authorization, notification of adverse events) at Bangladesh.

5. To designate the monitor that will be responsible for the study, as well as the main and participant investigators.

6. To provide the products to be used in the treatment with the quality certificates corresponding to each lot.

7. To carry out audits with the planned periodicity.

8. To keep all the primary information and the reports generated in the study for at least 15 years.

9. To maintain the information of the study conduction updated.

10. To participate in the evaluation of the results and in the elaboration of the final report.

11. To consider quickly, together with the investigators, any severe adverse events that occur, determining the procedure in each case.

**Duties of the monitors**

1. To participate in the design and elaboration of the protocol of the investigation.

2. To carry out audit and control visits in order to monitor the performance of the trial.

3. To elaborate databases, and management of the information and statistical analysis.

4. To participate in the elaboration of reports of the investigation.

5. To notify the results of the study to the Regulatory Authorities for their inclusion in the registration file of the product.

**Duties of the Principal investigators**

1. To participate in the design and elaboration of the protocol of the investigation.

2. To perform a meeting with all the participants in the investigation to explain them their duties and responsibilities.

3. To give the necessary explanations to the subjects and to obtain their informed signed consent.

4. To keep a file with the whole documentation related with the investigation, including all communications with the sponsor, monitors, and the rest of the investigators; besides the copy of the signed protocol and all the modifications that take place, and other documents. This file will be available to the monitors and quality controllers that carry out the control visits.

5. To participate in the data analysis.

6. To elaborate any partial and final reports of the investigation.

**Duties of rest of the investigators**

1. To participate in the meeting that is made to explain the execution of the investigation where they will know their responsibilities.

2. To help principal investigator to include all the patients who fulfill the inclusion criteria.

3. To write down all the information carefully in the CRF, completing all that settled down in the protocol.

4. To maintain a file with the whole documentation related with the investigation, including all the communications with the sponsor, monitors, and with the main investigator, copies of the signed protocol and all modifications that take place, model of included and not included patients, copies of the CRF models of patients, constancy of reception and delivery of the medications and other documents. This file will be available to the monitors and controllers of the quality that carry out the control visits.

5. To participate in the discussion and analysis of the investigation results.

6. To provide the medical attention of the patients those abandon the trial or refuse to participate.

7. To maintain confidentiality of the data included in the trial.

8. To participate in the quality assurance activities that is requested.

9. To control strictly the patient’s compliance to treatment by means of the treatment compliance card or by other means.

10. To detect and report to the CIGB and the Ethics Committee of the Hospital, the serious and very serious adverse events those occur.

11. To participate in the final evaluation of the results and in the elaboration of the final report.

**Instructions to the personnel**

A meeting will be made with the whole staff participating in the study to explain the duties and responsibilities of each one and to clarify all the doubts that might exist.

**Addresses and telephone numbers of the main investigator**

| Name | Address, Telephone, FAX, Email |
|---|---|
| Dr. Mamun-Al-Mahtab | House No. 109, Road No. 4 Block. B, Banani<br>Dhaka-1213, Bangladesh Tel: +880-2-8850371<br>Mobile: +880-1711567275 Fax: +880-2-8826840<br>Alternate Email <hbd@dhaka.net> |

**Considerations about confidentiality and disclosure of the results**

1. The confidentiality of the personal data of subjects will be guaranteed.

2. The disclosure of any information generated in this study is prohibited without the consent of the sponsors and principal investigators. The investigators will be able to use the results of this study for publications and/or oral presentations or in posters in scientific events but they will not be able to do it without previous agreement between CIGB and Bangladesh Liver Foundation/Labaid Dhaka Hospital of the purpose and subject matter of these publications.

3. Sponsors and investigators will carry out the analysis of the results and their interpretation together.

**Quality control and audits**

Monitoring will be done at regular intervals, depending on the progress of the investigation. The objective of the control is to check compliance with Good Clinical Practices (GCP) guidelines, demanded by sponsor. These visits will be serving to discuss any aspect of the protocol that the investigator suggests.

The investigator and the hospital unit where he belongs will permit that the monitors (properly authorized by CIGB / Labaid Dhaka hospital) audit and review the documents of the investigation and the use of the given resources. The Case Report Form models of patients will be verified, which permits a comparison with the original clinical histories.

An initiation visit will be carried out. It is foreseen to carry out monitoring and control of the quality visits to each investigation place.

# 12. Assurance

### Necessary resources

| Resources for 80 patients | Amount per patient | Total | Responsible |
|---|---|---|---|
| NASVAC | 30 bb 500mL | 3000 bb of NASVAC | CIGB |
| IFN alpha 2b | 48 bb considering 48 w | 4 000 bb considering 48 w | CIGB |
| HBV DNA quantitative* | 4 | 700 tests | Labaid Dhaka and Hospital and Faculty of Medicine |
| Anti-HCV, and anti-HIV* | 2 (each) | 360 tests | |
| Liver function and hematology tests* | 5 (each) | 900 test | |
| ALT, AST* | 8 (each) | 1440 test | |
| HBV serology * | 5 (each) | 900 test | |

(*) A number of tests will be used before definitive recruiting (before day 0).

# 13. References


1. Pentón E, Muzio V, González-Griego M. The Hepatitis B Virus (HBV) infection and its prevention by a recombinant-DNA viral surface antigen (rec- HBsAg) vaccine, Biotecnología Aplicada 1994;11:1-11.
2. Juliao, O., González, A., Ramírez, V., Rojas, M.C. González, M. (1991). Inmunogenicidad de dos vacunas recombinantes anti hepatitis B aplicando dos esquemas de inmunización. Biomedica Vol 11 No. 1-4 pp. 7.
3. Method for the obtaiment of vaccine preparates that contain particulate antigen aggregates. OCPI patent application No. 2000-279, 2000.
4. Tleugabulova D, Falcón V, Pentón E. Physico-chemical characterization of recombinant hepatitis B surface antigen by multidimensional approach. J Chromatography A, 845 (1999) 171-179.
5. Penna A, Chisari FV, Bertoletti A, Missale G, Fowler P, Giuberti T, Fiaccadori F et al. Cytotoxic T lymphocytes recognize an HLA A2 restricted epitope within the the hepatitis B virus nucleocapsid antigen. J Exp Med 1991; 174: 1565-1570.
6. Moriyama T, Guilhot S, Klopchin K, Moss B, Pinkert CA, Palmiter RD, Brinster RL, et al. Immunobiology and pathogenesis of hepatocellular injury in hepatitis B transgenic mice. Science 1990; 248, 361-364.
7. Ferrari C, Penna A, Giuberti T, Tong MJ, Ribera E, Fiaccadori F, Chisari FV. Intrahepatic nucleocapside antigen-specific T-cells in chronic active hepatitis B. J Immunol 1987; 139: 2050-2058.
8. Hoofnagle JH, Peters M, Mullen KD, Jones DB, Rustgi V, Di Bisceglie A, Hallahan C, et al. Randomized controlled trials of recombinant human $\alpha$-interferon in patients with chronic hepatitis B. Gastroenterology 1998; 95: 1318-1325.
9. Chisari FV, Ferrari C. Hepatitis B virus immunopathogenesis. Ann Rev Immunol 1995; 13: 29-60.
10. Pol S, Couillin I, Michel ML, Driss F, Nalpas B, Carnot F, Berthelot P, et al. Immunotherapy of chronic hepatitis B by anti HBV vaccine. Acta Gastroenterol Belg 1998; 61: 228-233.
11. Heathcote J, McHutchison J, Benner K, Wright T, Minuk J, Sacks S, et al. CY-1899: a therapeutic vaccine for chronic hepatitis B (Abstract) Hepatology. 1998; 28: 588A.
12. Mancini M, Hadchouel M, Davis HL, Whalen RG, Tiollais P, Michel ML. DNA mediated immunization in a transgenic mouse model of the hepatitis B surface antigen chronic carrier state. Proc Natl Acad Sci USA 1996; 93: 12496-501.
13. Livingstone BD, Alexander J, Crimi C, Oseroff C, Celis E, Kieran D, Guidotti LG, Chisari FV, Fikes J, Chesnut RW, Sette A. Altered helper T lymphocyte function associated with chronic hepatitis B virus infection and its role in response to therapeutic vaccination in humans. J Immunol, 1999, 162: 3088-3095.
14. Soussan P, Pol S, Garreau F, Brechot C, Kremsdorf D. Vaccination of chronic hepatitis B virus carriers with preS2/S envelope protein is not associated with the emergence of envelope escape mutants. J Gen Virol 2001 Feb;82(Pt 2):367-371.
15. Hepagene Effective In Difficult-To-Treat Hepatitis B Patients URL: http://www.pslgroup.com/dg/1592C2.htm
16. Bohm W, Schirmbeck R, Elbe A, Melber K, Diminky D, Kraal G, van Rooijen N, et al. Exogenous hepatitis B surface antigen particles processed by dendritic cells or macrophages prime murine MHC class I-restricted cytotoxic T lymphocytes in vitro. J Immunol 1995; 155: 3313-3321.
17. Rahman F, Dahmen A, Herzoug-Hauff S, Bocher WO, Galle PR, Lohr HF. Cellular and humoral immune responses induced by intradermal or intramuscular vaccination with the major Hepatitis B Surface Antigen. Hepatology 2000; 31: 521-527.
18. Lobaina Y, García D, Abreu N, Muzio V, Aguilar JC. Mucosal Immunogenicity of hepatitis B core antigen. Biochem. Biophys Res Commun. 2003; 300: 745-750.
19. Aguilar JC, Lobaina Y, Muzio V, Garcia D, Penton E, Iglesias E, Pichardo D, Urquiza D, Rodriguez D, Silva D, Petrovsky N, Guillen G. Development of a nasal vaccine for chronic hepatitis B infection that uses the ability of hepatitis B core antigen to stimulate a strong Th1 response against hepatitis B surface antigen. Immunology and Cell Biology 2004 Oct;82(5):539-46.
20. Schirmbeck R, Wild J, Reimann J. Similar as well as distinct MHC class I-binding peptides are generated by exogenous and endogenous processing of hepatitis B virus surface antigen. Eur.J. Immunol. 1998; 028: 4149–61.
21. Tan WS, Dyson MR, Murray K. Hepatitis B virus core antigen: enhancement of its production in Escherichia coli, and interaction of the core particles with the viral surface antigen. Biol. Chem. 2003; 384: 363–71.
22. Jiang HL, Park IK, Shin NR, Yoo HS, Akaike T, Cho CS. Controlled release of Bordetella Bronchiseptica Dermonecrotoxin (BBD) vaccine from BBD-loaded chitosan microspheres in vitro. Arch. Pharm. Res. 2004; 27:

346–50.
23. Chattaraj SC, Das SK. Physicochemical characterization of influenza viral vaccine loaded surfactant vesicles. Drug Deliv. 2003; 10: 73–7.
24. He Q, Mitchell A, Morcol T, Bell SJ. Calcium phosphate nanoparticles induce mucosal immunity and protection against herpes simplex virus type 2. Clin. Diagn. Lab. Immunol. 2002; 9: 1021–4.
25. Milich DR, Chen M, Schodel F, Peterson DL, Jones JE, Hughes JL. Role of B cells in antigen presentation of the hepatitis B core. Proc. Natl Acad. Sci. USA 1997; 94: 14 648–53.
26. Zhevachevsky NG, Nomokonova NY, Beklemishev AB, Belov GF. Dynamic study of HBsAg and HBeAg in saliva samples from patients with hepatitis B infection: diagnostic and epidemiological significance. J. Med. Virol. 2000; 61: 433–8.
27. Weiner HL. Oral Tolerance: immune mechanisms and treatment of autoimmune diseases. Immunol. Today 1997; 18: 335–41.
28. Boni C, Bertoletti A, Penna A et al. Lamivudine treatment can restore T cell responsiveness in chronic hepatitis B. J. Clin. Invest. 1998; 102: 968–75.
29. Boni C, Penna A, Ogg GS et al. Lamivudine treatment can overcome cytotoxic T-cell hyporesponsiveness in chronic hepatitis B: new perspectives for immune therapy. Hepatology 2001; 33: 963–71.
30. Lazdina U, Alheim M, Nystrom J et al. Priming of cytotoxic T cell responses to exogenous hepatitis B virus core antigen is B cell dependent. J. General Virol. 2003; 84: 139–46.
31. Maini MK, Boni C, Lee CK, Larrubia JR, Reignat S, Ogg GS, King AS, Herberg J, Gilson R, Alisa A, Williams R, Vergani D, Naoumov NV, Ferrari C, Bertoletti A. The role of virus-specific CD8(+) cells in liver damage and viral control during persistent hepatitis B virus infection. J Exp Med. 2000 Apr 17;191(8):1269-80.
32. McClary H, Koch R, Chisari FV, Guidotti LG. Relative sensitivity of hepatitis B virus and other hepatotropic viruses to the antiviral effects of cytokines. J Virol. 2000 Mar;74(5):2255-64.
33. Wieland SF, Guidotti LG, Chisari FV. Intrahepatic induction of alpha/beta interferon eliminates viral RNA-containing capsids in hepatitis B virus transgenic mice. J Virol. 2000 May;74(9):4165-73.
34. Ikeda T, Lever AM, Thomas HC. Evidence for a deficiency of interferon production in patients with chronic hepatitis B virus infection acquired in adult life. Hepatology. 1986 Sep-Oct;6(5):962-5.
35. Jung MC, Hartmann B, Gerlach JT, Diepolder H, Gruber R, Schraut W, Gruner N, Zachoval R, Hoffmann R, Santantonio T, Wachtler M, Pape GR. Virus-specific lymphokine production differs quantitatively but not qualitatively in acute and chronic hepatitis B infection. Virology 1999; 261: 165-72.
36. Nouri-Aria et al., 1991;
37. Penna A, Del Prete G, Cavalli A, Bertoletti A, D'Elios MM, Sorrentino R. Predominant T-helper 1 cytokine profile of hepatitis B virus nucleocapsid-specific T cells in acute self-limited hepatitis B. Hepatology 1997; 25: 1022-27.
38 Rehermann B, Lau D, Hoofnagle JH, Chisari FV. Cytotoxic T lymphocyte responsiveness after resolution of chronic hepatitis B virus infection. J Clin Invest 1996; 97: 1655-65.
39. Boni C, Penna A, Bertoletti A, Lamonaca V, Rapti I, Missale G. Transient restoration of anti-viral T cell responses induced by lamivudine therapy in chronic hepatitis B. J Hepatol 2003; 39: 595-605.
40. Lau GK, Suri D, Liang R, Rigopoulou EI, Thomas MG, Mullerova I. Resolution of chronic hepatitis B and anti-HBs seroconversion in humans by adoptive transfer of immunity to hepatitis B core antigen. Gastroenterology 2002; 122: 614-24.
41. Shouval D, Ilan Y. Transplantation of hepatitis B immune lymphocytes as means for adoptive transfer of immunity to hepatitis B virus. J Hepatol 1995; 23: 98-101.
