# Supplementary material for: Treatment of chronic hepatitis B naïve patients with a therapeutic vaccine containing HBs and HBc antigens (a randomized, open and treatment controlled phase III clinical trial)
Source: PLoS One. 2018 Aug 22;13(8):e0201236. doi: 10.1371/journal.pone.0201236 (PMC6104936; doi:10.1371/journal.pone.0201236)
Supplement: S1 Table — The file contains the results of Virological and serological evaluations of the timepoint assessments included in the publication. (DOC) [file pone.0201236.s004.doc]

**S1 Table.** **ANNEX 1 Virology HBs HBeAg Serology.doc**

**The file contains the results of Virological and serological evaluations of the timepoint assessments included in the publication**.

Annex 1.1.A. Virology results (Viral load [copies/mL]) of chronic hepatitis B patients treated with PegIFN.

Annex 1.1.B. Virology results (Viral load [copies/mL]) of chronic hepatitis B patients treated with NASVAC.

Annex 1.2.A. PegIFN treated patients: Serology results (qualitative HBsAg).

Annex 1.2.B. NASVAC treated patients: Serology results (qualitative HBsAg).

Annex 1.3.A. Serology results (qualitative HBeAg & anti HBeAg determination) of chronic hepatitis B patients treated with PegIFN.

Annex 1.3.B. Serology results (qualitative HBeAg & anti HBeAg determination) of chronic hepatitis B patients treated with NASVAC.

Annex 3.1.A. Virology results (Viral load [copies/mL]) of chronic hepatitis B patients treated with PegIFN. Lower limit of detection: 250 copies/mL. Negative (neg) values <250 copies/mL; ND: not done. W24: NASVAC end of treatment; W48: PegIFN end of treatment.

| I.N. | Baseline | W12 | W24 | W48 | W76 |
| --- | --- | --- | --- | --- | --- |
| 2 | 2,50E+04 | neg | neg | neg | 7,75E+02 |
| 4 | 1,40E+06 | neg | neg | neg | 1,21E+03 |
| 6 | 1,70E+04 | neg | neg | neg | 3,60E+02 |
| 9 | 1,35E+03 | neg | neg | neg | neg |
| 12 | 1,40E+05 | neg | 6,45E+03 | 7,15E+02 | 1,36E+02 |
| 13 | 2,20E+04 | neg | neg | neg | neg |
| 15 | 2,50E+04 | neg | ND | 7,90E+02 | 6,10E+02 |
| 16 | 5,70E+05 | DROPPED | DROPPED | DROPPED | DROPPED |
| 17 | 2,00E+04 | neg | neg | neg | neg |
| 19 | 4,40E+03 | neg | ND | neg | 1,17E+03 |
| 21 | 8,10E+03 | neg | neg | neg | neg |
| 22 | 1,20E+04 | neg | neg | neg | neg |
| 26 | 3,17E+03 | neg | 1,50E+01 | neg | 5,60E+03 |
| 28 | 3,06E+07 | neg | neg | neg | 2,45E+02 |
| 31 | 2,30E+05 | neg | neg | neg | 1,67E+03 |
| 33 | 1,25E+03 | neg | neg | neg | ND |
| 34 | 1,50E+04 | neg | neg | neg | 1,04E+04 |
| 35 | 2,40E+06 | neg | 9,75E+02 | neg | 5,35E+03 |
| 36 | 5,00E+06 | 8,70E+04 | neg | 1,53E+02 | 7,80E+02 |
| 37 | 3,40E+06 | 2,95E+02 | neg | neg | 1,65E+05 |
| 41 | 1,93E+06 | neg | neg | neg | 3,00E+04 |
| 42 | 2,70E+04 | neg | neg | neg | neg |
| 43 | 1,10E+05 | 8,00E+00 | 7,05E+02 | neg | 3,73E+04 |
| 44 | 1,98E+04 | 1,42E+05 | 2,24E+01 | neg | 3,29E+05 |
| 46 | 1,90E+07 | 8,35E+02 | neg | neg | neg |
| 47 | 7,40E+03 | neg | neg | neg | neg |
| 51 | 1,10E+04 | neg | neg | 3,48E+04 | ND |
| 52 | 1,33E+05 | 1,23E+05 | neg | neg | neg |
| 53 | 8,00E+03 | DROPPED | DROPPED | DROPPED | DROPPED |
| 55 | 8,20E+03 | neg | neg | ND | 1,89E+02 |
| 61 | 2,88E+05 | neg | 4,31E+01 | neg | 4,72E+04 |
| 62 | 5,80E+03 | neg | neg | neg | ND |
| 63 | 9,20E+03 | neg | neg | neg | neg |
| 64 | 9,35E+04 | 2,97E+02 | 2,05E+03 | neg | neg |
| 65 | 1,53E+03 | neg | neg | ND | 6,25E+04 |
| 66 | 1,90E+10 | 6,05E+05 | 1,80E+01 | neg | 2,13E+07 |
| 70 | 3,00E+05 | neg | 8,10E+01 | 3,81E+09 | 7,55E+08 |
| 71 | 2,70E+03 | neg | neg | neg | 2,40E+04 |
| 73 | 5,09E+09 | 2,29E+04 | 1,66E+04 | 3,52E+05 | 2,24E+08 |
| 74 | 4,59E+03 | neg | neg | ND | neg |

Annex 3.1.A. Virology results (Viral load [copies/mL]) of chronic hepatitis B patients treated with PegIFN, 2^nd^ part.

| I.N. | Baseline | W12 | W24 | W48 | W76 |
| --- | --- | --- | --- | --- | --- |
| 81 | 2,80E+04 | neg | neg | neg | neg |
| 83 | 2,80E+05 | 4,31E+03 | neg | neg | neg |
| 86 | 2,24E+03 | neg | neg | neg | neg |
| 87 | 3,90E+06 | 1,42E+05 | 2,45E+04 | 5,60E+03 | 1,95E+08 |
| 93 | 3,50E+12 | 7,30E+06 | ND | ND | 9,45E+07 |
| 94 | 1,90E+04 | neg | neg | ND | 1,46E+06 |
| 97 | 5,50E+07 | neg | 1,05E+02 | neg | 3,50E+02 |
| 98 | 2,86E+07 | neg | neg | ND | neg |
| 99 | 1,50E+05 | neg | neg | neg | neg |
| 100 | 2,90E+04 | 9,00E+00 | neg | neg | 6,75E+02 |
| 102 | 2,42E+04 | 1,60E+01 | neg | ND | 2,59E+04 |
| 104 | 6,90E+11 | 3,13E+05 | 8,20E+05 | DROPPED | DROPPED |
| 105 | 1,00E+10 | 7,80E+04 | 5,35E+03 | 2,34E+04 | 6,15E+05 |
| 107 | 3,90E+05 | neg | neg | ND | 1,44E+04 |
| 109 | 1,00E+06 | DROPPED | DROPPED | DROPPED | DROPPED |
| 111 | 2,60E+06 | neg | neg | neg | 1,05E+05 |
| 114 | 1,13E+08 | 1,70E+08 | 6,55E+08 | 3,17E+08 | 2,31E+08 |
| 115 | 1,10E+05 | neg | neg | neg | 1,37E+04 |
| 117 | 3,90E+08 | 2,02E+02 | 6,00E+00 | 2,82E+03 | 7,25E+06 |
| 118 | 2,50E+06 | 3,40E+03 | 1,27E+04 | 8,38E+01 | 7,85E+05 |
| 121 | 2,19E+07 | 2,19E+05 | 1.68x10^4^ | 2,19E+03 | 8,15E+06 |
| 122 | 8,70E+07 | 1,38E+03 | 1.32x10^3^ | 3,73E+06 | 1,76E+07 |
| 124 | 3,20E+03 | neg | neg | neg | neg |
| 128 | 5,90E+10 | neg | neg | neg | 2,54E+07 |
| 130 | 5,60E+06 | 1,70E+06 | 8,35E+03 | 1,31E+05 | 2,67E+08 |
| 132 | 2,20E+05 | neg | neg | neg | 4,17E+02 |
| 134 | 8,00E+05 | neg | neg | neg | neg |
| 136 | 1,70E+04 | neg | neg | neg | 3,90E+05 |
| 138 | 9,20E+03 | neg | neg | neg | neg |
| 139 | 7,10E+09 | 2,56E+06 | 5,65E+06 | 6,20E+05 | 2,00E+09 |
| 141 | 1,90E+05 | neg | neg | neg | neg |
| 143 | 1,32E+07 | 9,90E+02 | 3,93E+03 | ND | 6,20E+07 |
| 144 | 1,21E+03 | neg | neg | neg | neg |
| 145 | 1,80E+05 | neg | 3,80E+01 | neg | neg |
| 146 | 3,60E+03 | 1,18E+02 | 2,11E+02 | neg | 3,45E+02 |
| 147 | 7,10E+03 | neg | neg | ND | neg |
| 148 | 3,50E+12 | 3,60E+06 | 1,75E+05 | 1,80E+04 | 5,90E+06 |
| 154 | 1,30E+05 | 1,56E+05 | neg | ND | 1,77E+08 |
| 155 | 1,90E+05 | neg | neg | neg | neg |
| 156 | 1,70E+12 | 9,25E+05 | 1,21E+07 | 4,83E+05 | 9,13E+08 |

Annex 3.1.B. Virology results (Viral load [copies/mL]) of chronic hepatitis B patients treated with NASVAC.

| I.N. | Baseline | W12 | W24 | W48 | W76 |
| --- | --- | --- | --- | --- | --- |
| 1 | 2,70E+03 | neg | neg | neg | 7,35E+02 |
| 3 | 3,70E+03 | neg | neg | neg | 9,60E+02 |
| 5 | 3,30E+04 | neg | neg | neg | 4,82E+03 |
| 7 | 5,20E+04 | 7,95E+03 | neg | 6,60E+01 | 6,00E+03 |
| 8 | 2,20E+04 | neg | neg | 3,15E+02 | 3,85E+02 |
| 10 | 3,30E+04 | neg | 5,50E+02 | neg | 2,19E+03 |
| 11 | 6,85E+04 | neg | 6,25E+02 | 7,25E+03 | 2,64E+03 |
| 14 | 4,30E+04 | neg | neg | neg | 2,64E+06 |
| 18 | 1,50E+09 | 5,40E+04 | 1,48E+04 | 7,20E+02 | neg |
| 20 | 6,30E+04 | neg | neg | 5,91E+01 | 1,60E+06 |
| 23 | 8,40E+03 | neg | neg | neg | ND |
| 24 | 1,10E+06 | 6,10E+01 | neg | neg | 3,59E+03 |
| 25 | 2,70E+03 | neg | ND | neg | neg |
| 27 | 6,20E+03 | neg | neg | 1,57E+02 | 1,18E+03 |
| 29 | 9,30E+03 | 3,45E+02 | 3,90E+01 | 3,32E+01 | 8,75E+02 |
| 30 | 4,90E+04 | neg | neg | 7,50E+02 | neg |
| 32 | 3,00E+03 | neg | 2,05E+03 | neg | neg |
| 38 | 1,20E+05 | neg | neg | neg | 9,60E+02 |
| 39 | 2,11E+07 | DROPPED | DROPPED | DROPPED | DROPPED |
| 40 | 1,90E+03 | neg | 2,83E+03 | 7,55E+02 | 5,40E+02 |
| 45 | 2,40E+09 | 2,09E+04 | 2,82E+03 | 1,19E+06 | 9,65E+05 |
| 48 | 1,60E+04 | neg | neg | neg | 3,74E+03 |
| 49 | 4,53E+03 | 1,15E+02 | 1,25E+04 | neg | 8,25E+03 |
| 50 | 7,20E+03 | neg | 4,02E+02 | 3,68E+02 | 1,90E+02 |
| 54 | 5,90E+03 | neg | 4,20E+01 | 4,20E+01 | ND |
| 56 | 6,70E+04 | neg | neg | neg | neg |
| 57 | 1,40E+12 | 5,40E+07 | 3,85E+06 | 3,35E+07 | 1,51E+07 |
| 58 | 6,50E+07 | 6,05E+03 | 2,30E+01 | 1,60E+04 | ND |
| 59 | 5,00E+08 | 3,17E+04 | 1,72E+02 | 1,02E+03 | 3,57E+04 |
| 60 | NP | NP | NP | NP | NP |
| 67 | 1,90E+04 | ND | neg | neg | ND |
| 68 | 3,70E+05 | 2,11E+03 | 9,35E+02 | 1,40E+02 | 1,81E+02 |
| 69 | NP | NP | NP | NP | NP |
| 72 | 2,70E+04 | 13 | neg | neg | neg |
| 75 | 9,30E+05 | 148 | neg | 2,38E+03 | neg |
| 76 | 5,50E+04 | 280 | neg | neg | 6,35E+03 |
| 77 | 4,33E+03 | neg | neg | neg | ND |
| 78 | 6,00E+03 | 7,55E+04 | 9,55E+04 | 6,65E+01 | 1,37E+02 |
| 79 | 3,50E+04 | neg | neg | 3,09E+02 | 1,22E+03 |
| 80 | 3,00E+06 | ND | ND | neg | 6,82E+02 |

Annex 3.1.B. Virology results (Viral load [copies/mL]) of chronic hepatitis B patients treated with NASVAC, 2^nd^ part.

| I.N. | Baseline | W12 | W24 | W48 | W76 |
| --- | --- | --- | --- | --- | --- |
| 82 | 4,20E+05 | 3,39E+03 | neg | 5,85E+03 | 3,59E+03 |
| 84 | 2,50E+04 | neg | neg | neg | neg |
| 85 | 3,80E+04 | 3,48E+02 | neg | 3,02E+02 | ND |
| 88 | 1,00E+05 | neg | 1,84E+03 | neg | neg |
| 89 | 1,02E+05 | 5,45E+02 | 2,30E+01 | 6,10E+02 | 1,23E+05 |
| 90 | 5,70E+06 | 1,46E+05 | 1,19E+03 | 1,33E+05 | 6,45E+04 |
| 91 | 1,80E+07 | 6,65E+03 | 5,00E+04 | ND | 3,46E+03 |
| 92 | 7,95E+04 | neg | 3,55E+02 | 3,31E+01 | neg |
| 95 | 6,90E+03 | neg | neg | 5,75E+00 | 1,49E+03 |
| 96 | 2,49E+05 | 3,20E+05 | 3,39E+04 | 1,31E+02 | 3,80E+04 |
| 101 | 1,50E+05 | 7,90E+02 | 8,80E+02 | 5,60E+03 | ND |
| 103 | 6,00E+11 | 6,95E+05 | 4,46E+05 | 4,17E+05 | 3,28E+06 |
| 106 | 5,10E+05 | neg | neg | 1,76E+04 | 5,50E+03 |
| 108 | 1,62E+07 | 1,26E+06 | 2,80E+06 | 1,12E+04 | 3,28E+03 |
| 110 | 5,30E+05 | 1,66E+04 | 1,13E+04 | 1,17E+05 | 1,30E+07 |
| 112 | 1,60E+04 | neg | ND | ND | ND |
| 113 | 2,70E+03 | neg | 3,96E+02 | 2,17E+02 | 1,74E+02 |
| 116 | 9,40E+05 | 198 | neg | 7,50E+00 | 3,26E+03 |
| 119 | 3,50E+12 | 1,71E+07 | neg | 2,69E+07 | ND |
| 120 | 5,85E+07 | 1,79E+03 | 1,90E+02 | 8,11E+01 | 1,56E+04 |
| 123 | 1,50E+06 | 1,68E+04 | 9,65E+03 | 2,48E+05 | 1,23E+04 |
| 125 | 1,70E+03 | neg | neg | neg | neg |
| 126 | 1,80E+05 | 4,26E+02 | neg | 1,75E+02 | 4,22E+03 |
| 127 | 2,30E+04 | neg | neg | neg | neg |
| 129 | 1,60E+05 | 1,07E+04 | 5,80E+04 | neg | ND |
| 131 | 1,70E+08 | DROPPED | DROPPED | DROPPED | DROPPED |
| 133 | 1,00E+13 | 3,03E+05 | 8,50E+01 | 1,73E+06 | 1,51E+06 |
| 135 | 3,99E+04 | 1,39E+05 | 2,12E+03 | 3,68E+03 | 3,51E+03 |
| 137 | 9,70E+03 | 5,50E+01 | neg | neg | 8,80E+02 |
| 140 | 6,05E+04 | 5,20E+02 | 6,00E+00 | 5,35E+02 | 1,20E+02 |
| 142 | 3,50E+04 | 1,43E+03 | 7,95E+02 | 6,30E+02 | ND |
| 149 | 1,00E+04 | neg | neg | neg | 2,32E+03 |
| 150 | 2,40E+04 | neg | 2,82E+03 | neg | ND |
| 151 | 4,10E+04 | neg | neg | neg | 1,58E+04 |
| 152 | 5,48E+05 | DROPPED | DROPPED | DROPPED | DROPPED |
| 153 | 2,60E+04 | neg | neg | neg | neg |
| 157 | 7,90E+03 | neg | 1,20E+01 | neg | neg |
| 158 | 1,10E+07 | neg | neg | 6,15E+02 | ND |
| 159 | 2,20E+04 | neg | neg | 2,21E+02 | 2,17E+03 |
| 160 | 4,80E+05 | neg | neg | 8,60E+01 | ND |

Annex 3.2.A. PegIFN treated patients: Serology results (qualitative HBsAg), Preimmune (PI) and end of follow-up (E of F-up) response.

| I.N. | HBsAg  PI | HBsAg  E of F-up |
| --- | --- | --- |
| 2 | pos | pos |
| 4 | pos | pos |
| 6 | pos | pos |
| 9 | pos | pos |
| 12 | pos | pos |
| 13 | pos | pos |
| 15 | pos | pos |
| 16 | pos | pos |
| 17 | pos | pos |
| 19 | pos | pos |
| 21 | pos | pos |
| 22 | pos | pos |
| 26 | pos | pos |
| 28 | pos | pos |
| 31 | pos | pos |
| 33 | pos | pos |
| 34 | pos | pos |
| 35 | pos | pos |
| 36 | pos | pos |
| 37 | pos | pos |
| 41 | pos | pos |
| 42 | pos | pos |
| 43 | pos | pos |
| 44 | pos | pos |
| 46 | pos | pos |
| 47 | pos | pos |
| 51 | pos | pos |
| 52 | pos | pos |
| 53 | pos | pos |
| 55 | pos | pos |
| 61 | pos | pos |
| 62 | pos | pos |
| 63 | pos | pos |
| 64 | pos | pos |
| 65 | pos | pos |
| 66 | pos | pos |
| 70 | pos | pos |
| 71 | pos | pos |
| 73 | pos | pos |
| 74 | pos | pos |

Annex 3.2.A. PegIFN treated patients: Serology results (qualitative HBsAg), Preimmune (PI) and end of follow-up (E of F-up) response.

| I.N. | HBsAg  PI | HBsAg  E of F-up |
| --- | --- | --- |
| 81 | pos | pos |
| 83 | pos | pos |
| 86 | pos | pos |
| 87 | pos | pos |
| 93 | pos | pos |
| 94 | pos | pos |
| 97 | pos | pos |
| 98 | pos | pos |
| 99 | pos | pos |
| 100 | pos | pos |
| 102 | pos | pos |
| 104 | pos | pos |
| 105 | pos | pos |
| 107 | pos | pos |
| 109 | pos | pos |
| 111 | pos | pos |
| 114 | pos | pos |
| 115 | pos | pos |
| 117 | pos | pos |
| 118 | pos | pos |
| 121 | pos | pos |
| 122 | pos | pos |
| 124 | pos | pos |
| 128 | pos | pos |
| 130 | pos | pos |
| 132 | pos | pos |
| 134 | pos | pos |
| 136 | pos | pos |
| 138 | pos | pos |
| 139 | pos | pos |
| 141 | pos | pos |
| 143 | pos | pos |
| 144 | pos | pos |
| 145 | pos | pos |
| 146 | pos | pos |
| 147 | pos | pos |
| 148 | pos | pos |
| 154 | pos | pos |
| 155 | pos | pos |
| 156 | pos | pos |

Annex 3.2.B. NASVAC treated patients: Serology results (qualitative HBsAg), Preimmune (PI) and end of follow-up (E of F-up) response.

| I.N. | HBsAg  PI | HBsAg E of F-up |
| --- | --- | --- |
| 1 | pos | pos |
| 3 | pos | pos |
| 5 | pos | pos |
| 7 | pos | pos |
| 8 | pos | pos |
| 10 | pos | pos |
| 11 | pos | pos |
| 14 | pos | pos |
| 18 | pos | pos |
| 20 | pos | pos |
| 23 | pos | pos |
| 24 | pos | pos |
| 25 | pos | pos |
| 27 | pos | pos |
| 29 | pos | pos |
| 30 | pos | pos |
| 32 | pos | pos |
| 38 | pos | pos |
| 39 | pos | pos |
| 40 | pos | pos |
| 45 | pos | pos |
| 48 | pos | pos |
| 49 | pos | pos |
| 50 | pos | pos |
| 54 | pos | pos |
| 56 | pos | pos |
| 57 | pos | pos |
| 58 | pos | pos |
| 59 | pos | pos |
| 60 | NP | NP |
| 67 | pos | pos |
| 68 | pos | pos |
| 69 | NP | NP |
| 72 | pos | pos |
| 75 | pos | pos |
| 76 | pos | pos |
| 77 | pos | pos |
| 78 | pos | pos |
| 79 | pos | pos |
| 80 | pos | pos |

Annex 3.2.B. NASVAC treated patients: Serology results (qualitative HBsAg): preimmune (PI), and end of follow-up (E of F-up) response.

| I.N. | HBsAg  PI | HBsAg E of F-up |
| --- | --- | --- |
| 82 | pos | pos |
| 84 | pos | pos |
| 85 | pos | pos |
| 88 | pos | pos |
| 89 | pos | pos |
| 90 | pos | pos |
| 91 | pos | pos |
| 92 | pos | pos |
| 95 | pos | pos |
| 96 | pos | pos |
| 101 | pos | pos |
| 103 | pos | pos |
| 106 | pos | pos |
| 108 | pos | pos |
| 110 | pos | pos |
| 112 | pos | pos |
| 113 | pos | pos |
| 116 | pos | pos |
| 119 | pos | pos |
| 120 | pos | pos |
| 123 | pos | pos |
| 125 | pos | pos |
| 126 | pos | pos |
| 127 | pos | pos |
| 129 | pos | pos |
| 131 | pos | pos |
| 133 | pos | pos |
| 135 | pos | pos |
| 137 | pos | pos |
| 140 | pos | pos |
| 142 | pos | pos |
| 149 | pos | pos |
| 150 | pos | pos |
| 151 | pos | pos |
| 152 | pos | pos |
| 153 | pos | pos |
| 157 | pos | pos |
| 158 | pos | pos |
| 159 | pos | pos |
| 160 | pos | pos |

Annex 3.3.A. Serology results (qualitative HBeAg & anti HBeAg determination) of chronic hepatitis B patients treated with PegIFN.

| I.N. | HBeAg PI | Anti-  HBe PI | HBeAg W24 | HBeAg E of F-up | Anti-HBe E of F-up |
| --- | --- | --- | --- | --- | --- |
| 2 | neg | pos | NA | NA | NA |
| 4 | neg | pos | NA | NA | NA |
| 6 | neg | pos | NA | NA | NA |
| 9 | neg | pos | NA | NA | NA |
| 12 | neg | pos | NA | NA | NA |
| 13 | neg | pos | NA | NA | NA |
| 15 | neg | pos | NA | NA | NA |
| 16 | neg | neg | DROPPED | DROPPED | DROPPED |
| 17 | neg | pos | NA | NA | NA |
| 19 | neg | pos | NA | NA | NA |
| 21 | neg | pos | NA | NA | NA |
| 22 | neg | pos | NA | NA | NA |
| 26 | neg | pos | NA | NA | NA |
| 28 | neg | pos | NA | NA | NA |
| 31 | neg | pos | NA | NA | NA |
| 33 | neg | pos | NA | NA | NA |
| 34 | neg | pos | NA | NA | NA |
| 35 | neg | pos | NA | NA | NA |
| 36 | neg | neg | NA | NA | NA |
| 37 | neg | pos | NA | NA | NA |
| 41 | neg | neg | NA | NA | NA |
| 42 | neg | pos | NA | NA | NA |
| 43 | neg | pos | NA | NA | NA |
| 44 | neg | pos | NA | NA | NA |
| 46 | neg | neg | NA | NA | NA |
| 47 | neg | pos | NA | NA | NA |
| 51 | neg | pos | NA | NA | NA |
| 52 | neg | neg | NA | NA | NA |
| 53 | neg | pos | NA | DROPPED | DROPPED |
| 55 | neg | pos | NA | NA | NA |
| 61 | neg | pos | NA | NA | NA |
| 62 | neg | neg | NA | NA | NA |
| 63 | neg | pos | NA | NA | NA |
| 64 | neg | pos | NA | NA | NA |
| 65 | neg | pos | NA | NA | NA |
| 66 | pos | neg | pos | pos | neg |
| 70 | neg | pos | NA | NA | NA |
| 71 | neg | pos | NA | NA | NA |
| 73 | pos | neg | pos | pos | neg |
| 74 | neg | neg | NA | NA | NA |

Annex 3.3.A. Serology results (qualitative HBeAg & anti HBeAg determination) of chronic hepatitis B patients treated with PegIFN, 2^nd^ part.

| I.N. | HBeAg PI | Anti-  HBe PI | HBeAg W24 | HBeAg E of F-up | Anti-HBe E of F-up |
| --- | --- | --- | --- | --- | --- |
| 81 | neg | neg | NA | NA | NA |
| 83 | neg | pos | NA | NA | NA |
| 86 | neg | pos | NA | NA | NA |
| 87 | pos | neg | pos | ND | ND |
| 93 | pos | neg | ND | ND | ND |
| 94 | neg | neg | NA | NA | NA |
| 97 | pos | neg | neg | neg | neg |
| 98 | neg | pos | NA | NA | NA |
| 99 | neg | pos | NA | NA | NA |
| 100 | neg | pos | NA | NA | NA |
| 102 | neg | pos | NA | NA | NA |
| 104 | pos | neg | DROPPED | DROPPED | DROPPED |
| 105 | neg | pos | NA | NA | NA |
| 107 | pos | neg | neg | NA | NA |
| 109 | neg | pos | DROPPED | DROPPED | DROPPED |
| 111 | pos | neg | ND | neg | pos |
| 114 | pos | neg | ND | pos | neg |
| 115 | neg | pos | NA | NA | NA |
| 117 | neg | pos | NA | NA | NA |
| 118 | pos | neg | neg | neg | pos |
| 121 | pos | neg | neg | neg | pos |
| 122 | pos | neg | neg | neg | neg |
| 124 | neg | pos | NA | NA | NA |
| 128 | pos | neg | neg | neg | neg |
| 130 | pos | neg | neg | neg | neg |
| 132 | neg | pos | NA | NA | NA |
| 134 | neg | pos | NA | NA | NA |
| 136 | neg | pos | NA | NA | NA |
| 138 | neg | pos | NA | NA | NA |
| 139 | pos | neg | pos | pos | neg |
| 141 | neg | pos | NA | NA | NA |
| 143 | neg | pos | NA | NA | NA |
| 144 | neg | pos | NA | NA | NA |
| 145 | neg | pos | NA | NA | NA |
| 146 | neg | pos | NA | NA | NA |
| 147 | neg | pos | NA | NA | NA |
| 148 | pos | neg | pos | pos | neg |
| 154 | neg | pos | NA | NA | NA |
| 155 | neg | pos | NA | NA | NA |
| 156 | pos | neg | pos | ND | neg |

Annex 3.3.B. Serology results (qualitative HBeAg & anti HBeAg determination) of chronic hepatitis B patients treated with NASVAC.

| I.N. | HBeAg PI | Anti-  HBe PI | HBeAg W24 | HBeAg E of F-up | Anti-HBe E of F-up |
| --- | --- | --- | --- | --- | --- |
| 1 | neg | pos | NA | NA | NA |
| 3 | neg | pos | NA | NA | NA |
| 5 | neg | pos | NA | NA | NA |
| 7 | neg | pos | NA | NA | NA |
| 8 | neg | pos | NA | NA | NA |
| 10 | neg | pos | NA | NA | NA |
| 11 | neg | pos | NA | NA | NA |
| 14 | neg | pos | NA | NA | NA |
| 18 | pos | neg | neg | neg | neg |
| 20 | neg | pos | NA | NA | NA |
| 23 | neg | pos | NA | NA | NA |
| 24 | pos | pos | neg | neg | pos |
| 25 | neg | pos | NA | NA | NA |
| 27 | neg | pos | NA | NA | NA |
| 29 | neg | pos | NA | NA | NA |
| 30 | neg | pos | NA | NA | NA |
| 32 | neg | pos | NA | NA | NA |
| 38 | neg | pos | NA | NA | NA |
| 39 | neg | pos | NA | NA | NA |
| 40 | neg | pos | NA | NA | NA |
| 45 | neg | pos | NA | NA | NA |
| 48 | neg | pos | NA | NA | NA |
| 49 | neg | pos | NA | NA | NA |
| 50 | neg | pos | NA | NA | NA |
| 54 | neg | pos | NA | NA | NA |
| 56 | neg | pos | NA | NA | NA |
| 57 | pos | neg | pos | pos | neg |
| 58 | neg | pos | NA | NA | NA |
| 59 | neg | pos | NA | NA | NA |
| 60 | NP | NP | NP | NP | NP |
| 67 | neg | neg | NA | NA | NA |
| 68 | neg | pos | NA | NA | NA |
| 69 | NP | NP | NP | NP | NP |
| 72 | neg | neg | NA | NA | NA |
| 75 | pos | neg | ND | neg | pos |
| 76 | neg | pos | NA | NA | NA |
| 77 | neg | pos | NA | NA | NA |
| 78 | neg | pos | NA | NA | NA |
| 79 | pos | neg | neg | neg | neg |
| 80 | neg | neg | NA | NA | NA |

Annex 3.3.B. Serology results (qualitative HBeAg & anti HBeAg determination) of chronic hepatitis B patients treated with NASVAC, 2^nd^ part.

| I.N. | HBeAg PI | Anti-  HBe PI | HBeAg W24 | HBeAg E of F-up | Anti-HBe E of F-up |
| --- | --- | --- | --- | --- | --- |
| 82 | neg | pos | NA | NA | NA |
| 84 | neg | pos | NA | NA | NA |
| 85 | neg | pos | NA | NA | NA |
| 88 | neg | pos | NA | NA | NA |
| 89 | neg | neg | NA | NA | NA |
| 90 | pos | neg | pos | pos | neg |
| 91 | neg | pos | NA | NA | NA |
| 92 | neg | pos | NA | NA | NA |
| 95 | neg | pos | NA | NA | NA |
| 96 | neg | pos | NA | NA | NA |
| 101 | neg | pos | NA | NA | NA |
| 103 | pos | neg | pos | pos | neg |
| 106 | pos | neg | neg | pos | neg |
| 108 | pos | neg | ND | neg | neg |
| 110 | pos | neg | neg | neg | pos |
| 112 | neg | NA | NA | NA | NA |
| 113 | neg | pos | NA | NA | NA |
| 116 | pos | neg | ND | neg | pos |
| 119 | pos | neg | pos | pos | neg |
| 120 | pos | neg | ND | neg | pos |
| 123 | neg | neg | NA | NA | NA |
| 125 | neg | pos | NA | NA | NA |
| 126 | neg | pos | NA | NA | NA |
| 127 | neg | pos | NA | NA | NA |
| 129 | neg | pos | NA | NA | NA |
| 131 | pos | neg | DROPPED | DROPPED | DROPPED |
| 133 | pos | neg | pos | pos | neg |
| 135 | neg | pos | NA | NA | NA |
| 137 | neg | pos | NA | NA | NA |
| 140 | neg | pos | NA | NA | NA |
| 142 | neg | pos | NA | NA | NA |
| 149 | neg | pos | NA | NA | NA |
| 150 | neg | pos | NA | NA | NA |
| 151 | neg | pos | NA | NA | NA |
| 152 | neg | pos | DROPPED | DROPPED | DROPPED |
| 153 | neg | pos | NA | NA | NA |
| 157 | neg | pos | NA | NA | NA |
| 158 | neg | pos | NA | NA | NA |
| 159 | neg | pos | NA | NA | NA |
| 160 | neg | neg | NA | NA | NA |
