# Supplementary material for: Treatment of chronic hepatitis B naïve patients with a therapeutic vaccine containing HBs and HBc antigens (a randomized, open and treatment controlled phase III clinical trial)
Source: PLoS One. 2018 Aug 22;13(8):e0201236. doi: 10.1371/journal.pone.0201236 (PMC6104936; doi:10.1371/journal.pone.0201236)
Supplement: S2 Table — The file contains the results of Biochemistry and liver function tests of the timepoint assessments included in the publication. (DOC) [file pone.0201236.s005.doc]

**S2 Table. ANNEX 2 Biochemistry and liver function tests.doc**

**The file contains the results of Biochemistry and liver function tests of the timepoint assessments included in the publication**.

Annex 2.1.A. Biochemistry results (ALT values [U/L]) of chronic hepatitis B patients treated with PEGIFN.

Annex 2.1.B. Biochemistry results (ALT values [U/L]) of chronic hepatitis B patients treated with NASVAC.

Annex 2.2.A. Biochemistry results (AST values [U/L]) of chronic hepatitis B patients treated with PEGIFN.

Annex 2.2.B. Biochemistry results (AST values [U/L]) of chronic hepatitis B patients treated with NASVAC.

Annex 2.3.A. Liver function tests (Bilirubin levels [mg/dL]) of chronic hepatitis B patients treated with PEGIFN.

Annex 2.4.B. Liver function tests (Bilirubin levels [mg/dL]) of chronic hepatitis B patients treated with NASVAC.

Annex 2.5.A. Liver function tests (Alc. phosphatase [U/L]) levels of chronic hepatitis B patients treated with PEGIFN.

Annex 2.5.B. Liver function tests (Alc. phosphatase [U/L]) levels of chronic hepatitis B patients treated with NASVAC.

Annex 2.1.A. Biochemistry results (ALT values [U/L]) of chronic hepatitis B patients treated with PEGIFN. ND: Not done. *Reference range*: M[up to 29] / F[up to 19].

| I.N. | W0 | W12 | W24 | W48 | W76 |
| --- | --- | --- | --- | --- | --- |
| 2 | 24 | 36 | 46 | 19 | ND |
| 4 | 31 | 73 | 26 | 17 | 18 |
| 6 | 29 | 35 | 32 | 24 | 54 |
| 9 | 17 | 36 | 48 | 30 | 18 |
| 12 | 20 | 170 | 62 | 52 | 19 |
| 13 | 35 | 66 | 43 | 36 | 45 |
| 15 | 21 | 52 | 125 | 72 | ND |
| 16 | 10 | DROPPED | DROPPED | DROPPED | DROPPED |
| 17 | 21 | 76 | 53 | 39 | 24 |
| 19 | 41 | 20 | 20 | 43 | ND |
| 21 | 47 | 46 | 42 | 23 | 37 |
| 22 | 87 | 93 | 28 | 38 | 52 |
| 26 | 26 | 49 | 33 | 27 | 23 |
| 28 | 37 | 64 | 62 | 72 | 34 |
| 31 | 90 | 47 | 44 | 30 | 45 |
| 33 | 56 | 100 | 22 | 20 | 57 |
| 34 | 63 | 132 | 65 | 48 | ND |
| 35 | 31 | 37 | 83 | 43 | 26 |
| 36 | 50 | 62 | 35 | 31 | 39 |
| 37 | 108 | 106 | 121 | 100 | 99 |
| 41 | 30 | 35 | 32 | 40 | ND |
| 42 | 43 | 93 | 60 | 31 | 34 |
| 43 | 35 | 72 | 94 | 93 | 49 |
| 44 | 169 | 99 | 66 | 35 | 82 |
| 46 | 29 | 23 | 49 | 34 | 32 |
| 47 | 24 | 55 | 23 | 19 | 23 |
| 51 | 31 | 37 | 44 | 85 | ND |
| 52 | 43 | 129 | 149 | 62 | 33 |
| 53 | 19 | 24 | DROPPED | DROPPED | DROPPED |
| 55 | 28 | 70 | 82 | ND | 22 |
| 61 | 30 | 196 | 44 | 30 | 17 |
| 62 | 23 | 39 | 85 | 81 | ND |
| 63 | 41 | 45 | 40 | 27 | 28 |
| 64 | 47 | 57 | 69 | 46 | 29 |
| 65 | 44 | 31 | 38 | ND | 18 |
| 66 | 61 | 27 | 30 | 34 | 67 |
| 70 | 27 | 33 | 26 | 31 | 40 |
| 71 | 42 | 44 | 42 | 36 | 41 |
| 73 | 100 | 81 | 42 | 69 | ND |
| 74 | 28 | 35 | 132 | ND | 36 |

Annex 2.1.A. Biochemistry results (ALT values [U/L]) of chronic hepatitis B patients treated with PEGIFN. *Reference range*: M[up to 29] / F[up to 19], 2^nd^ part.

| I.N. | W0 | W12 | W24 | W48 | W76 |
| --- | --- | --- | --- | --- | --- |
| 81 | 37 | 62 | 32 | 34 | 30 |
| 83 | 29 | 119 | 67 | 47 | 22 |
| 86 | 40 | 27 | 50 | 28 | 39 |
| 87 | 38 | 136 | 32 | 56 | 80 |
| 93 | 77 | 33 | 20 | 20 | 46 |
| 94 | 48 | 229 | 159 | 62 | 54 |
| 97 | 79 | 134 | 63 | 36 | 172 |
| 98 | 38 | 38 | 41 | 22 | 25 |
| 99 | 29 | 34 | 24 | 30 | 30 |
| 100 | 62 | 77 | 41 | 39 | ND |
| 102 | 28 | 33 | 34 | 16 | 23 |
| 104 | 40 | 71 | 28 | DROPPED | ND |
| 105 | 44 | 73 | 64 | 36 | 39 |
| 107 | 86 | 26 | 35 | 33 | ND |
| 109 | 37 | DROPPED | DROPPED | DROPPED | DROPPED |
| 111 | 40 | 37 | 35 | 80 | ND |
| 114 | 55 | 40 | 30 | ND | 24 |
| 115 | 71 | 76 | 70 | 59 | ND |
| 117 | 50 | 48 | 33 | 24 | 55 |
| 118 | 29 | 89 | 43 | 48 | 41 |
| 121 | 36 | 142 | 56 | 61 | 104 |
| 122 | 23 | 46 | ND | 19 | ND |
| 124 | 32 | 40 | ND | 32 | 32 |
| 128 | 226 | 53 | 38 | 33 | ND |
| 130 | 34 | 47 | 48 | 37 | 39 |
| 132 | 18 | 23 | 23 | 23 | 18 |
| 134 | 22 | 38 | 86 | 31 | 29 |
| 136 | 24 | 31 | 18 | ND | ND |
| 138 | 39 | 91 | 83 | 43 | 30 |
| 139 | 33 | 57 | 55 | 21 | 26 |
| 141 | 38 | 60 | 60 | 40 | 26 |
| 143 | 88 | 48 | 51 | ND | 235 |
| 144 | 44 | 71 | 41 | 20 | 37 |
| 145 | 22 | 21 | 21 | 21 | 18 |
| 146 | 15 | 30 | 29 | 11 | 34 |
| 147 | 25 | 38 | 34 | ND | 28 |
| 148 | 56 | 39 | 28 | 33 | 38 |
| 154 | 46 | 55 | 42 | ND | ND |
| 155 | 22 | 49 | 49 | 28 | 29 |
| 156 | 31 | 37 | 35 | 42 | ND |

Annex 2.1.B. Biochemistry results (ALT values [U/L]) of chronic hepatitis B patients treated with NASVAC. *Reference range*: M[up to 29] / F[up to 19].

| I.N. | W0 | W12 | W24 | W48 | W76 |
| --- | --- | --- | --- | --- | --- |
| 1 | 17 | 124 | 25 | 25 | ND |
| 3 | 128 | 173 | 33 | 31 | 24 |
| 5 | 15 | 108 | 35 | 32 | 32 |
| 7 | 21 | 83 | 25 | 20 | 42 |
| 8 | 24 | 44 | 24 | ND | ND |
| 10 | 14 | 166 | 22 | 29 | 29 |
| 11 | 23 | 140 | 50 | 39 | 27 |
| 14 | 30 | 102 | 32 | 55 | 23 |
| 18 | 201 | 148 | 20 | 17 | 25 |
| 20 | 25 | 156 | 33 | 18 | 46 |
| 23 | 30 | 115 | 38 | 34 | 46 |
| 24 | 20 | 120 | 22 | 18 | 17 |
| 25 | 28 | 119 | 16 | 60 | 22 |
| 27 | 20 | 87 | 36 | 37 | 28 |
| 29 | 36 | 172 | 35 | 37 | 31 |
| 30 | 23 | 119 | 31 | 25 | 33 |
| 32 | 15 | 94 | 30 | 29 | 42 |
| 38 | 36 | 187 | 19 | 28 | 33 |
| 39 | 21 | DROPPED | DROPPED | DROPPED | DROPPED |
| 40 | 22 | 77 | 61 | 64 | 72 |
| 45 | 30 | 122 | 28 | 20 | ND |
| 48 | 36 | 157 | 36 | 45 | 20 |
| 49 | 101 | 67 | 62 | 71 | 107 |
| 50 | 35 | 190 | 43 | 18 | 53 |
| 54 | 15 | 35 | 19 | 47 | ND |
| 56 | 19 | 144 | 38 | 34 | 53 |
| 57 | 30 | 164 | 41 | 81 | 63 |
| 58 | 43 | 35 | 44 | 66 | ND |
| 59 | 41 | 169 | 67 | 38 | 28 |
| 60 | NP | NP | NP | NP | NP |
| 67 | 22 | 167 | 31 | 30 | ND |
| 68 | 45 | 114 | 37 | 39 | 30 |
| 69 | NP | NP | NP | NP | NP |
| 72 | 32 | 170 | 38 | 83 | 26 |
| 75 | 30 | 153 | 36 | 42 | 48 |
| 76 | 16 | 79 | 38 | 30 | 18 |
| 77 | 35 | 140 | 55 | 33 | 48 |
| 78 | 10 | 131 | 64 | 46 | 55 |
| 79 | 23 | 187 | 23 | 26 | 71 |
| 80 | 19 | 32 | ND | 28 | 27 |

Annex 2.1.B. Biochemistry results (ALT values [U/L]) of chronic hepatitis B patients treated with NASVAC. *Reference range*: M[up to 29] / F[up to 19], 2^nd^ part.

| I.N. | W0 | W12 | W24 | W48 | W76 |
| --- | --- | --- | --- | --- | --- |
| 82 | 13 | 122 | 21 | 26 | 18 |
| 84 | 18 | 128 | 32 | 20 | 17 |
| 85 | 14 | 154 | 27 | 18 | ND |
| 88 | 18 | 128 | 22 | 22 | 27 |
| 89 | 20 | 113 | 35 | 39 | 55 |
| 90 | 26 | 155 | 48 | 74 | 58 |
| 91 | 27 | 12 | 28 | 30 | 23 |
| 92 | 12 | 63 | 29 | 37 | 19 |
| 95 | 34 | 87 | 36 | 36 | 24 |
| 96 | 62 | 155 | 40 | 32 | 31 |
| 101 | 49 | 97 | 38 | 28 | ND |
| 103 | 58 | 179 | 229 | 58 | 165 |
| 106 | 43 | 151 | 46 | 23 | 52 |
| 108 | 262 | 222 | 132 | 67 | 51 |
| 110 | 193 | 187 | 43 | 163 | 92 |
| 112 | 26 | 125 | ND | ND | ND |
| 113 | 23 | 46 | 31 | 22 | 26 |
| 116 | 24 | 171 | 34 | 37 | 33 |
| 119 | 94 | 180 | 95 | 43 | 152 |
| 120 | 78 | 116 | 34 | 16 | ND |
| 123 | 33 | 162 | 36 | 35 | 31 |
| 125 | 50 | 38 | 45 | 50 | 43 |
| 126 | 17 | 140 | 29 | 24 | 39 |
| 127 | 47 | 140 | 34 | 34 | 30 |
| 129 | 35 | 100 | 42 | 50 | ND |
| 131 | 40 | DROPPED | DROPPED | DROPPED | DROPPED |
| 133 | 74 | 139 | 53 | 31 | 66 |
| 135 | 41 | 59 | 56 | 33 | 28 |
| 137 | 22 | 94 | 26 | 47 | 30 |
| 140 | 77 | 165 | 37 | 79 | 28 |
| 142 | 24 | 114 | 25 | 29 | 36 |
| 149 | 16 | 141 | 28 | ND | ND |
| 150 | 80 | 40 | 37.5 | 29 | 32 |
| 151 | 31 | 40 | 36 | 33 | 25 |
| 152 | 15 | DROPPED | DROPPED | DROPPED | DROPPED |
| 153 | 48 | 30 | 28 | 36 | 50 |
| 157 | 39 | 43 | 43 | 33 | 46 |
| 158 | 35 | 40 | 44 | 43 | ND |
| 159 | 35 | 159 | 20 | 22 | 28 |
| 160 | 65 | 23 | 31 | 40 | ND |

Annex 2.2.A. Biochemistry results (AST values [U/L]) of chronic hepatitis B patients treated with PEGIFN. *Reference range*: M[up to 39] / F[up to 41].

| I.N. | Baseline | W24 | W48 |
| --- | --- | --- | --- |
| 2 | 23 | 47 | 21 |
| 4 | 29 | 22 | 15 |
| 6 | 25 | 30 | 28 |
| 9 | 13 | 50 | 46 |
| 12 | 18 | 60 | 49 |
| 13 | 32 | 40 | 63 |
| 15 | 19 | 81 | 51 |
| 16 | 24 | DROPPED | DROPPED |
| 17 | 18 | 51 | 40 |
| 19 | 40 | ND | 36 |
| 21 | 45 | 40 | 35 |
| 22 | 48 | 26 | 44 |
| 26 | 25 | 30 | 22 |
| 28 | 36 | 60 | 42 |
| 31 | 60 | 41 | 37 |
| 33 | 51 | 18 | 24 |
| 34 | 44 | 62 | 40 |
| 35 | 29 | 80 | 47 |
| 36 | 48 | 34 | 28 |
| 37 | 98 | 118 | 149 |
| 41 | 27 | 30 | 49 |
| 42 | 42 | 57 | 41 |
| 43 | 33 | 92 | 60 |
| 44 | 156 | 63 | 43 |
| 46 | 27 | 46 | 30 |
| 47 | 23 | 19 | 21 |
| 51 | 30 | 49 | 127 |
| 52 | 44 | 135 | 55 |
| 53 | 18 | DROPPED | DROPPED |
| 55 | 27 | 80 | ND |
| 61 | 29 | 51 | 38 |
| 62 | 22 | 69 | 73 |
| 63 | 40 | 34 | 44 |
| 64 | 46 | 43 | 36 |
| 65 | 43 | 36 | ND |
| 66 | 41 | 25 | 30 |
| 70 | 26 | 23 | 29 |
| 71 | 35 | 37 | 33 |
| 73 | 43 | 49 | 60 |
| 74 | 25 | 129 | ND |

Annex 2.2.A. Biochemistry results (AST values [U/L]) of chronic hepatitis B patients treated with PEGIFN. *Reference range*: M[up to 39] / F[up to 41], 2^nd^ part.

| I.N. | Baseline | W24 | W48 |
| --- | --- | --- | --- |
| 81 | 35 | 29 | 41 |
| 83 | 28 | 65 | 44 |
| 86 | 38 | 48 | 38 |
| 87 | 30 | 29 | 50 |
| 93 | 56 | 16 | 22 |
| 94 | 46 | 154 | 55 |
| 97 | 59 | 57 | 40 |
| 98 | 40 | 46 | 28 |
| 99 | 29 | 22 | 39 |
| 100 | 56 | 38 | ND |
| 102 | 25 | 31 | 21 |
| 104 | 38 | 25 | DROPPED |
| 105 | 40 | 62 | 33 |
| 107 | 59 | 28 | 28 |
| 109 | 34 | DROPPED | DROPPED |
| 111 | 37 | 33 | 69 |
| 114 | 60 | ND | ND |
| 115 | 43 | 45 | 44 |
| 117 | 48 | 29 | 36 |
| 118 | 27 | 39 | 40 |
| 121 | 30 | 62 | 57 |
| 122 | 19 | ND | 20 |
| 124 | 30 | ND | 30 |
| 128 | 215 | 36 | 43 |
| 130 | 33 | 44 | 72 |
| 132 | 25 | 20 | 30 |
| 134 | 20 | 84 | 55 |
| 136 | 22 | 15 | ND |
| 138 | 37 | 80 | 38 |
| 139 | 32 | 53 | 25 |
| 141 | 36 | 51 | 42 |
| 143 | 49 | 51 | ND |
| 144 | 43 | 38 | 36 |
| 145 | 20 | 18 | 18 |
| 146 | 14 | 27 | 15 |
| 147 | 24 | 33 | ND |
| 148 | 48 | 25 | 36 |
| 154 | 37 | 53 | ND |
| 155 | 27 | 58 | 26 |
| 156 | 26 | 29 | 30 |

Annex 2.2.B. Biochemistry results (AST values [U/L]) of chronic hepatitis B patients treated with NASVAC. *Reference range*: M[up to 39] / F[up to 41].

| I.N. | Baseline | W24 | W48 |
| --- | --- | --- | --- |
| 1 | 16 | 22 | 35 |
| 3 | 122 | 30 | 28 |
| 5 | 15 | 32 | 28 |
| 7 | 19 | 22 | 18 |
| 8 | 22 | 22 | ND |
| 10 | 10 | 20 | 26 |
| 11 | 19 | 50 | 35 |
| 14 | 28 | 29 | 39 |
| 18 | 189 | 17 | 29 |
| 20 | 22 | 30 | 37 |
| 23 | 33 | 35 | 33 |
| 24 | 15 | 19 | 40 |
| 25 | 21 | 14 | 57 |
| 27 | 19 | 33 | 34 |
| 29 | 35 | 33 | 33 |
| 30 | 20 | 28 | 23 |
| 32 | 12 | 26 | 20 |
| 38 | 34 | 39 | 26 |
| 39 | DROPPED | DROPPED | DROPPED |
| 40 | 19 | 33 | 46 |
| 45 | 28 | 25 | 17 |
| 48 | 33 | 32 | 41 |
| 49 | 54 | 45 | 66 |
| 50 | 30 | 40 | 20 |
| 54 | 23 | 25 | 45 |
| 56 | 14 | 36 | 31 |
| 57 | 29 | 39 | 45 |
| 58 | 40 | 41 | 36 |
| 59 | 40 | 35 | 35 |
| 60 | NP | NP | NP |
| 67 | 20 | 29 | 18 |
| 68 | 43 | 32 | 36 |
| 69 | NP | NP | NP |
| 72 | 29 | 35 | 79 |
| 75 | 27 | 35 | 41 |
| 76 | 15 | 35 | 32 |
| 77 | 34 | 53 | 30 |
| 78 | 8 | 63 | 26 |
| 79 | 22 | 21 | 23 |
| 80 | 30 | ND | 23 |

Annex 2.2.B. Biochemistry results (AST values [U/L]) of chronic hepatitis B patients treated with NASVAC. *Reference range*: M[up to 39] / F[up to 41], 2^nd^ part.

| I.N. | Baseline | W24 | W48 |
| --- | --- | --- | --- |
| 82 | 9 | 19 | 24 |
| 84 | 15 | 30 | 31 |
| 85 | 10 | 23 | 21 |
| 88 | 16 | 19 | 18 |
| 89 | 17 | 32 | 36 |
| 90 | 22 | 44 | 66 |
| 91 | 22 | 25 | 27 |
| 92 | 10 | 27 | 33 |
| 95 | 32 | 33 | 34 |
| 96 | 59 | 36 | 28 |
| 101 | 44 | 35 | 18 |
| 103 | 51 | 223 | 52 |
| 106 | 40 | 43 | 22 |
| 108 | 247 | 127 | 62 |
| 110 | 184 | 39 | 90 |
| 112 | 27 | ND | ND |
| 113 | 18 | 25 | 18 |
| 116 | 20 | 33 | 34 |
| 119 | 90 | 93 | 39 |
| 120 | 72 | 31 | 13 |
| 123 | 30 | 41 | 31 |
| 125 | 43 | 27 | 47 |
| 126 | 15 | 27 | 20 |
| 127 | 42 | 33 | 31 |
| 129 | 33 | 39 | ND |
| 131 | 39 | DROPPED | DROPPED |
| 133 | 72 | 59 | 28 |
| 135 | 41 | 53 | 30 |
| 137 | 18 | 20 | 42 |
| 140 | 69 | 35 | 77 |
| 142 | 27 | 22 | 37 |
| 149 | 12 | 22 | ND |
| 150 | 78 | 29 | 33 |
| 151 | 11 | 29 | 29 |
| 152 | 22 | DROPPED | DROPPED |
| 153 | 45 | 37 | 32 |
| 157 | 29 | ND | 30 |
| 158 | 23 | 29 | 41 |
| 159 | 30 | 33 | 19 |
| 160 | 60 | 25 | 34 |

Annex 2.3.A. Liver function tests (Bilirubin levels [mg/dL]) of chronic hepatitis B patients treated with PEGIFN. *Reference range*:[0.3-1.1].

| I.N. | Baseline | W12 | W24 | W48 |
| --- | --- | --- | --- | --- |
| 2 | 0,46 | 0,64 | 0,40 | 0,40 |
| 4 | 0,72 | 0.59 | 0,48 | 0,70 |
| 6 | 0,40 | 0,58 | 0,68 | 0,40 |
| 9 | 0,39 | 0,56 | 0,40 | 0,40 |
| 12 | 0,27 | 0,50 | 0,76 | 0,78 |
| 13 | 0,49 | 0,66 | 0,58 | 0,64 |
| 15 | 0,46 | 0,83 | 1,10 | ND |
| 16 | DROPPED | DROPPED | DROPPED | DROPPED |
| 17 | 0,50 | 0,78 | 0,55 | 0,50 |
| 19 | 0,49 | 0,6 | 0,5 | 0,50 |
| 21 | 0,89 | 0,54 | 0,66 | 0,77 |
| 22 | 0,78 | 0,67 | 0,78 | 0,82 |
| 26 | 0,28 | 0,72 | 0,65 | 0,66 |
| 28 | 0,48 | 0,73 | 0,83 | 0,50 |
| 31 | 0,89 | 0,75 | 0,66 | 0,80 |
| 33 | 0,52 | 0,73 | 0,62 | 0,72 |
| 34 | 0,75 | 0,83 | 0,70 | 0.52 |
| 35 | 0,58 | 0,45 | 0,86 | 0,40 |
| 36 | 0,50 | 0,65 | 0,85 | 0,73 |
| 37 | ND | 0,49 | 0,64 | 0,45 |
| 41 | 0,44 | 0,62 | 0,75 | 0,55 |
| 42 | 0,80 | 0,68 | 0,70 | 0,62 |
| 43 | 0,59 | 0,63 | 0,77 | 0,65 |
| 44 | 0,72 | 0,77 | 0,74 | 0,40 |
| 46 | 0,44 | 0,48 | 0,73 | 0,55 |
| 47 | 0,50 | 0,73 | 0,62 | 0,80 |
| 51 | 0,39 | 0,68 | 0,40 | 0,40 |
| 52 | 0,35 | 0,71 | 1,10 | 0,45 |
| 53 | 0,65 | 0,69 | DROPPED | DROPPED |
| 55 | 0,45 | 0,52 | 0,50 | ND |
| 61 | 0,39 | 0,70 | 0,50 | 1,20 |
| 62 | 0,62 | 0,68 | 0,50 | 0,45 |
| 63 | 0,45 | 0,68 | 0,62 | 0,65 |
| 64 | 0,32 | 0,77 | 0,60 | 0.52 |
| 65 | 0,58 | 0,66 | 0,59 | ND |
| 66 | 0,46 | 0,83 | 0,62 | 0,72 |
| 70 | 0,38 | 0,57 | 0,58 | 0,65 |
| 71 | 0,37 | 0,69 | 0,52 | 0,80 |
| 73 | 0,30 | 1,00 | 1,00 | 0,90 |
| 74 | 0,50 | 0,72 | 0,84 | ND |

Annex 2.3.A. Liver function tests (Bilirubin levels [mg/dL]) of chronic hepatitis B patients treated with PEGIFN. *Reference range*:[0.3-1.1], 2^nd^ part.

| I.N. | Baseline | W12 | W24 | W48 |
| --- | --- | --- | --- | --- |
| 81 | 0,59 | 0,71 | 0,61 | 0,68 |
| 83 | 0,45 | 1,13 | 0,72 | 0,54 |
| 86 | 0,24 | 0,58 | 0,73 | 0,66 |
| 87 | ND | 0,71 | 0,57 | 0,67 |
| 93 | 0,49 | 0,82 | 0,67 | 0,67 |
| 94 | 0,50 | 0,67 | 0,68 | 0,77 |
| 97 | 0,40 | 0,53 | 0,73 | 0,80 |
| 98 | 0,48 | 0,65 | 0,45 | 0,69 |
| 99 | 0,38 | 0,52 | 0,78 | 0,40 |
| 100 | 0,42 | 0,92 | 0,93 | 1,10 |
| 102 | 0,48 | 0,66 | 0,72 | 0,68 |
| 104 | 0,39 | 0,69 | 0,52 | ND |
| 105 | 0,81 | 0,65 | 0,68 | 0,66 |
| 107 | 0,32 | 0,64 | 0,62 | 0,60 |
| 109 | 0,35 | DROPPED | DROPPED | DROPPED |
| 111 | 0,28 | 0,45 | 0,72 | 0,40 |
| 114 | 0,78 | 0,70 | 0,90 | 0,80 |
| 115 | 0,28 | 0.24 | 1,50 | 0,54 |
| 117 | 0,52 | 1,04 | 0,83 | 0,74 |
| 118 | 0,45 | 0,76 | 0,81 | 0,55 |
| 121 | 0,93 | 0,73 | 0,40 | 0,55 |
| 122 | 0,90 | 0,65 | ND | 0,64 |
| 124 | 0,55 | 0,46 | ND | 0,55 |
| 128 | 0,38 | 0,62 | 0,78 | 0,50 |
| 130 | 0,45 | 0,70 | 0,62 | 0,72 |
| 132 | 0,81 | 0,62 | 0,62 | 0,45 |
| 134 | 0,59 | 0,61 | 0,60 | 0,58 |
| 136 | 0,52 | 0,68 | 0,70 | ND |
| 138 | 0,28 | 0,70 | 0,62 | 0,90 |
| 139 | 0,49 | 0,67 | 0,78 | 0,80 |
| 141 | 0,45 | 0,50 | 0,70 | 1,55 |
| 143 | 0,52 | 0,55 | 0,50 | ND |
| 144 | 0,39 | 0,56 | 0,55 | 0,82 |
| 145 | 0,62 | 0,68 | 0,52 | 0,68 |
| 146 | 0,75 | 0,85 | 0,55 | 0,74 |
| 147 | 0,65 | 0,83 | 0,80 | ND |
| 148 | 0,62 | 0,62 | 0,72 | 0,30 |
| 154 | 0,40 | 0,66 | 0,80 | ND |
| 155 | 0,86 | 0,52 | 0,59 | 0,40 |
| 156 | 0,52 | 0,83 | 0,74 | 0,70 |

Annex 2.4.B. Liver function tests (Bilirubin levels [mg/dL]) of chronic hepatitis B patients treated with NASVAC. *Reference range*:[0.3-1.1].

| I.N. | Baseline | W12 | W24 | W48 |
| --- | --- | --- | --- | --- |
| 1 | 0.59 | 0.75 | 0.48 | 0.4 |
| 3 | 0.59 | 0.7 | 0.65 | 0.66 |
| 5 | 0.68 | 0.62 | 0.56 | 0.78 |
| 7 | 0.66 | 0.7 | 0.72 | 0.66 |
| 8 | 0.96 | 1.1 | 0.72 | ND |
| 10 | 0.51 | 0.68 | 1.16 | 0.68 |
| 11 | 0.72 | 0.66 | 0.7 | 0.66 |
| 14 | 0.91 | 0.82 | 0.42 | 0.4 |
| 18 | 0.47 | 0.72 | 0.6 | 0.4 |
| 20 | 0.61 | 0.7 | 0.62 | 0.6 |
| 23 | 0.73 | 0.64 | 0.73 | 0.74 |
| 24 | 0.7 | 0.68 | 0.58 | 0.5 |
| 25 | 0.63 | 0.74 | 0.62 | 0.88 |
| 27 | 0.67 | 0.72 | 0.49 | 0.67 |
| 29 | 0.57 | 0.67 | 0.63 | 0.84 |
| 30 | 0.78 | 0.89 | 0.45 | 0.4 |
| 32 | 0.42 | 0.72 | 0.5 | 0.5 |
| 38 | 0.81 | 0.72 | 0.75 | 0.6 |
| 39 | DROPPED | DROPPED | DROPPED | DROPPED |
| 40 | 0.52 | 0.67 | 0.72 | 0.65 |
| 45 | 0.73 | 0.62 | 0.76 | 0.56 |
| 48 | 0.47 | 0.62 | 0.82 | 0.58 |
| 49 | 0.7 | 0.6 | 0.57 | 0.86 |
| 50 | 0.47 | 0.97 | 0.71 | 0.78 |
| 54 | 0.72 | 0.6 | 0.92 | 0.78 |
| 56 | 0.79 | 0.58 | 0.58 | 0.75 |
| 57 | 0.51 | 0.78 | 0.57 | 0.4 |
| 58 | 0.62 | 0.58 | 0.72 | 0.55 |
| 59 | 0.69 | 0.72 | 0.6 | 0.68 |
| 60 | N. I. | N. I. | N. I. | N. I. |
| 67 | 0.71 | 0.62 | 0.75 | 0.95 |
| 68 | 0.67 | 0.74 | 1.06 | 0.66 |
| 69 | N. I. | N. I. | N. I. | N. I. |
| 72 | 0.74 | 0.72 | 0.66 | 0.72 |
| 75 | 0.82 | 0.72 | 0.68 | 1.1 |
| 76 | 0.59 | 0.61 | 0.62 | 0.35 |
| 77 | 0.53 | 0.9 | 0.73 | 0.64 |
| 78 | 0.62 | 0.68 | 0.48 | 0.35 |
| 79 | 0.58 | 0.55 | 0.65 | 0.62 |
| 80 | 0.67 | 0.7 | ND | 0.63 |

Annex 2.4.B. Liver function tests (Bilirubin levels [mg/dL]) of chronic hepatitis B patients treated with NASVAC. *Reference range*:[0.3-1.1], 2^nd^ part.

| I.N. | Baseline | W12 | W24 | W48 |
| --- | --- | --- | --- | --- |
| 82 | 1.2 | 0.75 | 0.72 | 0.78 |
| 84 | 0.72 | 0.73 | 0.7 | 0.4 |
| 85 | 0.42 | 0.54 | 0.83 | 0.9 |
| 88 | 0.63 | 0.65 | 0.72 | 0.4 |
| 89 | 0.62 | 0.58 | 0.58 | 0.72 |
| 90 | 0.56 | 0.68 | 0.52 | 0.58 |
| 91 | 0.82 | 0.67 | 0.8 | 0.84 |
| 92 | 0.57 | 0.63 | 0.66 | 0.84 |
| 95 | 0.59 | 0.72 | 0.66 | 1 |
| 96 | 0.91 | 0.88 | 0.77 | 0.74 |
| 101 | 0.72 | 0.72 | 0.73 | 0.35 |
| 103 | 0.61 | 0.55 | 0.68 | 0.59 |
| 106 | 0.85 | 0.67 | 0.52 | 0.7 |
| 108 | 0.39 | 0.82 | 0.88 | 0.77 |
| 110 | 0.67 | 0.73 | 0.56 | 0.4 |
| 112 | 0.73 | 0.75 | ND | ND |
| 113 | 0.62 | 0.5 | 0.92 | 0.64 |
| 116 | 0.72 | 0.72 | 0.74 | 0.64 |
| 119 | 0.56 | 0.65 | 0.56 | 0.95 |
| 120 | 0.73 | 0.69 | 0.73 | 0.86 |
| 123 | 0.53 | 0.63 | 0.4 | 0.66 |
| 125 | 0.76 | 0.82 | 0.72 | 0.64 |
| 126 | 0.78 | 0.82 | 0.66 | 0.82 |
| 127 | 0.61 | 0.71 | 0.74 | 0.66 |
| 129 | 0.58 | 0.62 | 0.68 | 0.4 |
| 131 | 0.73 | DROPPED | DROPPED | DROPPED |
| 133 | 0.63 | 0.61 | 0.85 | 0.68 |
| 135 | 0.73 | 0.73 | 0.53 | 0.64 |
| 137 | 0.72 | 0.62 | 0.68 | 0.4 |
| 140 | 0.59 | 0.54 | 0.72 | 0.64 |
| 142 | 0.71 | 0.67 | 0.5 | 0.55 |
| 149 | 0.62 | 0.58 | 0.77 | ND |
| 150 | 0.59 | 0.55 | 0.53 | 0.45 |
| 151 | 0.4 | 0.6 | 0.4 | 0.4 |
| 152 | 0.72 | DROPPED | DROPPED | DROPPED |
| 153 | 1.19 | 1.47 | 0.64 | 0.6 |
| 157 | 0.8 | 0.9 | 0.9 | 0.8 |
| 158 | 1.8 | 1.88 | 1.52 | 0.62 |
| 159 | 1.26 | 1.67 | 1.84 | 0.82 |
| 160 | 0.67 | 0.79 | 0.56 | 0.56 |

Annex 2.5.A. Liver function tests (Alc. phosphatase [U/L]) levels of chronic hepatitis B patients treated with PEGIFN. *Reference range*:[80-290].

| I.N. | Baseline | W24 | W48 |
| --- | --- | --- | --- |
| 2 | 70 | 32 | 49 |
| 4 | 115 | 120 | 325 |
| 6 | 121 | 74 | 70 |
| 9 | 65 | 47 | 49 |
| 12 | 106 | 106 | 275 |
| 13 | 105 | 128 | 185 |
| 15 | 95 | 92 | ND |
| 16 | 162 | DROPPED | DROPPED |
| 17 | 128 | 129 | 91 |
| 19 | 87 | ND | 44 |
| 21 | 79 | 66 | 203 |
| 22 | 86 | 106 | 98 |
| 26 | 108 | 89 | 127 |
| 28 | 116 | 58 | 50 |
| 31 | 126 | 177 | 170 |
| 33 | 32 | 182 | 132 |
| 34 | 141 | 120 | 102 |
| 35 | 71 | 287 | 68 |
| 36 | 87 | 68 | 113 |
| 37 | 93 | 236 | 157 |
| 41 | 171 | 379 | 90 |
| 42 | 79 | 62 | 170 |
| 43 | 119 | 153 | 75 |
| 44 | 97 | 126 | 78 |
| 46 | 100 | 87 | 126 |
| 47 | 107 | 118 | 129 |
| 51 | 85 | 87 | 56 |
| 52 | 68 | 88 | 73 |
| 53 | 132 | DROPPED | DROPPED |
| 55 | 69 | 57 | ND |
| 61 | 107 | 61 | 71 |
| 62 | 111 | 110 | 106 |
| 63 | 125 | 122 | 239 |
| 64 | 121 | 50 | 45 |
| 65 | 175 | 135 | ND |
| 66 | 120 | 120 | 205 |
| 70 | 150 | 96 | 229 |
| 71 | 96 | 65 | 129 |
| 73 | 86 | ND | 29 |
| 74 | 109 | 53 | ND |

Annex 2.5.A. Liver function tests (Alc. phosphatase [U/L] levels of chronic hepatitis B patients treated with PEGIFN. *Reference range*:[80-290], 2^nd^ part.

| I.N. | Baseline | W24 | W48 |
| --- | --- | --- | --- |
| 81 | 110 | 103 | 222 |
| 83 | 147 | 95 | 63 |
| 86 | 100 | 96 | 134 |
| 87 | 98 | 120 | 190 |
| 93 | 135 | 87 | 140 |
| 94 | 87 | 59 | 106 |
| 97 | 102 | 84 | 169 |
| 98 | 120 | 85 | 124 |
| 99 | 128 | 131 | 74 |
| 100 | 108 | 86 | ND |
| 102 | 104 | 68 | 125 |
| 104 | 119 | 79 | ND |
| 105 | 75 | 77 | 119 |
| 107 | 127 | 141 | 175 |
| 109 | 93 | DROPPED | DROPPED |
| 111 | 150 | 79 | 79 |
| 114 | 171 | ND | ND |
| 115 | 103 | 48 | 72 |
| 117 | 131 | 146 | 124 |
| 118 | 86 | 115 | 75 |
| 121 | 190 | 121 | 117 |
| 122 | 210 | ND | 185 |
| 124 | 105 | ND | 105 |
| 128 | 118 | 255 | 72 |
| 130 | 101 | 100 | 225 |
| 132 | 125 | 80 | 47 |
| 134 | 76 | 62 | 153 |
| 136 | 87 | 102 | ND |
| 138 | 106 | 90 | 68 |
| 139 | 104 | 110 | 112 |
| 141 | 81 | 59 | 43 |
| 143 | 89 | 70 | ND |
| 144 | 136 | 160 | 127 |
| 145 | 133 | 69 | 168 |
| 146 | 78 | 85 | 134 |
| 147 | 66 | 97 | ND |
| 148 | 167 | 95 | 59 |
| 154 | 187 | 93 | ND |
| 155 | 149 | 141 | 88 |
| 156 | 210 | 233 | 180 |

Annex 2.5.B. Liver function tests (Alc. phosphatase [U/L]) levels of chronic hepatitis B patients treated with NASVAC. *Reference range*:[80-290].

| I.N. | Baseline | W24 | W48 |
| --- | --- | --- | --- |
| 1 | 74 | 96 | 58 |
| 3 | 277 | 142 | 189 |
| 5 | 76 | 144 | 244 |
| 7 | 161 | 92 | 102 |
| 8 | 78 | 127 | ND |
| 10 | 95 | 97 | 152 |
| 11 | 96 | 120 | 274 |
| 14 | 147 | 170 | 59 |
| 18 | 161 | 110 | 101 |
| 20 | 134 | 152 | 90 |
| 23 | 199 | 107 | 180 |
| 24 | 103 | 133 | 87 |
| 25 | 107 | 108 | 255 |
| 27 | 131 | 121 | 179 |
| 29 | 245 | 132 | 121 |
| 30 | 137 | 103 | 94 |
| 32 | 68 | 56 | 50 |
| 38 | 93 | 57 | 156 |
| 39 | DROPPED | DROPPED | DROPPED |
| 40 | 165 | 53 | 78 |
| 45 | 156 | 126 | 112 |
| 48 | 160 | 80 | 189 |
| 49 | 363 | 89 | 255 |
| 50 | 83 | 106 | 179 |
| 54 | 337 | 111 | 76 |
| 56 | 136 | 128 | 114 |
| 57 | 95 | 166 | 64 |
| 58 | 250 | 136 | 94 |
| 59 | 99 | 91 | 187 |
| 60 | NP | NP | NP |
| 67 | 109 | 60 | 62 |
| 68 | 97 | 74 | 178 |
| 69 | NP | NP | NP |
| 72 | 179 | 122 | 197 |
| 75 | 291 | 63 | 125 |
| 76 | 78 | 121 | 64 |
| 77 | 148 | 108 | 119 |
| 78 | 103 | 220 | 89 |
| 79 | 86 | 122 | 70 |
| 80 | 144 | ND | 234 |

Annex 2.5.B. Liver function tests (Alc. phosphatase [U/L]) levels of chronic hepatitis B patients treated with NASVAC. *Reference range*:[80-290], 2^nd^ part.

| I.N. | Baseline | W24 | W48 |
| --- | --- | --- | --- |
| 82 | 73 | 162 | 119 |
| 84 | 93 | 94 | 93 |
| 85 | 190 | 105 | 55 |
| 88 | 221 | 109 | 85 |
| 89 | 190 | 128 | 140 |
| 90 | 124 | 87 | 230 |
| 91 | 181 | 106 | 94 |
| 92 | 186 | 160 | 121 |
| 95 | 162 | 132 | 42 |
| 96 | 96 | 108 | 214 |
| 101 | 190 | 142 | 89 |
| 103 | 273 | 258 | 242 |
| 106 | 196 | 112 | 240 |
| 108 | 293 | 154 | 187 |
| 110 | 220 | 126 | 123 |
| 112 | 172 | ND | ND |
| 113 | 332 | 87 | 212 |
| 116 | 113 | 196 | 94 |
| 119 | 73 | 182 | 71 |
| 120 | 69 | 107 | 128 |
| 123 | 222 | 81 | 198 |
| 125 | 173 | 85 | 237 |
| 126 | 172 | 87 | 97 |
| 127 | 136 | 72 | 167 |
| 129 | 249 | 146 | 178 |
| 131 | 181 | DROPPED | DROPPED |
| 133 | 390 | 73 | 210 |
| 135 | 89 | 153 | 119 |
| 137 | 64 | 125 | 45 |
| 140 | 240 | 145 | 203 |
| 142 | 317 | 161 | 56 |
| 149 | 220 | 87 | ND |
| 150 | 210 | 264 | 285 |
| 151 | 87 | 79 | 98 |
| 152 | 210 | DROPPED | DROPPED |
| 153 | 183 | 210 | 71 |
| 157 | 90 | ND | 98 |
| 158 | 270 | 71 | 270 |
| 159 | 202 | 196 | 61 |
| 160 | 236 | 209 | 263 |
