# Supplementary material for: Treatment of chronic hepatitis B naïve patients with a therapeutic vaccine containing HBs and HBc antigens (a randomized, open and treatment controlled phase III clinical trial)
Source: PLoS One. 2018 Aug 22;13(8):e0201236. doi: 10.1371/journal.pone.0201236 (PMC6104936; doi:10.1371/journal.pone.0201236)
Supplement: S3 Table — The file contains the results of adverse events detected during the study. (DOC) [file pone.0201236.s006.doc]

**S3 Table. ANNEX 3 Adverse Events.doc to your manuscript.  The file contains the results of adverse events detected during the study.**

**Supporting information can be accessed at: Aguilar, Julio, 2018, "Replication Data for: Anonymized data set",** [**https://doi.org/10.7910/DVN/IT467W**](https://doi.org/10.7910/DVN/IT467W)**, Harvard Dataverse, V1.**

**Annex 3.1.A.** Adverse events (AE) during PegIFN treatment.

**Annex 3.2.A.** Adverse events reported during PegIFN administration, weekly report of the number of cases reported during the 48 weeks of product administration.

**Annex 3.1.B.** Adverse events during NASVAC treatment.

**Annex 3.2.B.** Adverse events reported during NASVAC administration, bi-monthly report of the number of cases reported during the 10 product administrations.

| **No.** | **AE-ID** | **Adverse Event** | **Total (N)** | **Pat/AE** | **%** |
| --- | --- | --- | --- | --- | --- |
| 1 | 8 | Fever | 247 | 78 | 97,5 |
| 2 | 13 | Weakness | 317 | 66 | 82,5 |
| 3 | 9 | General Malaise | 195 | 65 | 81,25 |
| 4 | 10 | Headache | 60 | 35 | 43,75 |
| 5 | 1 | Local Pain | 42 | 23 | 28,75 |
| 6 | 11 | Nausea | 25 | 18 | 22,5 |
| 7 | 25 | Lose Motion | 13 | 12 | 15 |
| 8 | 29 | Hairfall | 16 | 9 | 11,25 |
| 9 | 28 | Fatigue | 10 | 8 | 10 |
| 10 | 30 | Ansiety | 10 | 8 | 10 |
| 11 | 37 | GIT disorder | 12 | 8 | 10 |
| 12 | 35 | Dyspepsia | 10 | 7 | 8,75 |
| 13 | 31 | Apthous ulcer | 6 | 6 | 7,5 |
| 14 | 41 | Skin Rash | 6 | 6 | 7,5 |
| 15 | 58 | Gum bleeding | 11 | 6 | 7,5 |
| 16 | 12 | Vomiting | 7 | 5 | 6,25 |
| 17 | 50 | Bitter taste | 8 | 5 | 6,25 |
| 18 | 6 | Pruritus | 4 | 4 | 5 |
| 19 | 16 | Sneezing | 4 | 4 | 5 |
| 20 | 21 | Palate Itching | 4 | 4 | 5 |
| 21 | 27 | Vertigo | 4 | 4 | 5 |
| 22 | 32 | Alergy in Body | 5 | 4 | 5 |
| 23 | 33 | Itching | 5 | 4 | 5 |
| 24 | 46 | Abdominal pain | 7 | 4 | 5 |
| 25 | 3 | Induration | 3 | 3 | 3,75 |
| 26 | 26 | Anemia | 4 | 3 | 3,75 |
| 27 | 44 | Constipation | 4 | 3 | 3,75 |
| 28 | 49 | Coughing | 3 | 3 | 3,75 |
| 29 | 63 | Frequent Urination | 4 | 3 | 3,75 |

**Annex 3.1.A.** Adverse events (AE) during PegIFN treatment, No. of reports per AE (Total), No. of patients reporting each AE (Pat /AE) and % of patients reporting the AE (%).

| **No.** | **AE-ID** | **Adverse Event** | **Total (N)** | **Pat/AE** | **%** |
| --- | --- | --- | --- | --- | --- |
| 30 | 5 | Infiltration | 2 | 2 | 2,5 |
| 31 | 36 | Weight loss | 4 | 2 | 2,5 |
| 32 | 38 | Chest pain | 3 | 2 | 2,5 |
| 33 | 43 | Conc urine | 3 | 2 | 2,5 |
| 34 | 62 | Dysentry | 6 | 2 | 2,5 |
| 35 | 65 | Diarrhea | 2 | 2 | 2,5 |
| 36 | 7 | Slight Fever | 1 | 1 | 1,25 |
| 37 | 18 | Nasal Itching | 1 | 1 | 1,25 |
| 38 | 23 | Odynophagia | 1 | 1 | 1,25 |
| 39 | 34 | Eye infection | 1 | 1 | 1,25 |
| 40 | 39 | Osteo problem | 1 | 1 | 1,25 |
| 41 | 40 | Backbone pain | 2 | 1 | 1,25 |
| 42 | 42 | Anorexia | 1 | 1 | 1,25 |
| 43 | 45 | Respiratory disorders | 1 | 1 | 1,25 |
| 44 | 47 | Urticaria | 1 | 1 | 1,25 |
| 45 | 48 | Eye pain | 1 | 1 | 1,25 |
| 46 | 51 | Sweating | 2 | 1 | 1,25 |
| 47 | 52 | Dehydratation | 1 | 1 | 1,25 |
| 48 | 53 | Appetite | 1 | 1 | 1,25 |
| 49 | 54 | Hand infection | 1 | 1 | 1,25 |
| 50 | 55 | Sense less | 1 | 1 | 1,25 |
| 51 | 56 | Acidity | 2 | 1 | 1,25 |
| 52 | 57 | Eye problem | 1 | 1 | 1,25 |
| 53 | 59 | High blood pressure | 2 | 1 | 1,25 |
| 54 | 60 | Neck pain | 1 | 1 | 1,25 |
| 55 | 61 | Blood Esputum | 1 | 1 | 1,25 |
| 56 | 64 | Low blood pressure | 1 | 1 | 1,25 |
| 57 | 66 | Teeth pain | 1 | 1 | 1,25 |
| 58 | 5 | Infiltration | 2 | 2 | 2,5 |

**Annex 3.1.A.** Adverse events during PegIFN treatment, No. of reports per AE (Total), No. of patients reporting each AE (Pat /AE) and % of patients reporting the AE (%), 2^nd^ part.

**Annex 3.2.A.** Adverse events reported during PegIFN administration, weekly report of the number of cases reported during the 48 weeks of product administration, first part weeks 01-24. Only adverse events presented by more than 5% of patients were taken into account.

| **AE-ID** | **Week**  **AE** | **W1** | **W2** | **W3** | **W4** | **W5** | **W6** | **W7** | **W8** | **W9** | **W10** | **W11** | **W12** | **W13** | **W14** | **W15** | **W16** | **W17** | **W18** | **W19** | **W20** | **W21** | **W22** | **W23** | **W24** |
| --- | --- | --- | --- | --- | --- | --- | --- | --- | --- | --- | --- | --- | --- | --- | --- | --- | --- | --- | --- | --- | --- | --- | --- | --- | --- |
| 8 | Fever | 68 | 19 | 11 | 9 | 4 | 7 | 6 | 6 | 5 | 8 | 4 | 5 | 3 | 4 | 1 | 1 | 5 | 6 | 5 | 3 | 4 | 3 | 3 | 2 |
| 13 | Weakness | 7 | 14 | 9 | 10 | 9 | 12 | 14 | 12 | 9 | 6 | 10 | 9 | 8 | 2 | 6 | 5 | 7 | 4 | 2 | 4 | 4 | 1 | 1 | 2 |
| 9 | General Malaise | 29 | 24 | 15 | 12 | 13 | 10 | 9 | 4 | 6 | 5 | 8 | 1 | 3 | 5 | 5 | 1 | 5 | 1 | 1 | 1 | 1 | 3 | 3 | 0 |
| 10 | Headache | 8 | 7 | 5 | 4 | 1 | 3 | 2 | 2 | 2 | 0 | 1 | 3 | 0 | 1 | 2 | 0 | 1 | 0 | 0 | 2 | 0 | 0 | 1 | 0 |
| 1 | Local Pain | 6 | 3 | 2 | 2 | 0 | 1 | 1 | 1 | 2 | 0 | 2 | 0 | 0 | 1 | 0 | 0 | 0 | 0 | 1 | 1 | 1 | 1 | 0 | 3 |
| 11 | Nausea | 3 | 1 | 2 | 1 | 1 | 1 | 1 | 0 | 1 | 1 | 1 | 1 | 0 | 0 | 0 | 0 | 0 | 2 | 1 | 0 | 0 | 0 | 1 | 0 |
| 25 | Lose Motion | 0 | 0 | 0 | 3 | 0 | 0 | 0 | 0 | 0 | 0 | 1 | 0 | 0 | 1 | 0 | 0 | 0 | 1 | 0 | 1 | 0 | 0 | 1 | 0 |
| 29 | Hairfall | 0 | 0 | 0 | 0 | 0 | 0 | 0 | 0 | 0 | 0 | 0 | 0 | 0 | 0 | 0 | 1 | 0 | 0 | 0 | 8 | 1 | 1 | 3 | 0 |
| 37 | GIT disorder | 0 | 2 | 1 | 1 | 0 | 0 | 0 | 0 | 0 | 0 | 0 | 0 | 0 | 0 | 0 | 0 | 0 | 0 | 0 | 0 | 0 | 1 | 0 | 0 |
| 28 | Fatigue | 0 | 0 | 0 | 1 | 0 | 0 | 1 | 1 | 0 | 1 | 1 | 0 | 1 | 0 | 1 | 0 | 0 | 0 | 0 | 1 | 0 | 0 | 1 | 0 |
| 30 | Ansiety | 0 | 0 | 2 | 2 | 2 | 1 | 0 | 1 | 0 | 0 | 0 | 0 | 0 | 0 | 0 | 0 | 0 | 0 | 0 | 1 | 0 | 0 | 0 | 1 |
| 35 | Dyspepsia | 0 | 1 | 1 | 1 | 2 | 0 | 0 | 0 | 0 | 0 | 0 | 0 | 0 | 0 | 0 | 0 | 0 | 0 | 0 | 0 | 0 | 0 | 0 | 0 |
| 58 | Gum bleeding | 0 | 1 | 0 | 0 | 0 | 0 | 1 | 0 | 0 | 0 | 0 | 0 | 0 | 0 | 0 | 0 | 0 | 0 | 0 | 0 | 1 | 1 | 1 | 0 |
| 31 | Aphtous Ulcer | 2 | 0 | 0 | 0 | 1 | 1 | 0 | 0 | 0 | 0 | 0 | 0 | 0 | 0 | 0 | 0 | 0 | 0 | 0 | 0 | 0 | 0 | 0 | 0 |
| 41 | Skin Rash | 0 | 1 | 0 | 0 | 0 | 1 | 0 | 1 | 2 | 0 | 0 | 0 | 0 | 1 | 0 | 0 | 0 | 0 | 0 | 0 | 0 | 0 | 0 | 0 |
| 12 | Vomiting | 2 | 0 | 0 | 0 | 0 | 0 | 0 | 0 | 0 | 1 | 0 | 1 | 0 | 0 | 0 | 0 | 0 | 0 | 0 | 0 | 0 | 0 | 0 | 0 |
| 50 | Bitter taste | 0 | 1 | 1 | 1 | 0 | 2 | 0 | 0 | 0 | 0 | 0 | 0 | 0 | 0 | 1 | 1 | 0 | 0 | 0 | 0 | 0 | 0 | 0 | 0 |

**Annex 3.2.A.** Adverse events reported during PegIFN administration, weekly report of the number of cases reported during the 48 weeks of product administration, 2^nd^ part weeks 25-48. Only adverse events presented by more than 5% of patients were taken into account.

| **AE-ID** | **Week**  **AE** | **W25** | **W26** | **W27** | **W28** | **W29** | **W30** | **W31** | **W32** | **W33** | **W34** | **W35** | **W36** | **W37** | **W38** | **W39** | **W40** | **W41** | **W42** | **W43** | **W44** | **W45** | **W46** | **W47** | **W48** |
| --- | --- | --- | --- | --- | --- | --- | --- | --- | --- | --- | --- | --- | --- | --- | --- | --- | --- | --- | --- | --- | --- | --- | --- | --- | --- |
| 8 | Fever | 4 | 6 | 3 | 2 | 0 | 3 | 2 | 0 | 1 | 1 | 1 | 1 | 2 | 6 | 1 | 1 | 5 | 0 | 3 | 3 | 1 | 3 | 5 | 1 |
| 13 | Weakness | 1 | 4 | 4 | 0 | 0 | 1 | 0 | 3 | 0 | 1 | 2 | 2 | 4 | 5 | 15 | 11 | 15 | 11 | 7 | 5 | 10 | 13 | 10 | 26 |
| 9 | General Malaise | 2 | 2 | 1 | 0 | 0 | 0 | 2 | 0 | 1 | 0 | 1 | 3 | 2 | 0 | 1 | 1 | 4 | 0 | 1 | 0 | 2 | 3 | 3 | 1 |
| 10 | Headache | 1 | 0 | 3 | 0 | 0 | 1 | 0 | 1 | 0 | 0 | 1 | 0 | 0 | 1 | 1 | 1 | 0 | 0 | 1 | 0 | 1 | 0 | 1 | 2 |
| 1 | Local Pain | 1 | 0 | 0 | 0 | 0 | 1 | 1 | 3 | 0 | 1 | 0 | 0 | 0 | 0 | 0 | 0 | 2 | 1 | 0 | 0 | 0 | 1 | 0 | 2 |
| 11 | Nausea | 0 | 1 | 0 | 0 | 0 | 0 | 0 | 0 | 0 | 0 | 0 | 0 | 0 | 0 | 3 | 0 | 1 | 1 | 0 | 0 | 1 | 0 | 0 | 0 |
| 25 | Lose Motion | 0 | 0 | 0 | 0 | 0 | 1 | 0 | 0 | 0 | 0 | 0 | 0 | 1 | 2 | 0 | 0 | 0 | 0 | 0 | 0 | 0 | 0 | 0 | 1 |
| 29 | Hairfall | 0 | 0 | 1 | 0 | 0 | 0 | 0 | 0 | 0 | 0 | 0 | 0 | 0 | 0 | 0 | 1 | 0 | 0 | 0 | 0 | 0 | 0 | 0 | 0 |
| 37 | GIT disorder | 1 | 1 | 0 | 3 | 0 | 0 | 0 | 0 | 0 | 0 | 0 | 0 | 0 | 0 | 0 | 0 | 0 | 0 | 0 | 0 | 0 | 0 | 0 | 2 |
| 28 | Fatigue | 0 | 0 | 0 | 0 | 0 | 0 | 0 | 0 | 0 | 0 | 0 | 0 | 0 | 0 | 0 | 0 | 1 | 0 | 0 | 0 | 0 | 0 | 0 | 0 |
| 30 | Ansiety | 0 | 0 | 0 | 0 | 0 | 0 | 0 | 0 | 0 | 0 | 0 | 0 | 0 | 0 | 0 | 0 | 0 | 0 | 0 | 0 | 0 | 0 | 0 | 0 |
| 35 | Dyspepsia | 0 | 0 | 0 | 0 | 0 | 0 | 0 | 0 | 0 | 0 | 0 | 0 | 0 | 2 | 0 | 1 | 2 | 0 | 0 | 0 | 0 | 0 | 0 | 0 |
| 58 | Gum bleeding | 0 | 2 | 2 | 0 | 0 | 0 | 0 | 0 | 0 | 0 | 0 | 0 | 0 | 0 | 0 | 0 | 0 | 0 | 0 | 0 | 1 | 0 | 0 | 1 |
| 31 | Aphtous Ulcer | 0 | 0 | 0 | 0 | 0 | 0 | 0 | 0 | 0 | 0 | 0 | 0 | 0 | 0 | 0 | 0 | 0 | 1 | 0 | 0 | 0 | 0 | 0 | 1 |
| 41 | Skin Rash | 0 | 0 | 0 | 0 | 0 | 0 | 0 | 0 | 0 | 0 | 0 | 0 | 0 | 0 | 0 | 0 | 0 | 0 | 0 | 0 | 0 | 0 | 0 | 0 |
| 12 | Vomiting | 0 | 0 | 1 | 0 | 0 | 0 | 0 | 0 | 0 | 0 | 0 | 1 | 0 | 0 | 0 | 0 | 0 | 0 | 0 | 0 | 1 | 0 | 0 | 0 |
| 50 | Bitter taste | 0 | 0 | 0 | 0 | 0 | 0 | 0 | 0 | 0 | 0 | 0 | 0 | 0 | 0 | 0 | 1 | 0 | 0 | 0 | 0 | 0 | 0 | 0 | 0 |

| **No.** | **AE-ID** | **Adverse Event** | **Total (N)** | **Pat/AE** | **%** |
| --- | --- | --- | --- | --- | --- |
| 1 | 8 | Fever | 57 | 35 | 44,87 |
| 2 | 13 | Weakness | 40 | 21 | 26,92 |
| 3 | 10 | Headache | 23 | 16 | 20,51 |
| 4 | 1 | Local Pain | 19 | 15 | 19,23 |
| 5 | 9 | General Malaise | 17 | 13 | 16,67 |
| 6 | 11 | Nausea | 12 | 8 | 10,26 |
| 7 | 12 | Pruritus | 6 | 4 | 5,13 |
| 8 | 25 | Loose Motion | 4 | 4 | 5,13 |
| 9 | 37 | GIT disorder | 4 | 4 | 5,13 |
| 10 | 7 | Slight fever | 4 | 4 | 5,13 |
| 11 | 27 | Vertigo | 4 | 4 | 5,13 |
| 12 | 32 | Allergy in body | 3 | 3 | 3,85 |
| 13 | 43 | Conc urine | 3 | 3 | 3,85 |
| 14 | 4 | Abcess | 2 | 2 | 2,56 |
| 15 | 44 | Constipation | 2 | 2 | 2,56 |
| 16 | 16 | Sneezing | 2 | 2 | 2,56 |
| 17 | 33 | Itching | 1 | 1 | 1,28 |
| 18 | 65 | Diarrhea | 1 | 1 | 1,28 |
| 19 | 30 | Ansiety | 1 | 1 | 1,28 |
| 20 | 41 | Skin rash | 1 | 1 | 1,28 |
| 21 | 69 | Heart burning | 1 | 1 | 1,28 |
| 22 | 50 | Bitter taste | 1 | 1 | 1,28 |
| 23 | 68 | Scarbia | 1 | 1 | 1,28 |
| 24 | 67 | Black stool | 1 | 1 | 1,28 |

**Annex 3.1.B.** Adverse events during NASVAC treatment, No. of reports per AE (Total), No. of patients reporting each AE (Pat /AE) and % of patients reporting the AE (%).

**Annex 3.2.B.** Adverse events reported during NASVAC administration, bi-monthly report of the number of cases reported during the 10 product administrations. Only adverse events presented by more than 5% of patients were taken into account.

| **AE-ID** | **Week**  **AE** | **W0** | **W2** | **W4** | **W6** | **W8** | **W12** | **W14** | **W16** | **W18** | **W20** |
| --- | --- | --- | --- | --- | --- | --- | --- | --- | --- | --- | --- |
| 8 | Fever | 7 | 3 | 4 | 7 | 2 | 15 | 6 | 6 | 5 | 2 |
| 13 | Weakness | 5 | 2 | 7 | 9 | 2 | 4 | 1 | 4 | 4 | 2 |
| 10 | Headache | 7 | 3 | 2 | 6 | 2 | 1 | 0 | 0 | 1 | 1 |
| 1 | Local Pain | 0 | 0 | 0 | 0 | 0 | 13 | 3 | 1 | 1 | 1 |
| 9 | General Malaise | 3 | 0 | 4 | 2 | 0 | 2 | 1 | 1 | 1 | 0 |
| 11 | Nausea | 3 | 2 | 3 | 4 | 0 | 0 | 0 | 0 | 0 | 0 |
| 12 | Pruritus | 3 | 1 | 1 | 1 | 0 | 0 | 0 | 0 | 0 | 0 |
| 25 | Loose Motion | 2 | 1 | 0 | 0 | 0 | 1 | 0 | 0 | 0 | 0 |
| 37 | GIT disorder | 0 | 1 | 0 | 3 | 0 | 0 | 0 | 0 | 0 | 0 |
| 7 | Slight fever | 0 | 0 | 4 | 0 | 0 | 0 | 0 | 0 | 0 | 0 |
| 27 | Vertigo | 1 | 2 | 0 | 0 | 0 | 0 | 1 | 0 | 0 | 0 |
